# Supplementary figures and images for: Genome-wide identification and expression analysis of the 14-3-3 gene family in soybean (Glycine max)
Source: PeerJ. 2019 Dec 6;7:e7950. doi: 10.7717/peerj.7950 (PMC6901008; doi:10.7717/peerj.7950)

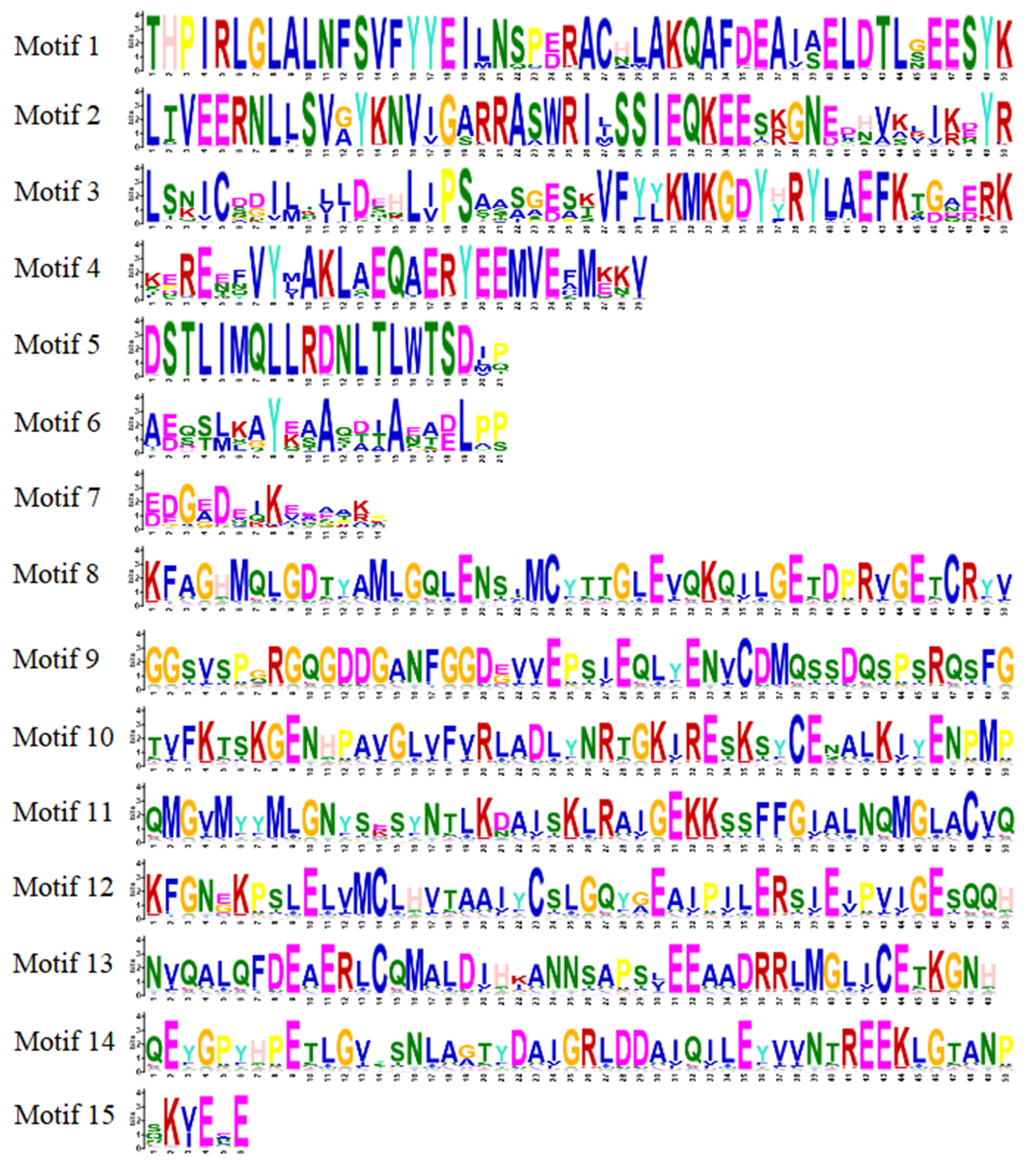

Supplement: Figure S1 [file peerj-07-7950-s001.png]

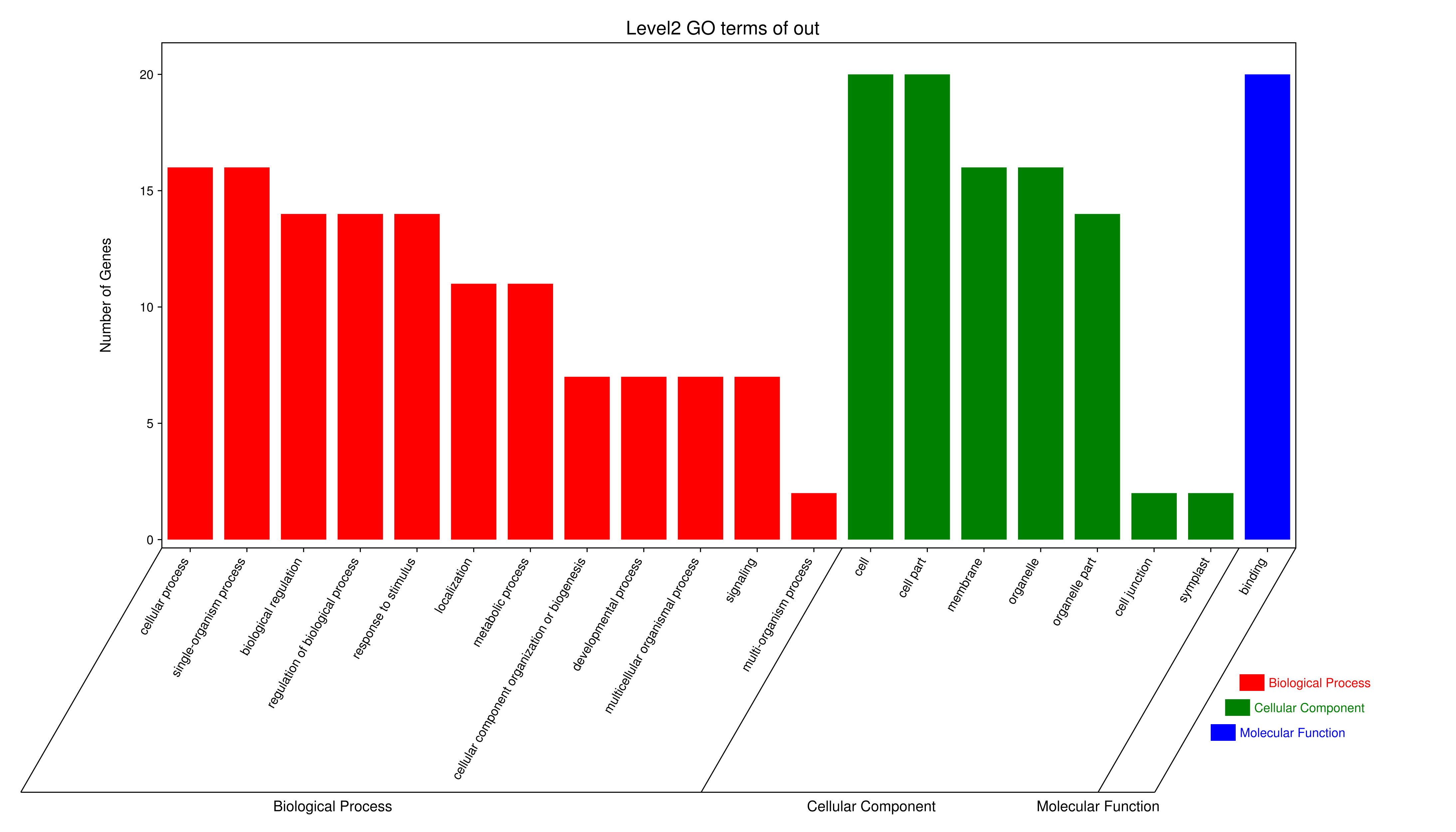

Supplement: Figure S2 [file peerj-07-7950-s002.png]
