# Supplementary material for: Genome-wide identification and expression analysis of the 14-3-3 gene family in soybean (Glycine max)
Source: PeerJ. 2019 Dec 6;7:e7950. doi: 10.7717/peerj.7950 (PMC6901008; doi:10.7717/peerj.7950)
Supplement: Table S1 [file peerj-07-7950-s003.docx]

| Table S1 List of primers used in qRT-PCR | | |
| --- | --- | --- |
| Gene name | Forward primer (5'-3') | Reverse primer(5'-3') |
| GmGF14a | TTGCGGAGTTGGACACCC | CCTCAGGCAAATCAGAGGTCCAG |
| GmGF14b | CAACCTTACTCTCTGGACTTCCG | CAATGCTCAGGCTCAGAAGGT |
| GmGF14c | TAGCACTTTGATCATGCAACTTCTT | CACCTTTGGGTGCGGCTTC |
| GmGF14d | CGAGTTTAAGGTTGGGGACGAA | GATGGGGTGAGTAGGGGGA |
| GmGF14e | AGGTTGCCATAGCTCATCCA | ATCCTGTTTCGGTGCTGCTT |
| GmGF14f | AGTTCTGATCAGTCGCCGTC | GCTTCGAAGATTTTCCATGCAC |
| GmGF14g | AAAGGGAACGATGTGAGCGT | TCACCGGACTTGAACTCTGC |
| GmGF14h | AACCTCGTACCCTCCGTCTT | GGGAAGATCCCCAGAAGCAAT |
| GmGF14i | ATACAAAGCCGCACAGGACA | TGCTTCTTTAATTTCATCTGCATGT |
| GmGF14j | GAGTTCCGATCAGTCGCCAT | CTTGTAGCTGCAGGCTGAAC |
| GmGF14k | AGGTCGAAGAGGAACTGTCCA | GTCGGTCTTGAACTCGGCTA |
| GmGF14l | GCTGAACGTGGAATTGACGG | ACCAGCAGAGCTAGAGGGAA |
| GmGF14m | GGAGGTTGAGGAGTTGACGG | AGCGGAAGGAATGAGGTTGG |
| GmGF14n | TGGCAGAATTCAAGGCTGGT | CCTCACCCTCTTCGGGGATA |
| GmGF14o | TGATGCTGTCTCAGACCTGG | TCTCCCCTGGTTGTGCTTTC |
| GmGF14p | TCGGAGCTGGATACCCTGAA | TCTCCTTGATCAACCCTGGC |
| GmGF14q | AGCCTGTCATCTTGCGAAGC | ACTTTCTGGGCATCTTCTCCATC |
| GmGF14r | AGCGACATTGCACACTAACAC | TTGAGGAGATGATGCGCCAG |
| GmGF14t | CCGCATCTCCCGAGAGTAAA | CCAGCCTAATGGGGTGAGTG |
