# Supplementary material for: Genome-wide identification and expression analysis of the 14-3-3 gene family in soybean (Glycine max)
Source: PeerJ. 2019 Dec 6;7:e7950. doi: 10.7717/peerj.7950 (PMC6901008; doi:10.7717/peerj.7950)
Supplement: Table S2 [file peerj-07-7950-s004.docx]

| **Table S2 The sequence information of GmGF14 genes** | |
| --- | --- |
| gene name | sequence |
| GmGF14a | MSAEKERETQVYLAKLAEQAERYEEMVECMKKVAKLDLDLTVEERNLLSVGYKNVIGARRASWRIMSSIEQKEESKGNEHNVKLIKSYCQKVEEELSKICGDILTIIDQHLIPSSASAEASVFYYKMKGDYFRYLAEFKTDQERKEAAEQSLKGYEAASATANTDLPSTHPIRLGLALNFSVFYYEIMNSPERACHLAKQAFDEAIAELDTLSEESYKDSTLIMQLLRDNLTLWTSDLPEDGGEDSIKAEETKPSEPEH |
| GmGF14b | MSVEKERETQVYLAKLAEQAERYEEMVECMKKVAKLDLDLTVEERNLLSVGYKNVIGARRASWRIMSSIEQKEESKGNEHNVKLIKSYCQKVEEELSKICGDILTIIDQHLIPSSGSAEASVFYYKMKGDYFRYLAEFKTDQERKEAAEQSLKGYEAASATANTDLPSTHPIRLGLALNFSVFYYEIMNSPERACHLAKQAFDEAIAELDTLSEESYKDSTLIMQLLRDNLTLWTSDLPEDGGEDNIKAEEAKPSEPEH |
| GmGF14c | MAASAPTPREEFVYMAKLAEQAERYEEMVEFMEKVSASAESEELTVEERNLLSVAYKNVIGARRASWRIISSIEQKEESRGNEDHVAVIRDYRSKIEAELSNICDGILKLLDTRLVPSAASGDSKVFYLKMKGDYHRYLAEFKTGADRKEAAESTLSAYKAAQDIANTELPPTHPIRLGLALNFSVFYYEILNSPDRACSLAKQAFDEAIAELDTLGEESYKDSTLIMQLLRDNLTLWTSDMQDDGADEIKEAAPKGDGEQN |
| GmGF14d | MAAAEGLNREQYVYLAKLSEQAERYEEMVEFMQKVVVGSTPASELTVEERNLLSVAYKNVIGSLRAAWRIVSSIEQKEEGRKNDDHVSLVKHYRSKVENELTQVCASILSLLDSNLVPSASASESKVFYLKMKGDYHRYLAEFKVGDERKTATEDTMLSYKAAQDIASADLPPTHPIRLGLALNFSVFYYEILNQSDKACAMAKQAFEEAIAELDTLGEESYKDSTLIMQLLRDNLTLWTSDVQDQLDEP |
| GmGF14e | MAAAPSPREENVYMAKLAEQAERYEEMVEFMEKVSAAADNEELTVEERNLLSVAYKNVIGARRASWRIISSIEQKEESRGNEDHVSVIRDYRSKIESELSNICDGILKLLDSRLIPSASSGDSKVFYLKMKGDYHRYLAEFKTGAERKEAAESTLSAYKAAQDIANAELPPTHPIRLGLALNFSVFYYEILNSPDRACNLAKQAFDEAIAELDTLGEESYKDSTLIMQLLRDNLTLWTSDMQVLILGCHSSSITSFFISLFCVVDLLLWFQDDGADEIKEAAPKQDDQ |
| GmGF14f | MKELNMPGIVTHGVQDEGGSNEMNGDQDSPFKEPLNLVKSPRGGSVSPGRGQGDDGANFGGDGVVEPSIEQLYENVCDMQSSDQSPSRQSFGSDGDESRIDSELRHLVGGRMREVEIMEEECGEGKEPEGSSSSEISCAWKIFEALNSQVESDNTLPKLTSKGRSPLSKAPIPRKNGKPLRKPIGGVTGVKNTKNSPIGKSVSQNRVESMAESALEKPERAPVLLKQARDLISSGDNPQKALDLALQAMELFEKFGNGKPSLELVMCLHVTAAIYCSLGQYAEAIPILERSIEIPVIGESQQHALAKFAGHMQLGDTYAMLGQLENSIMCYTTGLEVQKQILGETDPRVGETCRYVAEANVQALQFDEAERLCQMALDIHKANNSAPSVEEAADRRLMGLICETKGNHETALEHLVLASMAMVNNGQEAEVASVDCSIGDTYLSLSRYDEAAFAYQKALTVFKTSKGENHPAVGLVFVRLADLYNRTGKIRESKSYCENALKIYENPMPGVPLEEIASGLTNISTIYESMNELEQALKLLQKALEIYSDTPGQQSTIAGIEAQMGVMYYMLGNYSESYNTLKDAISKLRAIGEKKSSFFGIALNQMGLACVQRYALSEATELFEEAKSILEQEYGPYHPETLGVYSNLAGTYDAIGRLDDAIQILEYVVNTREEKLGTANPEVDDEKRRLGELLKEAGRVRSRKARSLENLLDGNAHAANNVVIRA |
| GmGF14g | MASTKERENFVYVAKLAEQAERYEEMVEAMKNVAKLNVELTVEERNLLSVGYKNVVGARRASWRILSSIEQKEEAKGNDVSVKRIKEYRLKVESELSNICSDIMTVIDEYLIPSSSSGEPSVFFYKMKGDYYRYLAEFKSGDERKEAADHSMKAYQLASTTAEAELASTHPIRLGLALNFSVFYYEILNSPERACHLAKQAFDEAISELDTLSEESYKDSTLIMQLLRDNLTLWTSDIPEDGAEEQKVDSARAAGGDDA |
| GmGF14h | MAGAEGLNREQYVYLAKLSEQAERYEEMVEFMQKVVVGWTPASELTVEERNLLSVAYKNVIGSLRAAWRIVSSIEQKEEGRKNDDHVSLVKHYRSKVENELTQVCASILNLLDSNLVPSVFASESKVFYLKMKGDYHRYLAEFKVGDERKTAAEDTMLSYKAAQDIASGDLPPTHPIRLGLALNFSVFYYEILNQSDKACAMAKQAFEEAIAELDTLGEESYKDSTLIMQLLRDNLTLWTSDVQDQLDEP |
| GmGF14i | MAAAPSPREENVYMAKLAEQAERYEEMVEFMEKVSAAADNEELTVEERNLLSVAYKNVIGARRASWRIISSIEQKEESRGNEDHVSVIRDYRSKIESELSNICDGILKLLDSRLIPSASSGDSKVFYLKMKGDYHRYLAEFKTGAERKEAAESTLSAYKAAQDIANAELPPTHPIRLGLALNFSVFYYEILNSPDRACNLAKQAFDEAIAELDTLGEESYKDSTLIMQLLRDNLTLWTSDMQMKLKKQHRNRMTSKNILLLIRIELLLHIFLKGEGVCC |
| GmGF14j | MPGIVRNGVHHDEGGSNDLNGDHDSTSKEPLNLVKSPRGGSVSPQRGQGDDGANFGGDEVVEPSIEQLYENVCDMQSSDQSPSRQSFGSDGDESRIDSELRHLVGGRMREVEIMEEEVGEEKGLPEGSSSSEISSALGGLSNDKKLNQVDEIQEVQPAATSSGSSEKSIKASISMVGPDNTSPKSTSKGKIPLSKAPIPRNNGKPLRKQISGATTGVKTTKNSPMGKSVSRNRAESTAESALEKPERAPVLLKQARDLISSGDNPHKALDLALQAMKLFEKFGNEKPSLELVMCLHVTAAIYCSLGQYGEAIPILERSIEVPVIGESQQHALAKFAGHMQLGDTYAMLGQLENSTMCYTTGLEVQKQILGETDPRVGETCRYVAEANVQALQFDEAERLCQMALDIHIANNSAPSLEEAADRRLMGLICETKGNHETALEHLVLASMAMVSNDQEAEVASVDCSIGDTYLSLSRYDEAVFAYQKALTVFKTSKGENHPAVGLVFVRLADLYNRTGKIRESKSYCESALKIYENPMPGIPPEEIASGLTNISTIYESMNELEHALKLLQKALEIYNDTPGQQSTIAGIEAQMGVMYYMLGNYSKSYNTLKNAISKLRAIGEKKSSFFGIALNQMGLACVQCYALSEATELFEEAKSILEQEYGPYHPETLGVSSNLAATYDAIGRLDDAIQILEYVVNTREEKLGTANPEVDDEKRRLGELLKEAGRVRSRKTRSLENLLDGNAHAANNVVIRA |
| GmGF14k | MSTEKERETQVYLAKLSEQAERYEEMVECMKTIAKLDLELTVEERNLLSVGYKNVIGARRASWRIMSSIEQKEESKGNESNAKLIKNYRQKVEEELSKICSDILSIIDQHLVPSSTSGEATVFYYKMKGDYYRYLAEFKTDQDRKEAAEQSLKGYEAALATASTDLPSTHPIRLGLALNFSVFYYEILNSPERACHLAKQAFDEAIAELDTLSEESYKDSTLIMQLLRDNLTLWTSDLPEDGGDEIKTEEVKPAETVEH |
| GmGF14l | MASTKERENFVYTAKLAEQAERYEEMVEAMKNVAKLNVELTVEERNLLSVGYKNVVGARRASWRILSSIEQKEEAKGNDVSVKRIKEYRQKVESELSNICSDIMTVIDEHLIPSSSAGEPSVFFYKMKGDYYRYLAEFKSGDERKEAADHSMKAYQSASTTAEAELPPTHPIRLGLALNFSVFYYEILNSPERACHLAKQAFDEAISELDTLSEESYKDSTLIMQLLRDNLTLWTSDIPEEGAEEQKVDSARAAGGDNA |
| GmGF14m | MADSSREENVYMAKLAEQAERYEEMVEFMEKVAKTVEVEELTVEERNLLSVAYKNVIGARRASWRIISSIEQKEESRGNEDHVAIIKEYRGKIEAELSKICDGILNLLESNLIPSAASPESKVFYLKMKGDYHRYLAEFKTGAERKEAAESTLLAYKSAQDIALADLAPTHPIRLGLALNFSVFYYEILNSPDRACNLAKQAFDEAISELDTLGEESYKDSTLIMQLLRDNLTLWTSDITVSFSLSISLLLYIVNISLCV |
| GmGF14n | MAASKDRENFVYIAKLAEQAERYEEMVESMKNVANLDVELTVEERNLLSVGYKNVIGARRASWRILSSIEQKEETKGNELNAKRIKEYRQKVELELSNICNDVMTVIDEHLIPSAAAGESTVFYYKMKGDYYRYLAEFKAGNEKKEAADQSMKAYESATTAAEADLPPTHPIRLGLALNFSVFYYEILNSPERACHLAKQAFDEAISELDTLNEESYKDSTLIMQLLRDNLTLWTSDIPEDGEDAQKVNGTAKLGGGEEAE |
| GmGF14o | MTQPAMATFSKERENFVYVAKLAEQAERYDEMVDAMKKVAKLDVELSVEERNLFSVGYKNVVGSRRASWRILSSIEQKEESKGNELHVKRIRDYRNKVELELSNICSDIMIVLDEHLIPSTNIAESTVFYYKMKGDYYRYLAEFKAGNEKKEVADQSLKAYETASTTAESELQPTHPIRLGLALNFSVFYYEIMNSPERACHLAKQAFDDAVSDLDTLNEDSYKDSTLIMQLLRDNLTLWTSDIPEEGEDQKMESTTRGEDELGR |
| GmGF14p | MATFSKERENFVYVAKLAEQAERYDEMVDAMKKVAKLDVELSVEERNLFSVGYKNVVGSRRASWRILSSIEQKEDSKGNELHVKHIRDYRNKVELELSNICSDIMIILDEHLIPSTNIAESTVFYYKMKGDYYRYLAEFKAGNEKKEVADQSLKAYQTASTTAESELQPTHPIRLGLALNFSVFYYEILNSPERACHLAKQAFDDAVSELDTLNEDSYKDSTLIMQLLRDNLTLWTSDIPEEGEDLKMESAARVDQGEDELGR |
| GmGF14q | MTASKDRENFVYIAKLAEQAERYEEMVESMKNVANLDVELTVEERNLLSVGYKNVIGARRASWRILSSIEQKEETKGNELNAKRIKEYRQKVELELSNICNDVMRVIDEHLIPSAAAGESTVFYYKMKGDYYRYLAEFKSGNEKKEAADQSMKAYESATAAAEADLPPTHPIRLGLALNFSVFYYEILNSPERACHLAKQAFDEAISELDTLNEESYKDSTLIMQLLRDNLTLWTSDIPEDGEDAQKVNGTAKLGGGEDAE |
| GmGF14r | MLPSMDNTCPCMSDITQIIRKALIPCYCLYFPCIFCVSPNERATLHTNTDLVAMASAPTPREEFVYMAKLAEQAERYEEMVEFMEKVSASAESEELTVEERNLLSVAYKNVIGARRASWRIISSIEQKEESRGNEDHVAVIRDYRSKIEAELSNICDGILKLLDTRLVPSAASGDSKVFYLKMKGDYHRYLAEFKTGADRKEAAESTLSAYKAAQDIANTELPPTHPIRLGLALNFSVFYYEILNSPDRACSLAKQAFDEAIAELDTLGEESYKDSTLIMQLLRDNLTLWTSDMQDDGADEIKEAAPKGDGEQN |
| GmGF14s | MASAPTPCEEFVYMAKLVEQVKHYEEMVEFMEKVFASTESEELTVDERNLLSVAYKNMIGKEDHLVVIRNYRSKIKVELSNIYDKILKLLDTHLVPSLTSGDSKVFYLKMKGAYHRYFTEFKIDANRKEAVESPLSTYKAAQICFFFFSFDFLDFYFVM |
| GmGF14t | MSDSSREENVYMAKLAEQAERYEEMVEFMEKVAKTVEVEELTVEERNLLSVAYKNVIGARRASWRIISSIEQKEESRGNEDHVAIIKEYRGKIEAELSKICDGILNLLESNLIPSAASPESKVFYLKMKGDYHRYLAEFKTGAERKEAAESTLLAYKSAQDIALADLAPTHPIRLGLALNFSVFYYEILNSPDRACNLAKQAFDEAISELDTLGEESYKDSTLIMQLLRDNLTLWTSDITDDAGDEIKETSKQQPGE |
| GmGF14u | MSTEKERETQVYLAKLSEQAERYEEMVECMKTIAKLDLELTVEERNLLSVGYKNVIGARRASWRIMSSIEQKEESKGNESNAKLIKNYRQKVEEELSKICSDILSIIDQHLVPSSTSGEATVFYYKMKGDYYRYLAEFKTDQDRKEAAEQSLKGYEAALATASTELPSTHPIRLGLALNFSVFYYEILNSPERACHLAKQAFDEAIAELDTLSEESYKDSTLIMQLLRDNLTLWTSDLPEDGGDEIKTEEAKPAETSEHS |
| GmGF14v | MLKCSACKKGDYYCYLAEFKSGNEKKEAVDQSMKAYESATTAAEVDLPPTHPIRFGLALNFLVFYYEILP |
