# Supplementary material for: Genome-wide identification and expression analysis of the 14-3-3 gene family in soybean (Glycine max)
Source: PeerJ. 2019 Dec 6;7:e7950. doi: 10.7717/peerj.7950 (PMC6901008; doi:10.7717/peerj.7950)
Supplement: Table S3 [file peerj-07-7950-s005.docx]

| **Table S3 The GFF file for GF14 genes** | | | | | | | | |
| --- | --- | --- | --- | --- | --- | --- | --- | --- |
| Chr01 | phytozomev10 | gene | 7642485 | 7646277 | . | + | . | ID=Glyma.01G058000.Wm82.a2.v1;Name=Glyma.01G058000;ancestorIdentifier=Glyma01g07070.v1.1 |
| Chr01 | phytozomev10 | mRNA | 7642485 | 7646277 | . | + | . | ID=Glyma.01G058000.1.Wm82.a2.v1;Name=Glyma.01G058000.1;pacid=30542937;longest=1;ancestorIdentifer=Glyma01g07070.1.v1.1;Parent=Glyma.01G058000.Wm82.a2.v1 |
| Chr01 | phytozomev10 | exon | 7642485 | 7642783 | . | + | . | ID=Glyma.01G058000.1.Wm82.a2.v1.exon.1;Parent=Glyma.01G058000.1.Wm82.a2.v1;pacid=30542937 |
| Chr01 | phytozomev10 | 5p_UTR | 7642485 | 7642710 | . | + | . | ID=Glyma.01G058000.1.Wm82.a2.v1.5p_UTR.1;Parent=Glyma.01G058000.1.Wm82.a2.v1;pacid=30542937 |
| Chr01 | phytozomev10 | CDS | 7642711 | 7642783 | . | + | 0 | ID=Glyma.01G058000.1.Wm82.a2.v1.CDS.1;Parent=Glyma.01G058000.1.Wm82.a2.v1;pacid=30542937 |
| Chr01 | phytozomev10 | exon | 7643987 | 7644293 | . | + | . | ID=Glyma.01G058000.1.Wm82.a2.v1.exon.2;Parent=Glyma.01G058000.1.Wm82.a2.v1;pacid=30542937 |
| Chr01 | phytozomev10 | CDS | 7643987 | 7644293 | . | + | 2 | ID=Glyma.01G058000.1.Wm82.a2.v1.CDS.2;Parent=Glyma.01G058000.1.Wm82.a2.v1;pacid=30542937 |
| Chr01 | phytozomev10 | exon | 7644726 | 7644813 | . | + | . | ID=Glyma.01G058000.1.Wm82.a2.v1.exon.3;Parent=Glyma.01G058000.1.Wm82.a2.v1;pacid=30542937 |
| Chr01 | phytozomev10 | CDS | 7644726 | 7644813 | . | + | 1 | ID=Glyma.01G058000.1.Wm82.a2.v1.CDS.3;Parent=Glyma.01G058000.1.Wm82.a2.v1;pacid=30542937 |
| Chr01 | phytozomev10 | exon | 7644923 | 7645032 | . | + | . | ID=Glyma.01G058000.1.Wm82.a2.v1.exon.4;Parent=Glyma.01G058000.1.Wm82.a2.v1;pacid=30542937 |
| Chr01 | phytozomev10 | CDS | 7644923 | 7645032 | . | + | 0 | ID=Glyma.01G058000.1.Wm82.a2.v1.CDS.4;Parent=Glyma.01G058000.1.Wm82.a2.v1;pacid=30542937 |
| Chr01 | phytozomev10 | exon | 7645122 | 7645270 | . | + | . | ID=Glyma.01G058000.1.Wm82.a2.v1.exon.5;Parent=Glyma.01G058000.1.Wm82.a2.v1;pacid=30542937 |
| Chr01 | phytozomev10 | CDS | 7645122 | 7645270 | . | + | 1 | ID=Glyma.01G058000.1.Wm82.a2.v1.CDS.5;Parent=Glyma.01G058000.1.Wm82.a2.v1;pacid=30542937 |
| Chr01 | phytozomev10 | exon | 7645632 | 7645678 | . | + | . | ID=Glyma.01G058000.1.Wm82.a2.v1.exon.6;Parent=Glyma.01G058000.1.Wm82.a2.v1;pacid=30542937 |
| Chr01 | phytozomev10 | CDS | 7645632 | 7645678 | . | + | 2 | ID=Glyma.01G058000.1.Wm82.a2.v1.CDS.6;Parent=Glyma.01G058000.1.Wm82.a2.v1;pacid=30542937 |
| Chr01 | phytozomev10 | exon | 7645920 | 7646277 | . | + | . | ID=Glyma.01G058000.1.Wm82.a2.v1.exon.7;Parent=Glyma.01G058000.1.Wm82.a2.v1;pacid=30542937 |
| Chr01 | phytozomev10 | CDS | 7645920 | 7645925 | . | + | 0 | ID=Glyma.01G058000.1.Wm82.a2.v1.CDS.7;Parent=Glyma.01G058000.1.Wm82.a2.v1;pacid=30542937 |
| Chr01 | phytozomev10 | 3p_UTR | 7645926 | 7646277 | . | + | . | ID=Glyma.01G058000.1.Wm82.a2.v1.3p_UTR.1;Parent=Glyma.01G058000.1.Wm82.a2.v1;pacid=30542937 |
| Chr02 | phytozomev10 | gene | 11280858 | 11285984 | . | + | . | ID=Glyma.02G115900.Wm82.a2.v1;Name=Glyma.02G115900;ancestorIdentifier=Glyma02g12930.v1.1 |
| Chr02 | phytozomev10 | mRNA | 11280858 | 11285331 | . | + | . | ID=Glyma.02G115900.1.Wm82.a2.v1;Name=Glyma.02G115900.1;pacid=30507087;longest=1;ancestorIdentifer=Glyma02g12930.1.v1.1;Parent=Glyma.02G115900.Wm82.a2.v1 |
| Chr02 | phytozomev10 | exon | 11280858 | 11281556 | . | + | . | ID=Glyma.02G115900.1.Wm82.a2.v1.exon.1;Parent=Glyma.02G115900.1.Wm82.a2.v1;pacid=30507087 |
| Chr02 | phytozomev10 | 5p_UTR | 11280858 | 11281483 | . | + | . | ID=Glyma.02G115900.1.Wm82.a2.v1.5p_UTR.1;Parent=Glyma.02G115900.1.Wm82.a2.v1;pacid=30507087 |
| Chr02 | phytozomev10 | CDS | 11281484 | 11281556 | . | + | 0 | ID=Glyma.02G115900.1.Wm82.a2.v1.CDS.1;Parent=Glyma.02G115900.1.Wm82.a2.v1;pacid=30507087 |
| Chr02 | phytozomev10 | exon | 11282837 | 11283143 | . | + | . | ID=Glyma.02G115900.1.Wm82.a2.v1.exon.2;Parent=Glyma.02G115900.1.Wm82.a2.v1;pacid=30507087 |
| Chr02 | phytozomev10 | CDS | 11282837 | 11283143 | . | + | 2 | ID=Glyma.02G115900.1.Wm82.a2.v1.CDS.2;Parent=Glyma.02G115900.1.Wm82.a2.v1;pacid=30507087 |
| Chr02 | phytozomev10 | exon | 11283578 | 11283665 | . | + | . | ID=Glyma.02G115900.1.Wm82.a2.v1.exon.3;Parent=Glyma.02G115900.1.Wm82.a2.v1;pacid=30507087 |
| Chr02 | phytozomev10 | CDS | 11283578 | 11283665 | . | + | 1 | ID=Glyma.02G115900.1.Wm82.a2.v1.CDS.3;Parent=Glyma.02G115900.1.Wm82.a2.v1;pacid=30507087 |
| Chr02 | phytozomev10 | exon | 11283775 | 11283884 | . | + | . | ID=Glyma.02G115900.1.Wm82.a2.v1.exon.4;Parent=Glyma.02G115900.1.Wm82.a2.v1;pacid=30507087 |
| Chr02 | phytozomev10 | CDS | 11283775 | 11283884 | . | + | 0 | ID=Glyma.02G115900.1.Wm82.a2.v1.CDS.4;Parent=Glyma.02G115900.1.Wm82.a2.v1;pacid=30507087 |
| Chr02 | phytozomev10 | exon | 11283974 | 11284122 | . | + | . | ID=Glyma.02G115900.1.Wm82.a2.v1.exon.5;Parent=Glyma.02G115900.1.Wm82.a2.v1;pacid=30507087 |
| Chr02 | phytozomev10 | CDS | 11283974 | 11284122 | . | + | 1 | ID=Glyma.02G115900.1.Wm82.a2.v1.CDS.5;Parent=Glyma.02G115900.1.Wm82.a2.v1;pacid=30507087 |
| Chr02 | phytozomev10 | exon | 11284493 | 11284539 | . | + | . | ID=Glyma.02G115900.1.Wm82.a2.v1.exon.6;Parent=Glyma.02G115900.1.Wm82.a2.v1;pacid=30507087 |
| Chr02 | phytozomev10 | CDS | 11284493 | 11284539 | . | + | 2 | ID=Glyma.02G115900.1.Wm82.a2.v1.CDS.6;Parent=Glyma.02G115900.1.Wm82.a2.v1;pacid=30507087 |
| Chr02 | phytozomev10 | exon | 11284865 | 11285331 | . | + | . | ID=Glyma.02G115900.1.Wm82.a2.v1.exon.7;Parent=Glyma.02G115900.1.Wm82.a2.v1;pacid=30507087 |
| Chr02 | phytozomev10 | CDS | 11284865 | 11284870 | . | + | 0 | ID=Glyma.02G115900.1.Wm82.a2.v1.CDS.7;Parent=Glyma.02G115900.1.Wm82.a2.v1;pacid=30507087 |
| Chr02 | phytozomev10 | 3p_UTR | 11284871 | 11285331 | . | + | . | ID=Glyma.02G115900.1.Wm82.a2.v1.3p_UTR.1;Parent=Glyma.02G115900.1.Wm82.a2.v1;pacid=30507087 |
| Chr02 | phytozomev10 | mRNA | 11280858 | 11285984 | . | + | . | ID=Glyma.02G115900.2.Wm82.a2.v1;Name=Glyma.02G115900.2;pacid=30507086;longest=0;Parent=Glyma.02G115900.Wm82.a2.v1 |
| Chr02 | phytozomev10 | exon | 11280858 | 11281556 | . | + | . | ID=Glyma.02G115900.2.Wm82.a2.v1.exon.1;Parent=Glyma.02G115900.2.Wm82.a2.v1;pacid=30507086 |
| Chr02 | phytozomev10 | 5p_UTR | 11280858 | 11281483 | . | + | . | ID=Glyma.02G115900.2.Wm82.a2.v1.5p_UTR.1;Parent=Glyma.02G115900.2.Wm82.a2.v1;pacid=30507086 |
| Chr02 | phytozomev10 | CDS | 11281484 | 11281556 | . | + | 0 | ID=Glyma.02G115900.2.Wm82.a2.v1.CDS.1;Parent=Glyma.02G115900.2.Wm82.a2.v1;pacid=30507086 |
| Chr02 | phytozomev10 | exon | 11282837 | 11283143 | . | + | . | ID=Glyma.02G115900.2.Wm82.a2.v1.exon.2;Parent=Glyma.02G115900.2.Wm82.a2.v1;pacid=30507086 |
| Chr02 | phytozomev10 | CDS | 11282837 | 11283143 | . | + | 2 | ID=Glyma.02G115900.2.Wm82.a2.v1.CDS.2;Parent=Glyma.02G115900.2.Wm82.a2.v1;pacid=30507086 |
| Chr02 | phytozomev10 | exon | 11283578 | 11283665 | . | + | . | ID=Glyma.02G115900.2.Wm82.a2.v1.exon.3;Parent=Glyma.02G115900.2.Wm82.a2.v1;pacid=30507086 |
| Chr02 | phytozomev10 | CDS | 11283578 | 11283665 | . | + | 1 | ID=Glyma.02G115900.2.Wm82.a2.v1.CDS.3;Parent=Glyma.02G115900.2.Wm82.a2.v1;pacid=30507086 |
| Chr02 | phytozomev10 | exon | 11283775 | 11283884 | . | + | . | ID=Glyma.02G115900.2.Wm82.a2.v1.exon.4;Parent=Glyma.02G115900.2.Wm82.a2.v1;pacid=30507086 |
| Chr02 | phytozomev10 | CDS | 11283775 | 11283884 | . | + | 0 | ID=Glyma.02G115900.2.Wm82.a2.v1.CDS.4;Parent=Glyma.02G115900.2.Wm82.a2.v1;pacid=30507086 |
| Chr02 | phytozomev10 | exon | 11283974 | 11284122 | . | + | . | ID=Glyma.02G115900.2.Wm82.a2.v1.exon.5;Parent=Glyma.02G115900.2.Wm82.a2.v1;pacid=30507086 |
| Chr02 | phytozomev10 | CDS | 11283974 | 11284122 | . | + | 1 | ID=Glyma.02G115900.2.Wm82.a2.v1.CDS.5;Parent=Glyma.02G115900.2.Wm82.a2.v1;pacid=30507086 |
| Chr02 | phytozomev10 | exon | 11284493 | 11284539 | . | + | . | ID=Glyma.02G115900.2.Wm82.a2.v1.exon.6;Parent=Glyma.02G115900.2.Wm82.a2.v1;pacid=30507086 |
| Chr02 | phytozomev10 | CDS | 11284493 | 11284539 | . | + | 2 | ID=Glyma.02G115900.2.Wm82.a2.v1.CDS.6;Parent=Glyma.02G115900.2.Wm82.a2.v1;pacid=30507086 |
| Chr02 | phytozomev10 | exon | 11284865 | 11284896 | . | + | . | ID=Glyma.02G115900.2.Wm82.a2.v1.exon.7;Parent=Glyma.02G115900.2.Wm82.a2.v1;pacid=30507086 |
| Chr02 | phytozomev10 | CDS | 11284865 | 11284870 | . | + | 0 | ID=Glyma.02G115900.2.Wm82.a2.v1.CDS.7;Parent=Glyma.02G115900.2.Wm82.a2.v1;pacid=30507086 |
| Chr02 | phytozomev10 | 3p_UTR | 11284871 | 11284896 | . | + | . | ID=Glyma.02G115900.2.Wm82.a2.v1.3p_UTR.1;Parent=Glyma.02G115900.2.Wm82.a2.v1;pacid=30507086 |
| Chr02 | phytozomev10 | exon | 11285927 | 11285984 | . | + | . | ID=Glyma.02G115900.2.Wm82.a2.v1.exon.8;Parent=Glyma.02G115900.2.Wm82.a2.v1;pacid=30507086 |
| Chr02 | phytozomev10 | 3p_UTR | 11285927 | 11285984 | . | + | . | ID=Glyma.02G115900.2.Wm82.a2.v1.3p_UTR.2;Parent=Glyma.02G115900.2.Wm82.a2.v1;pacid=30507086 |
| Chr02 | phytozomev10 | gene | 39388574 | 39391014 | . | + | . | ID=Glyma.02G208700.Wm82.a2.v1;Name=Glyma.02G208700;ancestorIdentifier=Glyma02g37120.v1.1 |
| Chr02 | phytozomev10 | mRNA | 39388574 | 39391014 | . | + | . | ID=Glyma.02G208700.1.Wm82.a2.v1;Name=Glyma.02G208700.1;pacid=30510513;longest=1;ancestorIdentifer=Glyma02g37120.1.v1.1;Parent=Glyma.02G208700.Wm82.a2.v1 |
| Chr02 | phytozomev10 | exon | 39388574 | 39389227 | . | + | . | ID=Glyma.02G208700.1.Wm82.a2.v1.exon.1;Parent=Glyma.02G208700.1.Wm82.a2.v1;pacid=30510513 |
| Chr02 | phytozomev10 | 5p_UTR | 39388574 | 39388738 | . | + | . | ID=Glyma.02G208700.1.Wm82.a2.v1.5p_UTR.1;Parent=Glyma.02G208700.1.Wm82.a2.v1;pacid=30510513 |
| Chr02 | phytozomev10 | CDS | 39388739 | 39389227 | . | + | 0 | ID=Glyma.02G208700.1.Wm82.a2.v1.CDS.1;Parent=Glyma.02G208700.1.Wm82.a2.v1;pacid=30510513 |
| Chr02 | phytozomev10 | exon | 39389909 | 39390031 | . | + | . | ID=Glyma.02G208700.1.Wm82.a2.v1.exon.2;Parent=Glyma.02G208700.1.Wm82.a2.v1;pacid=30510513 |
| Chr02 | phytozomev10 | CDS | 39389909 | 39390031 | . | + | 0 | ID=Glyma.02G208700.1.Wm82.a2.v1.CDS.2;Parent=Glyma.02G208700.1.Wm82.a2.v1;pacid=30510513 |
| Chr02 | phytozomev10 | exon | 39390295 | 39390411 | . | + | . | ID=Glyma.02G208700.1.Wm82.a2.v1.exon.3;Parent=Glyma.02G208700.1.Wm82.a2.v1;pacid=30510513 |
| Chr02 | phytozomev10 | CDS | 39390295 | 39390411 | . | + | 0 | ID=Glyma.02G208700.1.Wm82.a2.v1.CDS.3;Parent=Glyma.02G208700.1.Wm82.a2.v1;pacid=30510513 |
| Chr02 | phytozomev10 | exon | 39390516 | 39391014 | . | + | . | ID=Glyma.02G208700.1.Wm82.a2.v1.exon.4;Parent=Glyma.02G208700.1.Wm82.a2.v1;pacid=30510513 |
| Chr02 | phytozomev10 | CDS | 39390516 | 39390575 | . | + | 0 | ID=Glyma.02G208700.1.Wm82.a2.v1.CDS.4;Parent=Glyma.02G208700.1.Wm82.a2.v1;pacid=30510513 |
| Chr02 | phytozomev10 | 3p_UTR | 39390576 | 39391014 | . | + | . | ID=Glyma.02G208700.1.Wm82.a2.v1.3p_UTR.1;Parent=Glyma.02G208700.1.Wm82.a2.v1;pacid=30510513 |
| Chr02 | phytozomev10 | mRNA | 39388574 | 39391014 | . | + | . | ID=Glyma.02G208700.2.Wm82.a2.v1;Name=Glyma.02G208700.2;pacid=30510514;longest=0;Parent=Glyma.02G208700.Wm82.a2.v1 |
| Chr02 | phytozomev10 | exon | 39388574 | 39389227 | . | + | . | ID=Glyma.02G208700.2.Wm82.a2.v1.exon.1;Parent=Glyma.02G208700.2.Wm82.a2.v1;pacid=30510514 |
| Chr02 | phytozomev10 | 5p_UTR | 39388574 | 39388738 | . | + | . | ID=Glyma.02G208700.2.Wm82.a2.v1.5p_UTR.1;Parent=Glyma.02G208700.2.Wm82.a2.v1;pacid=30510514 |
| Chr02 | phytozomev10 | CDS | 39388739 | 39389227 | . | + | 0 | ID=Glyma.02G208700.2.Wm82.a2.v1.CDS.1;Parent=Glyma.02G208700.2.Wm82.a2.v1;pacid=30510514 |
| Chr02 | phytozomev10 | exon | 39389909 | 39390031 | . | + | . | ID=Glyma.02G208700.2.Wm82.a2.v1.exon.2;Parent=Glyma.02G208700.2.Wm82.a2.v1;pacid=30510514 |
| Chr02 | phytozomev10 | CDS | 39389909 | 39390031 | . | + | 0 | ID=Glyma.02G208700.2.Wm82.a2.v1.CDS.2;Parent=Glyma.02G208700.2.Wm82.a2.v1;pacid=30510514 |
| Chr02 | phytozomev10 | exon | 39390295 | 39391014 | . | + | . | ID=Glyma.02G208700.2.Wm82.a2.v1.exon.3;Parent=Glyma.02G208700.2.Wm82.a2.v1;pacid=30510514 |
| Chr02 | phytozomev10 | CDS | 39390295 | 39390438 | . | + | 0 | ID=Glyma.02G208700.2.Wm82.a2.v1.CDS.3;Parent=Glyma.02G208700.2.Wm82.a2.v1;pacid=30510514 |
| Chr02 | phytozomev10 | 3p_UTR | 39390439 | 39391014 | . | + | . | ID=Glyma.02G208700.2.Wm82.a2.v1.3p_UTR.1;Parent=Glyma.02G208700.2.Wm82.a2.v1;pacid=30510514 |
| Chr04 | phytozomev10 | gene | 8158031 | 8160711 | . | + | . | ID=Glyma.04G092600.Wm82.a2.v1;Name=Glyma.04G092600;ancestorIdentifier=Glyma04g09820.v1.1 |
| Chr04 | phytozomev10 | mRNA | 8158031 | 8160711 | . | + | . | ID=Glyma.04G092600.1.Wm82.a2.v1;Name=Glyma.04G092600.1;pacid=30487935;longest=1;ancestorIdentifer=Glyma04g09820.1.v1.1;Parent=Glyma.04G092600.Wm82.a2.v1 |
| Chr04 | phytozomev10 | exon | 8158031 | 8158714 | . | + | . | ID=Glyma.04G092600.1.Wm82.a2.v1.exon.1;Parent=Glyma.04G092600.1.Wm82.a2.v1;pacid=30487935 |
| Chr04 | phytozomev10 | 5p_UTR | 8158031 | 8158222 | . | + | . | ID=Glyma.04G092600.1.Wm82.a2.v1.5p_UTR.1;Parent=Glyma.04G092600.1.Wm82.a2.v1;pacid=30487935 |
| Chr04 | phytozomev10 | CDS | 8158223 | 8158714 | . | + | 0 | ID=Glyma.04G092600.1.Wm82.a2.v1.CDS.1;Parent=Glyma.04G092600.1.Wm82.a2.v1;pacid=30487935 |
| Chr04 | phytozomev10 | exon | 8158909 | 8159031 | . | + | . | ID=Glyma.04G092600.1.Wm82.a2.v1.exon.2;Parent=Glyma.04G092600.1.Wm82.a2.v1;pacid=30487935 |
| Chr04 | phytozomev10 | CDS | 8158909 | 8159031 | . | + | 0 | ID=Glyma.04G092600.1.Wm82.a2.v1.CDS.2;Parent=Glyma.04G092600.1.Wm82.a2.v1;pacid=30487935 |
| Chr04 | phytozomev10 | exon | 8160067 | 8160183 | . | + | . | ID=Glyma.04G092600.1.Wm82.a2.v1.exon.3;Parent=Glyma.04G092600.1.Wm82.a2.v1;pacid=30487935 |
| Chr04 | phytozomev10 | CDS | 8160067 | 8160183 | . | + | 0 | ID=Glyma.04G092600.1.Wm82.a2.v1.CDS.3;Parent=Glyma.04G092600.1.Wm82.a2.v1;pacid=30487935 |
| Chr04 | phytozomev10 | exon | 8160292 | 8160711 | . | + | . | ID=Glyma.04G092600.1.Wm82.a2.v1.exon.4;Parent=Glyma.04G092600.1.Wm82.a2.v1;pacid=30487935 |
| Chr04 | phytozomev10 | CDS | 8160292 | 8160312 | . | + | 0 | ID=Glyma.04G092600.1.Wm82.a2.v1.CDS.4;Parent=Glyma.04G092600.1.Wm82.a2.v1;pacid=30487935 |
| Chr04 | phytozomev10 | 3p_UTR | 8160313 | 8160711 | . | + | . | ID=Glyma.04G092600.1.Wm82.a2.v1.3p_UTR.1;Parent=Glyma.04G092600.1.Wm82.a2.v1;pacid=30487935 |
| Chr04 | phytozomev10 | gene | 9132954 | 9135203 | . | - | . | ID=Glyma.04G099900.Wm82.a2.v1;Name=Glyma.04G099900;ancestorIdentifier=Glyma04g10820.v1.1 |
| Chr04 | phytozomev10 | mRNA | 9132954 | 9135203 | . | - | . | ID=Glyma.04G099900.2.Wm82.a2.v1;Name=Glyma.04G099900.2;pacid=30487957;longest=0;ancestorIdentifer=Glyma04g10820.4.v1.1;Parent=Glyma.04G099900.Wm82.a2.v1 |
| Chr04 | phytozomev10 | exon | 9134135 | 9135203 | . | - | . | ID=Glyma.04G099900.2.Wm82.a2.v1.exon.1;Parent=Glyma.04G099900.2.Wm82.a2.v1;pacid=30487957 |
| Chr04 | phytozomev10 | 3p_UTR | 9134135 | 9134579 | . | - | . | ID=Glyma.04G099900.2.Wm82.a2.v1.3p_UTR.1;Parent=Glyma.04G099900.2.Wm82.a2.v1;pacid=30487957 |
| Chr04 | phytozomev10 | CDS | 9134580 | 9135098 | . | - | 0 | ID=Glyma.04G099900.2.Wm82.a2.v1.CDS.1;Parent=Glyma.04G099900.2.Wm82.a2.v1;pacid=30487957 |
| Chr04 | phytozomev10 | 5p_UTR | 9135099 | 9135203 | . | - | . | ID=Glyma.04G099900.2.Wm82.a2.v1.5p_UTR.1;Parent=Glyma.04G099900.2.Wm82.a2.v1;pacid=30487957 |
| Chr04 | phytozomev10 | exon | 9133452 | 9133568 | . | - | . | ID=Glyma.04G099900.2.Wm82.a2.v1.exon.2;Parent=Glyma.04G099900.2.Wm82.a2.v1;pacid=30487957 |
| Chr04 | phytozomev10 | 3p_UTR | 9133452 | 9133568 | . | - | . | ID=Glyma.04G099900.2.Wm82.a2.v1.3p_UTR.2;Parent=Glyma.04G099900.2.Wm82.a2.v1;pacid=30487957 |
| Chr04 | phytozomev10 | exon | 9132954 | 9133364 | . | - | . | ID=Glyma.04G099900.2.Wm82.a2.v1.exon.3;Parent=Glyma.04G099900.2.Wm82.a2.v1;pacid=30487957 |
| Chr04 | phytozomev10 | 3p_UTR | 9132954 | 9133364 | . | - | . | ID=Glyma.04G099900.2.Wm82.a2.v1.3p_UTR.3;Parent=Glyma.04G099900.2.Wm82.a2.v1;pacid=30487957 |
| Chr04 | phytozomev10 | mRNA | 9133667 | 9135203 | . | - | . | ID=Glyma.04G099900.3.Wm82.a2.v1;Name=Glyma.04G099900.3;pacid=30487956;longest=0;ancestorIdentifer=Glyma04g10820.3.v1.1;Parent=Glyma.04G099900.Wm82.a2.v1 |
| Chr04 | phytozomev10 | exon | 9134613 | 9135203 | . | - | . | ID=Glyma.04G099900.3.Wm82.a2.v1.exon.1;Parent=Glyma.04G099900.3.Wm82.a2.v1;pacid=30487956 |
| Chr04 | phytozomev10 | CDS | 9134613 | 9135098 | . | - | 0 | ID=Glyma.04G099900.3.Wm82.a2.v1.CDS.1;Parent=Glyma.04G099900.3.Wm82.a2.v1;pacid=30487956 |
| Chr04 | phytozomev10 | 5p_UTR | 9135099 | 9135203 | . | - | . | ID=Glyma.04G099900.3.Wm82.a2.v1.5p_UTR.1;Parent=Glyma.04G099900.3.Wm82.a2.v1;pacid=30487956 |
| Chr04 | phytozomev10 | exon | 9133667 | 9134257 | . | - | . | ID=Glyma.04G099900.3.Wm82.a2.v1.exon.2;Parent=Glyma.04G099900.3.Wm82.a2.v1;pacid=30487956 |
| Chr04 | phytozomev10 | 3p_UTR | 9133667 | 9134038 | . | - | . | ID=Glyma.04G099900.3.Wm82.a2.v1.3p_UTR.1;Parent=Glyma.04G099900.3.Wm82.a2.v1;pacid=30487956 |
| Chr04 | phytozomev10 | CDS | 9134039 | 9134257 | . | - | 0 | ID=Glyma.04G099900.3.Wm82.a2.v1.CDS.2;Parent=Glyma.04G099900.3.Wm82.a2.v1;pacid=30487956 |
| Chr04 | phytozomev10 | mRNA | 9132954 | 9135203 | . | - | . | ID=Glyma.04G099900.1.Wm82.a2.v1;Name=Glyma.04G099900.1;pacid=30487955;longest=1;ancestorIdentifer=Glyma04g10820.1.v1.1;Parent=Glyma.04G099900.Wm82.a2.v1 |
| Chr04 | phytozomev10 | exon | 9134613 | 9135203 | . | - | . | ID=Glyma.04G099900.1.Wm82.a2.v1.exon.1;Parent=Glyma.04G099900.1.Wm82.a2.v1;pacid=30487955 |
| Chr04 | phytozomev10 | CDS | 9134613 | 9135098 | . | - | 0 | ID=Glyma.04G099900.1.Wm82.a2.v1.CDS.1;Parent=Glyma.04G099900.1.Wm82.a2.v1;pacid=30487955 |
| Chr04 | phytozomev10 | 5p_UTR | 9135099 | 9135203 | . | - | . | ID=Glyma.04G099900.1.Wm82.a2.v1.5p_UTR.1;Parent=Glyma.04G099900.1.Wm82.a2.v1;pacid=30487955 |
| Chr04 | phytozomev10 | exon | 9134135 | 9134257 | . | - | . | ID=Glyma.04G099900.1.Wm82.a2.v1.exon.2;Parent=Glyma.04G099900.1.Wm82.a2.v1;pacid=30487955 |
| Chr04 | phytozomev10 | CDS | 9134135 | 9134257 | . | - | 0 | ID=Glyma.04G099900.1.Wm82.a2.v1.CDS.2;Parent=Glyma.04G099900.1.Wm82.a2.v1;pacid=30487955 |
| Chr04 | phytozomev10 | exon | 9132954 | 9133568 | . | - | . | ID=Glyma.04G099900.1.Wm82.a2.v1.exon.3;Parent=Glyma.04G099900.1.Wm82.a2.v1;pacid=30487955 |
| Chr04 | phytozomev10 | 3p_UTR | 9132954 | 9133310 | . | - | . | ID=Glyma.04G099900.1.Wm82.a2.v1.3p_UTR.1;Parent=Glyma.04G099900.1.Wm82.a2.v1;pacid=30487955 |
| Chr04 | phytozomev10 | CDS | 9133311 | 9133568 | . | - | 0 | ID=Glyma.04G099900.1.Wm82.a2.v1.CDS.3;Parent=Glyma.04G099900.1.Wm82.a2.v1;pacid=30487955 |
| Chr04 | phytozomev10 | gene | 45129363 | 45133820 | . | - | . | ID=Glyma.04G183400.Wm82.a2.v1;Name=Glyma.04G183400;ancestorIdentifier=Glyma04g35600.v1.1 |
| Chr04 | phytozomev10 | mRNA | 45129363 | 45133820 | . | - | . | ID=Glyma.04G183400.1.Wm82.a2.v1;Name=Glyma.04G183400.1;pacid=30488248;longest=1;ancestorIdentifer=Glyma04g35600.1.v1.1;Parent=Glyma.04G183400.Wm82.a2.v1 |
| Chr04 | phytozomev10 | exon | 45133312 | 45133820 | . | - | . | ID=Glyma.04G183400.1.Wm82.a2.v1.exon.1;Parent=Glyma.04G183400.1.Wm82.a2.v1;pacid=30488248 |
| Chr04 | phytozomev10 | 5p_UTR | 45133312 | 45133820 | . | - | . | ID=Glyma.04G183400.1.Wm82.a2.v1.5p_UTR.1;Parent=Glyma.04G183400.1.Wm82.a2.v1;pacid=30488248 |
| Chr04 | phytozomev10 | exon | 45132339 | 45132770 | . | - | . | ID=Glyma.04G183400.1.Wm82.a2.v1.exon.2;Parent=Glyma.04G183400.1.Wm82.a2.v1;pacid=30488248 |
| Chr04 | phytozomev10 | CDS | 45132339 | 45132758 | . | - | 0 | ID=Glyma.04G183400.1.Wm82.a2.v1.CDS.1;Parent=Glyma.04G183400.1.Wm82.a2.v1;pacid=30488248 |
| Chr04 | phytozomev10 | 5p_UTR | 45132759 | 45132770 | . | - | . | ID=Glyma.04G183400.1.Wm82.a2.v1.5p_UTR.2;Parent=Glyma.04G183400.1.Wm82.a2.v1;pacid=30488248 |
| Chr04 | phytozomev10 | exon | 45130706 | 45132255 | . | - | . | ID=Glyma.04G183400.1.Wm82.a2.v1.exon.3;Parent=Glyma.04G183400.1.Wm82.a2.v1;pacid=30488248 |
| Chr04 | phytozomev10 | CDS | 45130706 | 45132255 | . | - | 0 | ID=Glyma.04G183400.1.Wm82.a2.v1.CDS.2;Parent=Glyma.04G183400.1.Wm82.a2.v1;pacid=30488248 |
| Chr04 | phytozomev10 | exon | 45129363 | 45129957 | . | - | . | ID=Glyma.04G183400.1.Wm82.a2.v1.exon.4;Parent=Glyma.04G183400.1.Wm82.a2.v1;pacid=30488248 |
| Chr04 | phytozomev10 | 3p_UTR | 45129363 | 45129746 | . | - | . | ID=Glyma.04G183400.1.Wm82.a2.v1.3p_UTR.1;Parent=Glyma.04G183400.1.Wm82.a2.v1;pacid=30488248 |
| Chr04 | phytozomev10 | CDS | 45129747 | 45129957 | . | - | 1 | ID=Glyma.04G183400.1.Wm82.a2.v1.CDS.3;Parent=Glyma.04G183400.1.Wm82.a2.v1;pacid=30488248 |
| Chr05 | phytozomev10 | gene | 35025422 | 35029392 | . | + | . | ID=Glyma.05G158100.Wm82.a2.v1;Name=Glyma.05G158100;ancestorIdentifier=Glyma05g29080.v1.1 |
| Chr05 | phytozomev10 | mRNA | 35025422 | 35029381 | . | + | . | ID=Glyma.05G158100.3.Wm82.a2.v1;Name=Glyma.05G158100.3;pacid=30525611;longest=0;Parent=Glyma.05G158100.Wm82.a2.v1 |
| Chr05 | phytozomev10 | exon | 35025422 | 35025762 | . | + | . | ID=Glyma.05G158100.3.Wm82.a2.v1.exon.1;Parent=Glyma.05G158100.3.Wm82.a2.v1;pacid=30525611 |
| Chr05 | phytozomev10 | 5p_UTR | 35025422 | 35025689 | . | + | . | ID=Glyma.05G158100.3.Wm82.a2.v1.5p_UTR.1;Parent=Glyma.05G158100.3.Wm82.a2.v1;pacid=30525611 |
| Chr05 | phytozomev10 | CDS | 35025690 | 35025762 | . | + | 0 | ID=Glyma.05G158100.3.Wm82.a2.v1.CDS.1;Parent=Glyma.05G158100.3.Wm82.a2.v1;pacid=30525611 |
| Chr05 | phytozomev10 | exon | 35025964 | 35026270 | . | + | . | ID=Glyma.05G158100.3.Wm82.a2.v1.exon.2;Parent=Glyma.05G158100.3.Wm82.a2.v1;pacid=30525611 |
| Chr05 | phytozomev10 | CDS | 35025964 | 35026270 | . | + | 2 | ID=Glyma.05G158100.3.Wm82.a2.v1.CDS.2;Parent=Glyma.05G158100.3.Wm82.a2.v1;pacid=30525611 |
| Chr05 | phytozomev10 | exon | 35026354 | 35026441 | . | + | . | ID=Glyma.05G158100.3.Wm82.a2.v1.exon.3;Parent=Glyma.05G158100.3.Wm82.a2.v1;pacid=30525611 |
| Chr05 | phytozomev10 | CDS | 35026354 | 35026441 | . | + | 1 | ID=Glyma.05G158100.3.Wm82.a2.v1.CDS.3;Parent=Glyma.05G158100.3.Wm82.a2.v1;pacid=30525611 |
| Chr05 | phytozomev10 | exon | 35027152 | 35027261 | . | + | . | ID=Glyma.05G158100.3.Wm82.a2.v1.exon.4;Parent=Glyma.05G158100.3.Wm82.a2.v1;pacid=30525611 |
| Chr05 | phytozomev10 | CDS | 35027152 | 35027261 | . | + | 0 | ID=Glyma.05G158100.3.Wm82.a2.v1.CDS.4;Parent=Glyma.05G158100.3.Wm82.a2.v1;pacid=30525611 |
| Chr05 | phytozomev10 | exon | 35027382 | 35027530 | . | + | . | ID=Glyma.05G158100.3.Wm82.a2.v1.exon.5;Parent=Glyma.05G158100.3.Wm82.a2.v1;pacid=30525611 |
| Chr05 | phytozomev10 | CDS | 35027382 | 35027530 | . | + | 1 | ID=Glyma.05G158100.3.Wm82.a2.v1.CDS.5;Parent=Glyma.05G158100.3.Wm82.a2.v1;pacid=30525611 |
| Chr05 | phytozomev10 | exon | 35028894 | 35028943 | . | + | . | ID=Glyma.05G158100.3.Wm82.a2.v1.exon.6;Parent=Glyma.05G158100.3.Wm82.a2.v1;pacid=30525611 |
| Chr05 | phytozomev10 | CDS | 35028894 | 35028943 | . | + | 2 | ID=Glyma.05G158100.3.Wm82.a2.v1.CDS.6;Parent=Glyma.05G158100.3.Wm82.a2.v1;pacid=30525611 |
| Chr05 | phytozomev10 | exon | 35029023 | 35029381 | . | + | . | ID=Glyma.05G158100.3.Wm82.a2.v1.exon.7;Parent=Glyma.05G158100.3.Wm82.a2.v1;pacid=30525611 |
| Chr05 | phytozomev10 | 3p_UTR | 35029023 | 35029381 | . | + | . | ID=Glyma.05G158100.3.Wm82.a2.v1.3p_UTR.1;Parent=Glyma.05G158100.3.Wm82.a2.v1;pacid=30525611 |
| Chr05 | phytozomev10 | mRNA | 35025422 | 35029381 | . | + | . | ID=Glyma.05G158100.4.Wm82.a2.v1;Name=Glyma.05G158100.4;pacid=30525612;longest=0;ancestorIdentifer=Glyma05g29080.11.v1.1;Parent=Glyma.05G158100.Wm82.a2.v1 |
| Chr05 | phytozomev10 | exon | 35025422 | 35025762 | . | + | . | ID=Glyma.05G158100.4.Wm82.a2.v1.exon.1;Parent=Glyma.05G158100.4.Wm82.a2.v1;pacid=30525612 |
| Chr05 | phytozomev10 | 5p_UTR | 35025422 | 35025689 | . | + | . | ID=Glyma.05G158100.4.Wm82.a2.v1.5p_UTR.1;Parent=Glyma.05G158100.4.Wm82.a2.v1;pacid=30525612 |
| Chr05 | phytozomev10 | CDS | 35025690 | 35025762 | . | + | 0 | ID=Glyma.05G158100.4.Wm82.a2.v1.CDS.1;Parent=Glyma.05G158100.4.Wm82.a2.v1;pacid=30525612 |
| Chr05 | phytozomev10 | exon | 35025964 | 35026270 | . | + | . | ID=Glyma.05G158100.4.Wm82.a2.v1.exon.2;Parent=Glyma.05G158100.4.Wm82.a2.v1;pacid=30525612 |
| Chr05 | phytozomev10 | CDS | 35025964 | 35026270 | . | + | 2 | ID=Glyma.05G158100.4.Wm82.a2.v1.CDS.2;Parent=Glyma.05G158100.4.Wm82.a2.v1;pacid=30525612 |
| Chr05 | phytozomev10 | exon | 35026354 | 35026441 | . | + | . | ID=Glyma.05G158100.4.Wm82.a2.v1.exon.3;Parent=Glyma.05G158100.4.Wm82.a2.v1;pacid=30525612 |
| Chr05 | phytozomev10 | CDS | 35026354 | 35026441 | . | + | 1 | ID=Glyma.05G158100.4.Wm82.a2.v1.CDS.3;Parent=Glyma.05G158100.4.Wm82.a2.v1;pacid=30525612 |
| Chr05 | phytozomev10 | exon | 35027152 | 35027261 | . | + | . | ID=Glyma.05G158100.4.Wm82.a2.v1.exon.4;Parent=Glyma.05G158100.4.Wm82.a2.v1;pacid=30525612 |
| Chr05 | phytozomev10 | CDS | 35027152 | 35027261 | . | + | 0 | ID=Glyma.05G158100.4.Wm82.a2.v1.CDS.4;Parent=Glyma.05G158100.4.Wm82.a2.v1;pacid=30525612 |
| Chr05 | phytozomev10 | exon | 35027382 | 35027530 | . | + | . | ID=Glyma.05G158100.4.Wm82.a2.v1.exon.5;Parent=Glyma.05G158100.4.Wm82.a2.v1;pacid=30525612 |
| Chr05 | phytozomev10 | CDS | 35027382 | 35027530 | . | + | 1 | ID=Glyma.05G158100.4.Wm82.a2.v1.CDS.5;Parent=Glyma.05G158100.4.Wm82.a2.v1;pacid=30525612 |
| Chr05 | phytozomev10 | exon | 35028894 | 35028943 | . | + | . | ID=Glyma.05G158100.4.Wm82.a2.v1.exon.6;Parent=Glyma.05G158100.4.Wm82.a2.v1;pacid=30525612 |
| Chr05 | phytozomev10 | CDS | 35028894 | 35028943 | . | + | 2 | ID=Glyma.05G158100.4.Wm82.a2.v1.CDS.6;Parent=Glyma.05G158100.4.Wm82.a2.v1;pacid=30525612 |
| Chr05 | phytozomev10 | exon | 35029018 | 35029381 | . | + | . | ID=Glyma.05G158100.4.Wm82.a2.v1.exon.7;Parent=Glyma.05G158100.4.Wm82.a2.v1;pacid=30525612 |
| Chr05 | phytozomev10 | 3p_UTR | 35029018 | 35029381 | . | + | . | ID=Glyma.05G158100.4.Wm82.a2.v1.3p_UTR.1;Parent=Glyma.05G158100.4.Wm82.a2.v1;pacid=30525612 |
| Chr05 | phytozomev10 | mRNA | 35025422 | 35029392 | . | + | . | ID=Glyma.05G158100.2.Wm82.a2.v1;Name=Glyma.05G158100.2;pacid=30525609;longest=0;ancestorIdentifer=Glyma05g29080.14.v1.1;Parent=Glyma.05G158100.Wm82.a2.v1 |
| Chr05 | phytozomev10 | exon | 35025422 | 35025762 | . | + | . | ID=Glyma.05G158100.2.Wm82.a2.v1.exon.1;Parent=Glyma.05G158100.2.Wm82.a2.v1;pacid=30525609 |
| Chr05 | phytozomev10 | 5p_UTR | 35025422 | 35025689 | . | + | . | ID=Glyma.05G158100.2.Wm82.a2.v1.5p_UTR.1;Parent=Glyma.05G158100.2.Wm82.a2.v1;pacid=30525609 |
| Chr05 | phytozomev10 | CDS | 35025690 | 35025762 | . | + | 0 | ID=Glyma.05G158100.2.Wm82.a2.v1.CDS.1;Parent=Glyma.05G158100.2.Wm82.a2.v1;pacid=30525609 |
| Chr05 | phytozomev10 | exon | 35025964 | 35026270 | . | + | . | ID=Glyma.05G158100.2.Wm82.a2.v1.exon.2;Parent=Glyma.05G158100.2.Wm82.a2.v1;pacid=30525609 |
| Chr05 | phytozomev10 | CDS | 35025964 | 35026270 | . | + | 2 | ID=Glyma.05G158100.2.Wm82.a2.v1.CDS.2;Parent=Glyma.05G158100.2.Wm82.a2.v1;pacid=30525609 |
| Chr05 | phytozomev10 | exon | 35026354 | 35026441 | . | + | . | ID=Glyma.05G158100.2.Wm82.a2.v1.exon.3;Parent=Glyma.05G158100.2.Wm82.a2.v1;pacid=30525609 |
| Chr05 | phytozomev10 | CDS | 35026354 | 35026441 | . | + | 1 | ID=Glyma.05G158100.2.Wm82.a2.v1.CDS.3;Parent=Glyma.05G158100.2.Wm82.a2.v1;pacid=30525609 |
| Chr05 | phytozomev10 | exon | 35027152 | 35027261 | . | + | . | ID=Glyma.05G158100.2.Wm82.a2.v1.exon.4;Parent=Glyma.05G158100.2.Wm82.a2.v1;pacid=30525609 |
| Chr05 | phytozomev10 | CDS | 35027152 | 35027261 | . | + | 0 | ID=Glyma.05G158100.2.Wm82.a2.v1.CDS.4;Parent=Glyma.05G158100.2.Wm82.a2.v1;pacid=30525609 |
| Chr05 | phytozomev10 | exon | 35027382 | 35027530 | . | + | . | ID=Glyma.05G158100.2.Wm82.a2.v1.exon.5;Parent=Glyma.05G158100.2.Wm82.a2.v1;pacid=30525609 |
| Chr05 | phytozomev10 | CDS | 35027382 | 35027530 | . | + | 1 | ID=Glyma.05G158100.2.Wm82.a2.v1.CDS.5;Parent=Glyma.05G158100.2.Wm82.a2.v1;pacid=30525609 |
| Chr05 | phytozomev10 | exon | 35028894 | 35029392 | . | + | . | ID=Glyma.05G158100.2.Wm82.a2.v1.exon.6;Parent=Glyma.05G158100.2.Wm82.a2.v1;pacid=30525609 |
| Chr05 | phytozomev10 | CDS | 35028894 | 35028943 | . | + | 2 | ID=Glyma.05G158100.2.Wm82.a2.v1.CDS.6;Parent=Glyma.05G158100.2.Wm82.a2.v1;pacid=30525609 |
| Chr05 | phytozomev10 | 3p_UTR | 35028944 | 35029392 | . | + | . | ID=Glyma.05G158100.2.Wm82.a2.v1.3p_UTR.1;Parent=Glyma.05G158100.2.Wm82.a2.v1;pacid=30525609 |
| Chr05 | phytozomev10 | mRNA | 35025422 | 35029381 | . | + | . | ID=Glyma.05G158100.5.Wm82.a2.v1;Name=Glyma.05G158100.5;pacid=30525610;longest=0;Parent=Glyma.05G158100.Wm82.a2.v1 |
| Chr05 | phytozomev10 | exon | 35025422 | 35025762 | . | + | . | ID=Glyma.05G158100.5.Wm82.a2.v1.exon.1;Parent=Glyma.05G158100.5.Wm82.a2.v1;pacid=30525610 |
| Chr05 | phytozomev10 | 5p_UTR | 35025422 | 35025689 | . | + | . | ID=Glyma.05G158100.5.Wm82.a2.v1.5p_UTR.1;Parent=Glyma.05G158100.5.Wm82.a2.v1;pacid=30525610 |
| Chr05 | phytozomev10 | CDS | 35025690 | 35025762 | . | + | 0 | ID=Glyma.05G158100.5.Wm82.a2.v1.CDS.1;Parent=Glyma.05G158100.5.Wm82.a2.v1;pacid=30525610 |
| Chr05 | phytozomev10 | exon | 35025964 | 35026270 | . | + | . | ID=Glyma.05G158100.5.Wm82.a2.v1.exon.2;Parent=Glyma.05G158100.5.Wm82.a2.v1;pacid=30525610 |
| Chr05 | phytozomev10 | CDS | 35025964 | 35026270 | . | + | 2 | ID=Glyma.05G158100.5.Wm82.a2.v1.CDS.2;Parent=Glyma.05G158100.5.Wm82.a2.v1;pacid=30525610 |
| Chr05 | phytozomev10 | exon | 35026354 | 35026441 | . | + | . | ID=Glyma.05G158100.5.Wm82.a2.v1.exon.3;Parent=Glyma.05G158100.5.Wm82.a2.v1;pacid=30525610 |
| Chr05 | phytozomev10 | CDS | 35026354 | 35026441 | . | + | 1 | ID=Glyma.05G158100.5.Wm82.a2.v1.CDS.3;Parent=Glyma.05G158100.5.Wm82.a2.v1;pacid=30525610 |
| Chr05 | phytozomev10 | exon | 35027152 | 35027261 | . | + | . | ID=Glyma.05G158100.5.Wm82.a2.v1.exon.4;Parent=Glyma.05G158100.5.Wm82.a2.v1;pacid=30525610 |
| Chr05 | phytozomev10 | CDS | 35027152 | 35027261 | . | + | 0 | ID=Glyma.05G158100.5.Wm82.a2.v1.CDS.4;Parent=Glyma.05G158100.5.Wm82.a2.v1;pacid=30525610 |
| Chr05 | phytozomev10 | exon | 35027382 | 35027530 | . | + | . | ID=Glyma.05G158100.5.Wm82.a2.v1.exon.5;Parent=Glyma.05G158100.5.Wm82.a2.v1;pacid=30525610 |
| Chr05 | phytozomev10 | CDS | 35027382 | 35027530 | . | + | 1 | ID=Glyma.05G158100.5.Wm82.a2.v1.CDS.5;Parent=Glyma.05G158100.5.Wm82.a2.v1;pacid=30525610 |
| Chr05 | phytozomev10 | exon | 35028894 | 35028943 | . | + | . | ID=Glyma.05G158100.5.Wm82.a2.v1.exon.6;Parent=Glyma.05G158100.5.Wm82.a2.v1;pacid=30525610 |
| Chr05 | phytozomev10 | CDS | 35028894 | 35028943 | . | + | 2 | ID=Glyma.05G158100.5.Wm82.a2.v1.CDS.6;Parent=Glyma.05G158100.5.Wm82.a2.v1;pacid=30525610 |
| Chr05 | phytozomev10 | exon | 35029026 | 35029381 | . | + | . | ID=Glyma.05G158100.5.Wm82.a2.v1.exon.7;Parent=Glyma.05G158100.5.Wm82.a2.v1;pacid=30525610 |
| Chr05 | phytozomev10 | 3p_UTR | 35029026 | 35029381 | . | + | . | ID=Glyma.05G158100.5.Wm82.a2.v1.3p_UTR.1;Parent=Glyma.05G158100.5.Wm82.a2.v1;pacid=30525610 |
| Chr05 | phytozomev10 | mRNA | 35025422 | 35029381 | . | + | . | ID=Glyma.05G158100.6.Wm82.a2.v1;Name=Glyma.05G158100.6;pacid=30525607;longest=0;Parent=Glyma.05G158100.Wm82.a2.v1 |
| Chr05 | phytozomev10 | exon | 35025422 | 35025762 | . | + | . | ID=Glyma.05G158100.6.Wm82.a2.v1.exon.1;Parent=Glyma.05G158100.6.Wm82.a2.v1;pacid=30525607 |
| Chr05 | phytozomev10 | 5p_UTR | 35025422 | 35025689 | . | + | . | ID=Glyma.05G158100.6.Wm82.a2.v1.5p_UTR.1;Parent=Glyma.05G158100.6.Wm82.a2.v1;pacid=30525607 |
| Chr05 | phytozomev10 | CDS | 35025690 | 35025762 | . | + | 0 | ID=Glyma.05G158100.6.Wm82.a2.v1.CDS.1;Parent=Glyma.05G158100.6.Wm82.a2.v1;pacid=30525607 |
| Chr05 | phytozomev10 | exon | 35025964 | 35026270 | . | + | . | ID=Glyma.05G158100.6.Wm82.a2.v1.exon.2;Parent=Glyma.05G158100.6.Wm82.a2.v1;pacid=30525607 |
| Chr05 | phytozomev10 | CDS | 35025964 | 35026270 | . | + | 2 | ID=Glyma.05G158100.6.Wm82.a2.v1.CDS.2;Parent=Glyma.05G158100.6.Wm82.a2.v1;pacid=30525607 |
| Chr05 | phytozomev10 | exon | 35026354 | 35026441 | . | + | . | ID=Glyma.05G158100.6.Wm82.a2.v1.exon.3;Parent=Glyma.05G158100.6.Wm82.a2.v1;pacid=30525607 |
| Chr05 | phytozomev10 | CDS | 35026354 | 35026441 | . | + | 1 | ID=Glyma.05G158100.6.Wm82.a2.v1.CDS.3;Parent=Glyma.05G158100.6.Wm82.a2.v1;pacid=30525607 |
| Chr05 | phytozomev10 | exon | 35027152 | 35027261 | . | + | . | ID=Glyma.05G158100.6.Wm82.a2.v1.exon.4;Parent=Glyma.05G158100.6.Wm82.a2.v1;pacid=30525607 |
| Chr05 | phytozomev10 | CDS | 35027152 | 35027261 | . | + | 0 | ID=Glyma.05G158100.6.Wm82.a2.v1.CDS.4;Parent=Glyma.05G158100.6.Wm82.a2.v1;pacid=30525607 |
| Chr05 | phytozomev10 | exon | 35027382 | 35027530 | . | + | . | ID=Glyma.05G158100.6.Wm82.a2.v1.exon.5;Parent=Glyma.05G158100.6.Wm82.a2.v1;pacid=30525607 |
| Chr05 | phytozomev10 | CDS | 35027382 | 35027530 | . | + | 1 | ID=Glyma.05G158100.6.Wm82.a2.v1.CDS.5;Parent=Glyma.05G158100.6.Wm82.a2.v1;pacid=30525607 |
| Chr05 | phytozomev10 | exon | 35028891 | 35028943 | . | + | . | ID=Glyma.05G158100.6.Wm82.a2.v1.exon.6;Parent=Glyma.05G158100.6.Wm82.a2.v1;pacid=30525607 |
| Chr05 | phytozomev10 | CDS | 35028891 | 35028943 | . | + | 2 | ID=Glyma.05G158100.6.Wm82.a2.v1.CDS.6;Parent=Glyma.05G158100.6.Wm82.a2.v1;pacid=30525607 |
| Chr05 | phytozomev10 | exon | 35029018 | 35029381 | . | + | . | ID=Glyma.05G158100.6.Wm82.a2.v1.exon.7;Parent=Glyma.05G158100.6.Wm82.a2.v1;pacid=30525607 |
| Chr05 | phytozomev10 | 3p_UTR | 35029018 | 35029381 | . | + | . | ID=Glyma.05G158100.6.Wm82.a2.v1.3p_UTR.1;Parent=Glyma.05G158100.6.Wm82.a2.v1;pacid=30525607 |
| Chr05 | phytozomev10 | mRNA | 35025422 | 35029381 | . | + | . | ID=Glyma.05G158100.7.Wm82.a2.v1;Name=Glyma.05G158100.7;pacid=30525608;longest=0;Parent=Glyma.05G158100.Wm82.a2.v1 |
| Chr05 | phytozomev10 | exon | 35025422 | 35025762 | . | + | . | ID=Glyma.05G158100.7.Wm82.a2.v1.exon.1;Parent=Glyma.05G158100.7.Wm82.a2.v1;pacid=30525608 |
| Chr05 | phytozomev10 | 5p_UTR | 35025422 | 35025689 | . | + | . | ID=Glyma.05G158100.7.Wm82.a2.v1.5p_UTR.1;Parent=Glyma.05G158100.7.Wm82.a2.v1;pacid=30525608 |
| Chr05 | phytozomev10 | CDS | 35025690 | 35025762 | . | + | 0 | ID=Glyma.05G158100.7.Wm82.a2.v1.CDS.1;Parent=Glyma.05G158100.7.Wm82.a2.v1;pacid=30525608 |
| Chr05 | phytozomev10 | exon | 35025964 | 35026270 | . | + | . | ID=Glyma.05G158100.7.Wm82.a2.v1.exon.2;Parent=Glyma.05G158100.7.Wm82.a2.v1;pacid=30525608 |
| Chr05 | phytozomev10 | CDS | 35025964 | 35026270 | . | + | 2 | ID=Glyma.05G158100.7.Wm82.a2.v1.CDS.2;Parent=Glyma.05G158100.7.Wm82.a2.v1;pacid=30525608 |
| Chr05 | phytozomev10 | exon | 35026354 | 35026441 | . | + | . | ID=Glyma.05G158100.7.Wm82.a2.v1.exon.3;Parent=Glyma.05G158100.7.Wm82.a2.v1;pacid=30525608 |
| Chr05 | phytozomev10 | CDS | 35026354 | 35026441 | . | + | 1 | ID=Glyma.05G158100.7.Wm82.a2.v1.CDS.3;Parent=Glyma.05G158100.7.Wm82.a2.v1;pacid=30525608 |
| Chr05 | phytozomev10 | exon | 35027152 | 35027261 | . | + | . | ID=Glyma.05G158100.7.Wm82.a2.v1.exon.4;Parent=Glyma.05G158100.7.Wm82.a2.v1;pacid=30525608 |
| Chr05 | phytozomev10 | CDS | 35027152 | 35027261 | . | + | 0 | ID=Glyma.05G158100.7.Wm82.a2.v1.CDS.4;Parent=Glyma.05G158100.7.Wm82.a2.v1;pacid=30525608 |
| Chr05 | phytozomev10 | exon | 35027382 | 35027530 | . | + | . | ID=Glyma.05G158100.7.Wm82.a2.v1.exon.5;Parent=Glyma.05G158100.7.Wm82.a2.v1;pacid=30525608 |
| Chr05 | phytozomev10 | CDS | 35027382 | 35027530 | . | + | 1 | ID=Glyma.05G158100.7.Wm82.a2.v1.CDS.5;Parent=Glyma.05G158100.7.Wm82.a2.v1;pacid=30525608 |
| Chr05 | phytozomev10 | exon | 35028891 | 35028943 | . | + | . | ID=Glyma.05G158100.7.Wm82.a2.v1.exon.6;Parent=Glyma.05G158100.7.Wm82.a2.v1;pacid=30525608 |
| Chr05 | phytozomev10 | CDS | 35028891 | 35028943 | . | + | 2 | ID=Glyma.05G158100.7.Wm82.a2.v1.CDS.6;Parent=Glyma.05G158100.7.Wm82.a2.v1;pacid=30525608 |
| Chr05 | phytozomev10 | exon | 35029023 | 35029381 | . | + | . | ID=Glyma.05G158100.7.Wm82.a2.v1.exon.7;Parent=Glyma.05G158100.7.Wm82.a2.v1;pacid=30525608 |
| Chr05 | phytozomev10 | 3p_UTR | 35029023 | 35029381 | . | + | . | ID=Glyma.05G158100.7.Wm82.a2.v1.3p_UTR.1;Parent=Glyma.05G158100.7.Wm82.a2.v1;pacid=30525608 |
| Chr05 | phytozomev10 | mRNA | 35025422 | 35029392 | . | + | . | ID=Glyma.05G158100.1.Wm82.a2.v1;Name=Glyma.05G158100.1;pacid=30525605;longest=1;ancestorIdentifer=Glyma05g29080.9.v1.1;Parent=Glyma.05G158100.Wm82.a2.v1 |
| Chr05 | phytozomev10 | exon | 35025422 | 35025762 | . | + | . | ID=Glyma.05G158100.1.Wm82.a2.v1.exon.1;Parent=Glyma.05G158100.1.Wm82.a2.v1;pacid=30525605 |
| Chr05 | phytozomev10 | 5p_UTR | 35025422 | 35025689 | . | + | . | ID=Glyma.05G158100.1.Wm82.a2.v1.5p_UTR.1;Parent=Glyma.05G158100.1.Wm82.a2.v1;pacid=30525605 |
| Chr05 | phytozomev10 | CDS | 35025690 | 35025762 | . | + | 0 | ID=Glyma.05G158100.1.Wm82.a2.v1.CDS.1;Parent=Glyma.05G158100.1.Wm82.a2.v1;pacid=30525605 |
| Chr05 | phytozomev10 | exon | 35025964 | 35026270 | . | + | . | ID=Glyma.05G158100.1.Wm82.a2.v1.exon.2;Parent=Glyma.05G158100.1.Wm82.a2.v1;pacid=30525605 |
| Chr05 | phytozomev10 | CDS | 35025964 | 35026270 | . | + | 2 | ID=Glyma.05G158100.1.Wm82.a2.v1.CDS.2;Parent=Glyma.05G158100.1.Wm82.a2.v1;pacid=30525605 |
| Chr05 | phytozomev10 | exon | 35026354 | 35026441 | . | + | . | ID=Glyma.05G158100.1.Wm82.a2.v1.exon.3;Parent=Glyma.05G158100.1.Wm82.a2.v1;pacid=30525605 |
| Chr05 | phytozomev10 | CDS | 35026354 | 35026441 | . | + | 1 | ID=Glyma.05G158100.1.Wm82.a2.v1.CDS.3;Parent=Glyma.05G158100.1.Wm82.a2.v1;pacid=30525605 |
| Chr05 | phytozomev10 | exon | 35027152 | 35027261 | . | + | . | ID=Glyma.05G158100.1.Wm82.a2.v1.exon.4;Parent=Glyma.05G158100.1.Wm82.a2.v1;pacid=30525605 |
| Chr05 | phytozomev10 | CDS | 35027152 | 35027261 | . | + | 0 | ID=Glyma.05G158100.1.Wm82.a2.v1.CDS.4;Parent=Glyma.05G158100.1.Wm82.a2.v1;pacid=30525605 |
| Chr05 | phytozomev10 | exon | 35027382 | 35027530 | . | + | . | ID=Glyma.05G158100.1.Wm82.a2.v1.exon.5;Parent=Glyma.05G158100.1.Wm82.a2.v1;pacid=30525605 |
| Chr05 | phytozomev10 | CDS | 35027382 | 35027530 | . | + | 1 | ID=Glyma.05G158100.1.Wm82.a2.v1.CDS.5;Parent=Glyma.05G158100.1.Wm82.a2.v1;pacid=30525605 |
| Chr05 | phytozomev10 | exon | 35028891 | 35029392 | . | + | . | ID=Glyma.05G158100.1.Wm82.a2.v1.exon.6;Parent=Glyma.05G158100.1.Wm82.a2.v1;pacid=30525605 |
| Chr05 | phytozomev10 | CDS | 35028891 | 35028943 | . | + | 2 | ID=Glyma.05G158100.1.Wm82.a2.v1.CDS.6;Parent=Glyma.05G158100.1.Wm82.a2.v1;pacid=30525605 |
| Chr05 | phytozomev10 | 3p_UTR | 35028944 | 35029392 | . | + | . | ID=Glyma.05G158100.1.Wm82.a2.v1.3p_UTR.1;Parent=Glyma.05G158100.1.Wm82.a2.v1;pacid=30525605 |
| Chr05 | phytozomev10 | mRNA | 35025422 | 35029381 | . | + | . | ID=Glyma.05G158100.8.Wm82.a2.v1;Name=Glyma.05G158100.8;pacid=30525606;longest=0;Parent=Glyma.05G158100.Wm82.a2.v1 |
| Chr05 | phytozomev10 | exon | 35025422 | 35025762 | . | + | . | ID=Glyma.05G158100.8.Wm82.a2.v1.exon.1;Parent=Glyma.05G158100.8.Wm82.a2.v1;pacid=30525606 |
| Chr05 | phytozomev10 | 5p_UTR | 35025422 | 35025689 | . | + | . | ID=Glyma.05G158100.8.Wm82.a2.v1.5p_UTR.1;Parent=Glyma.05G158100.8.Wm82.a2.v1;pacid=30525606 |
| Chr05 | phytozomev10 | CDS | 35025690 | 35025762 | . | + | 0 | ID=Glyma.05G158100.8.Wm82.a2.v1.CDS.1;Parent=Glyma.05G158100.8.Wm82.a2.v1;pacid=30525606 |
| Chr05 | phytozomev10 | exon | 35025964 | 35026270 | . | + | . | ID=Glyma.05G158100.8.Wm82.a2.v1.exon.2;Parent=Glyma.05G158100.8.Wm82.a2.v1;pacid=30525606 |
| Chr05 | phytozomev10 | CDS | 35025964 | 35026270 | . | + | 2 | ID=Glyma.05G158100.8.Wm82.a2.v1.CDS.2;Parent=Glyma.05G158100.8.Wm82.a2.v1;pacid=30525606 |
| Chr05 | phytozomev10 | exon | 35026354 | 35026441 | . | + | . | ID=Glyma.05G158100.8.Wm82.a2.v1.exon.3;Parent=Glyma.05G158100.8.Wm82.a2.v1;pacid=30525606 |
| Chr05 | phytozomev10 | CDS | 35026354 | 35026441 | . | + | 1 | ID=Glyma.05G158100.8.Wm82.a2.v1.CDS.3;Parent=Glyma.05G158100.8.Wm82.a2.v1;pacid=30525606 |
| Chr05 | phytozomev10 | exon | 35027152 | 35027261 | . | + | . | ID=Glyma.05G158100.8.Wm82.a2.v1.exon.4;Parent=Glyma.05G158100.8.Wm82.a2.v1;pacid=30525606 |
| Chr05 | phytozomev10 | CDS | 35027152 | 35027261 | . | + | 0 | ID=Glyma.05G158100.8.Wm82.a2.v1.CDS.4;Parent=Glyma.05G158100.8.Wm82.a2.v1;pacid=30525606 |
| Chr05 | phytozomev10 | exon | 35027382 | 35027530 | . | + | . | ID=Glyma.05G158100.8.Wm82.a2.v1.exon.5;Parent=Glyma.05G158100.8.Wm82.a2.v1;pacid=30525606 |
| Chr05 | phytozomev10 | CDS | 35027382 | 35027530 | . | + | 1 | ID=Glyma.05G158100.8.Wm82.a2.v1.CDS.5;Parent=Glyma.05G158100.8.Wm82.a2.v1;pacid=30525606 |
| Chr05 | phytozomev10 | exon | 35028891 | 35028943 | . | + | . | ID=Glyma.05G158100.8.Wm82.a2.v1.exon.6;Parent=Glyma.05G158100.8.Wm82.a2.v1;pacid=30525606 |
| Chr05 | phytozomev10 | CDS | 35028891 | 35028943 | . | + | 2 | ID=Glyma.05G158100.8.Wm82.a2.v1.CDS.6;Parent=Glyma.05G158100.8.Wm82.a2.v1;pacid=30525606 |
| Chr05 | phytozomev10 | exon | 35029026 | 35029381 | . | + | . | ID=Glyma.05G158100.8.Wm82.a2.v1.exon.7;Parent=Glyma.05G158100.8.Wm82.a2.v1;pacid=30525606 |
| Chr05 | phytozomev10 | 3p_UTR | 35029026 | 35029381 | . | + | . | ID=Glyma.05G158100.8.Wm82.a2.v1.3p_UTR.1;Parent=Glyma.05G158100.8.Wm82.a2.v1;pacid=30525606 |
| Chr06 | phytozomev10 | gene | 7432085 | 7434388 | . | + | . | ID=Glyma.06G094400.Wm82.a2.v1;Name=Glyma.06G094400;ancestorIdentifier=Glyma06g09890.v1.1 |
| Chr06 | phytozomev10 | mRNA | 7432085 | 7434388 | . | + | . | ID=Glyma.06G094400.1.Wm82.a2.v1;Name=Glyma.06G094400.1;pacid=30550238;longest=1;Parent=Glyma.06G094400.Wm82.a2.v1 |
| Chr06 | phytozomev10 | exon | 7432085 | 7432644 | . | + | . | ID=Glyma.06G094400.1.Wm82.a2.v1.exon.1;Parent=Glyma.06G094400.1.Wm82.a2.v1;pacid=30550238 |
| Chr06 | phytozomev10 | 5p_UTR | 7432085 | 7432152 | . | + | . | ID=Glyma.06G094400.1.Wm82.a2.v1.5p_UTR.1;Parent=Glyma.06G094400.1.Wm82.a2.v1;pacid=30550238 |
| Chr06 | phytozomev10 | CDS | 7432153 | 7432644 | . | + | 0 | ID=Glyma.06G094400.1.Wm82.a2.v1.CDS.1;Parent=Glyma.06G094400.1.Wm82.a2.v1;pacid=30550238 |
| Chr06 | phytozomev10 | exon | 7432729 | 7432851 | . | + | . | ID=Glyma.06G094400.1.Wm82.a2.v1.exon.2;Parent=Glyma.06G094400.1.Wm82.a2.v1;pacid=30550238 |
| Chr06 | phytozomev10 | CDS | 7432729 | 7432851 | . | + | 0 | ID=Glyma.06G094400.1.Wm82.a2.v1.CDS.2;Parent=Glyma.06G094400.1.Wm82.a2.v1;pacid=30550238 |
| Chr06 | phytozomev10 | exon | 7433893 | 7434009 | . | + | . | ID=Glyma.06G094400.1.Wm82.a2.v1.exon.3;Parent=Glyma.06G094400.1.Wm82.a2.v1;pacid=30550238 |
| Chr06 | phytozomev10 | CDS | 7433893 | 7434009 | . | + | 0 | ID=Glyma.06G094400.1.Wm82.a2.v1.CDS.3;Parent=Glyma.06G094400.1.Wm82.a2.v1;pacid=30550238 |
| Chr06 | phytozomev10 | exon | 7434118 | 7434388 | . | + | . | ID=Glyma.06G094400.1.Wm82.a2.v1.exon.4;Parent=Glyma.06G094400.1.Wm82.a2.v1;pacid=30550238 |
| Chr06 | phytozomev10 | CDS | 7434118 | 7434138 | . | + | 0 | ID=Glyma.06G094400.1.Wm82.a2.v1.CDS.4;Parent=Glyma.06G094400.1.Wm82.a2.v1;pacid=30550238 |
| Chr06 | phytozomev10 | 3p_UTR | 7434139 | 7434388 | . | + | . | ID=Glyma.06G094400.1.Wm82.a2.v1.3p_UTR.1;Parent=Glyma.06G094400.1.Wm82.a2.v1;pacid=30550238 |
| Chr06 | phytozomev10 | gene | 8052625 | 8054939 | . | - | . | ID=Glyma.06G101500.Wm82.a2.v1;Name=Glyma.06G101500;ancestorIdentifier=Glyma06g10650.v1.1 |
| Chr06 | phytozomev10 | mRNA | 8052625 | 8054939 | . | - | . | ID=Glyma.06G101500.3.Wm82.a2.v1;Name=Glyma.06G101500.3;pacid=30550531;longest=0;ancestorIdentifer=Glyma06g10650.4.v1.1;Parent=Glyma.06G101500.Wm82.a2.v1 |
| Chr06 | phytozomev10 | exon | 8053802 | 8054939 | . | - | . | ID=Glyma.06G101500.3.Wm82.a2.v1.exon.1;Parent=Glyma.06G101500.3.Wm82.a2.v1;pacid=30550531 |
| Chr06 | phytozomev10 | 3p_UTR | 8053802 | 8054249 | . | - | . | ID=Glyma.06G101500.3.Wm82.a2.v1.3p_UTR.1;Parent=Glyma.06G101500.3.Wm82.a2.v1;pacid=30550531 |
| Chr06 | phytozomev10 | CDS | 8054250 | 8054780 | . | - | 0 | ID=Glyma.06G101500.3.Wm82.a2.v1.CDS.1;Parent=Glyma.06G101500.3.Wm82.a2.v1;pacid=30550531 |
| Chr06 | phytozomev10 | 5p_UTR | 8054781 | 8054939 | . | - | . | ID=Glyma.06G101500.3.Wm82.a2.v1.5p_UTR.1;Parent=Glyma.06G101500.3.Wm82.a2.v1;pacid=30550531 |
| Chr06 | phytozomev10 | exon | 8053115 | 8053231 | . | - | . | ID=Glyma.06G101500.3.Wm82.a2.v1.exon.2;Parent=Glyma.06G101500.3.Wm82.a2.v1;pacid=30550531 |
| Chr06 | phytozomev10 | 3p_UTR | 8053115 | 8053231 | . | - | . | ID=Glyma.06G101500.3.Wm82.a2.v1.3p_UTR.2;Parent=Glyma.06G101500.3.Wm82.a2.v1;pacid=30550531 |
| Chr06 | phytozomev10 | exon | 8052625 | 8053018 | . | - | . | ID=Glyma.06G101500.3.Wm82.a2.v1.exon.3;Parent=Glyma.06G101500.3.Wm82.a2.v1;pacid=30550531 |
| Chr06 | phytozomev10 | 3p_UTR | 8052625 | 8053018 | . | - | . | ID=Glyma.06G101500.3.Wm82.a2.v1.3p_UTR.3;Parent=Glyma.06G101500.3.Wm82.a2.v1;pacid=30550531 |
| Chr06 | phytozomev10 | mRNA | 8052625 | 8054939 | . | - | . | ID=Glyma.06G101500.2.Wm82.a2.v1;Name=Glyma.06G101500.2;pacid=30550529;longest=0;ancestorIdentifer=Glyma06g10650.2.v1.1;Parent=Glyma.06G101500.Wm82.a2.v1 |
| Chr06 | phytozomev10 | exon | 8054295 | 8054939 | . | - | . | ID=Glyma.06G101500.2.Wm82.a2.v1.exon.1;Parent=Glyma.06G101500.2.Wm82.a2.v1;pacid=30550529 |
| Chr06 | phytozomev10 | CDS | 8054295 | 8054780 | . | - | 0 | ID=Glyma.06G101500.2.Wm82.a2.v1.CDS.1;Parent=Glyma.06G101500.2.Wm82.a2.v1;pacid=30550529 |
| Chr06 | phytozomev10 | 5p_UTR | 8054781 | 8054939 | . | - | . | ID=Glyma.06G101500.2.Wm82.a2.v1.5p_UTR.1;Parent=Glyma.06G101500.2.Wm82.a2.v1;pacid=30550529 |
| Chr06 | phytozomev10 | exon | 8053802 | 8053924 | . | - | . | ID=Glyma.06G101500.2.Wm82.a2.v1.exon.2;Parent=Glyma.06G101500.2.Wm82.a2.v1;pacid=30550529 |
| Chr06 | phytozomev10 | CDS | 8053802 | 8053924 | . | - | 0 | ID=Glyma.06G101500.2.Wm82.a2.v1.CDS.2;Parent=Glyma.06G101500.2.Wm82.a2.v1;pacid=30550529 |
| Chr06 | phytozomev10 | exon | 8053115 | 8053231 | . | - | . | ID=Glyma.06G101500.2.Wm82.a2.v1.exon.3;Parent=Glyma.06G101500.2.Wm82.a2.v1;pacid=30550529 |
| Chr06 | phytozomev10 | CDS | 8053115 | 8053231 | . | - | 0 | ID=Glyma.06G101500.2.Wm82.a2.v1.CDS.3;Parent=Glyma.06G101500.2.Wm82.a2.v1;pacid=30550529 |
| Chr06 | phytozomev10 | exon | 8052625 | 8053018 | . | - | . | ID=Glyma.06G101500.2.Wm82.a2.v1.exon.4;Parent=Glyma.06G101500.2.Wm82.a2.v1;pacid=30550529 |
| Chr06 | phytozomev10 | 3p_UTR | 8052625 | 8052964 | . | - | . | ID=Glyma.06G101500.2.Wm82.a2.v1.3p_UTR.1;Parent=Glyma.06G101500.2.Wm82.a2.v1;pacid=30550529 |
| Chr06 | phytozomev10 | CDS | 8052965 | 8053018 | . | - | 0 | ID=Glyma.06G101500.2.Wm82.a2.v1.CDS.4;Parent=Glyma.06G101500.2.Wm82.a2.v1;pacid=30550529 |
| Chr06 | phytozomev10 | mRNA | 8053279 | 8054939 | . | - | . | ID=Glyma.06G101500.4.Wm82.a2.v1;Name=Glyma.06G101500.4;pacid=30550530;longest=0;ancestorIdentifer=Glyma06g10650.3.v1.1;Parent=Glyma.06G101500.Wm82.a2.v1 |
| Chr06 | phytozomev10 | exon | 8054295 | 8054939 | . | - | . | ID=Glyma.06G101500.4.Wm82.a2.v1.exon.1;Parent=Glyma.06G101500.4.Wm82.a2.v1;pacid=30550530 |
| Chr06 | phytozomev10 | CDS | 8054295 | 8054780 | . | - | 0 | ID=Glyma.06G101500.4.Wm82.a2.v1.CDS.1;Parent=Glyma.06G101500.4.Wm82.a2.v1;pacid=30550530 |
| Chr06 | phytozomev10 | 5p_UTR | 8054781 | 8054939 | . | - | . | ID=Glyma.06G101500.4.Wm82.a2.v1.5p_UTR.1;Parent=Glyma.06G101500.4.Wm82.a2.v1;pacid=30550530 |
| Chr06 | phytozomev10 | exon | 8053279 | 8053924 | . | - | . | ID=Glyma.06G101500.4.Wm82.a2.v1.exon.2;Parent=Glyma.06G101500.4.Wm82.a2.v1;pacid=30550530 |
| Chr06 | phytozomev10 | 3p_UTR | 8053279 | 8053705 | . | - | . | ID=Glyma.06G101500.4.Wm82.a2.v1.3p_UTR.1;Parent=Glyma.06G101500.4.Wm82.a2.v1;pacid=30550530 |
| Chr06 | phytozomev10 | CDS | 8053706 | 8053924 | . | - | 0 | ID=Glyma.06G101500.4.Wm82.a2.v1.CDS.2;Parent=Glyma.06G101500.4.Wm82.a2.v1;pacid=30550530 |
| Chr06 | phytozomev10 | mRNA | 8052625 | 8054939 | . | - | . | ID=Glyma.06G101500.1.Wm82.a2.v1;Name=Glyma.06G101500.1;pacid=30550528;longest=1;ancestorIdentifer=Glyma06g10650.1.v1.1;Parent=Glyma.06G101500.Wm82.a2.v1 |
| Chr06 | phytozomev10 | exon | 8054295 | 8054939 | . | - | . | ID=Glyma.06G101500.1.Wm82.a2.v1.exon.1;Parent=Glyma.06G101500.1.Wm82.a2.v1;pacid=30550528 |
| Chr06 | phytozomev10 | CDS | 8054295 | 8054780 | . | - | 0 | ID=Glyma.06G101500.1.Wm82.a2.v1.CDS.1;Parent=Glyma.06G101500.1.Wm82.a2.v1;pacid=30550528 |
| Chr06 | phytozomev10 | 5p_UTR | 8054781 | 8054939 | . | - | . | ID=Glyma.06G101500.1.Wm82.a2.v1.5p_UTR.1;Parent=Glyma.06G101500.1.Wm82.a2.v1;pacid=30550528 |
| Chr06 | phytozomev10 | exon | 8053802 | 8053924 | . | - | . | ID=Glyma.06G101500.1.Wm82.a2.v1.exon.2;Parent=Glyma.06G101500.1.Wm82.a2.v1;pacid=30550528 |
| Chr06 | phytozomev10 | CDS | 8053802 | 8053924 | . | - | 0 | ID=Glyma.06G101500.1.Wm82.a2.v1.CDS.2;Parent=Glyma.06G101500.1.Wm82.a2.v1;pacid=30550528 |
| Chr06 | phytozomev10 | exon | 8053115 | 8053231 | . | - | . | ID=Glyma.06G101500.1.Wm82.a2.v1.exon.3;Parent=Glyma.06G101500.1.Wm82.a2.v1;pacid=30550528 |
| Chr06 | phytozomev10 | CDS | 8053115 | 8053231 | . | - | 0 | ID=Glyma.06G101500.1.Wm82.a2.v1.CDS.3;Parent=Glyma.06G101500.1.Wm82.a2.v1;pacid=30550528 |
| Chr06 | phytozomev10 | exon | 8052625 | 8053005 | . | - | . | ID=Glyma.06G101500.1.Wm82.a2.v1.exon.4;Parent=Glyma.06G101500.1.Wm82.a2.v1;pacid=30550528 |
| Chr06 | phytozomev10 | 3p_UTR | 8052625 | 8052891 | . | - | . | ID=Glyma.06G101500.1.Wm82.a2.v1.3p_UTR.1;Parent=Glyma.06G101500.1.Wm82.a2.v1;pacid=30550528 |
| Chr06 | phytozomev10 | CDS | 8052892 | 8053005 | . | - | 0 | ID=Glyma.06G101500.1.Wm82.a2.v1.CDS.4;Parent=Glyma.06G101500.1.Wm82.a2.v1;pacid=30550528 |
| Chr06 | phytozomev10 | gene | 15705290 | 15709591 | . | - | . | ID=Glyma.06G182800.Wm82.a2.v1;Name=Glyma.06G182800;ancestorIdentifier=Glyma06g19430.v1.1 |
| Chr06 | phytozomev10 | mRNA | 15705310 | 15709021 | . | - | . | ID=Glyma.06G182800.2.Wm82.a2.v1;Name=Glyma.06G182800.2;pacid=30553595;longest=0;Parent=Glyma.06G182800.Wm82.a2.v1 |
| Chr06 | phytozomev10 | exon | 15708846 | 15709021 | . | - | . | ID=Glyma.06G182800.2.Wm82.a2.v1.exon.1;Parent=Glyma.06G182800.2.Wm82.a2.v1;pacid=30553595 |
| Chr06 | phytozomev10 | 5p_UTR | 15708846 | 15709021 | . | - | . | ID=Glyma.06G182800.2.Wm82.a2.v1.5p_UTR.1;Parent=Glyma.06G182800.2.Wm82.a2.v1;pacid=30553595 |
| Chr06 | phytozomev10 | exon | 15706586 | 15708663 | . | - | . | ID=Glyma.06G182800.2.Wm82.a2.v1.exon.2;Parent=Glyma.06G182800.2.Wm82.a2.v1;pacid=30553595 |
| Chr06 | phytozomev10 | CDS | 15706586 | 15708636 | . | - | 0 | ID=Glyma.06G182800.2.Wm82.a2.v1.CDS.1;Parent=Glyma.06G182800.2.Wm82.a2.v1;pacid=30553595 |
| Chr06 | phytozomev10 | 5p_UTR | 15708637 | 15708663 | . | - | . | ID=Glyma.06G182800.2.Wm82.a2.v1.5p_UTR.2;Parent=Glyma.06G182800.2.Wm82.a2.v1;pacid=30553595 |
| Chr06 | phytozomev10 | exon | 15705310 | 15705840 | . | - | . | ID=Glyma.06G182800.2.Wm82.a2.v1.exon.3;Parent=Glyma.06G182800.2.Wm82.a2.v1;pacid=30553595 |
| Chr06 | phytozomev10 | 3p_UTR | 15705310 | 15705629 | . | - | . | ID=Glyma.06G182800.2.Wm82.a2.v1.3p_UTR.1;Parent=Glyma.06G182800.2.Wm82.a2.v1;pacid=30553595 |
| Chr06 | phytozomev10 | CDS | 15705630 | 15705840 | . | - | 1 | ID=Glyma.06G182800.2.Wm82.a2.v1.CDS.2;Parent=Glyma.06G182800.2.Wm82.a2.v1;pacid=30553595 |
| Chr06 | phytozomev10 | mRNA | 15705290 | 15709591 | . | - | . | ID=Glyma.06G182800.1.Wm82.a2.v1;Name=Glyma.06G182800.1;pacid=30553594;longest=1;ancestorIdentifer=Glyma06g19430.2.v1.1;Parent=Glyma.06G182800.Wm82.a2.v1 |
| Chr06 | phytozomev10 | exon | 15709207 | 15709591 | . | - | . | ID=Glyma.06G182800.1.Wm82.a2.v1.exon.1;Parent=Glyma.06G182800.1.Wm82.a2.v1;pacid=30553594 |
| Chr06 | phytozomev10 | 5p_UTR | 15709207 | 15709591 | . | - | . | ID=Glyma.06G182800.1.Wm82.a2.v1.5p_UTR.1;Parent=Glyma.06G182800.1.Wm82.a2.v1;pacid=30553594 |
| Chr06 | phytozomev10 | exon | 15706586 | 15708663 | . | - | . | ID=Glyma.06G182800.1.Wm82.a2.v1.exon.2;Parent=Glyma.06G182800.1.Wm82.a2.v1;pacid=30553594 |
| Chr06 | phytozomev10 | CDS | 15706586 | 15708636 | . | - | 0 | ID=Glyma.06G182800.1.Wm82.a2.v1.CDS.1;Parent=Glyma.06G182800.1.Wm82.a2.v1;pacid=30553594 |
| Chr06 | phytozomev10 | 5p_UTR | 15708637 | 15708663 | . | - | . | ID=Glyma.06G182800.1.Wm82.a2.v1.5p_UTR.2;Parent=Glyma.06G182800.1.Wm82.a2.v1;pacid=30553594 |
| Chr06 | phytozomev10 | exon | 15705290 | 15705840 | . | - | . | ID=Glyma.06G182800.1.Wm82.a2.v1.exon.3;Parent=Glyma.06G182800.1.Wm82.a2.v1;pacid=30553594 |
| Chr06 | phytozomev10 | 3p_UTR | 15705290 | 15705629 | . | - | . | ID=Glyma.06G182800.1.Wm82.a2.v1.3p_UTR.1;Parent=Glyma.06G182800.1.Wm82.a2.v1;pacid=30553594 |
| Chr06 | phytozomev10 | CDS | 15705630 | 15705840 | . | - | 1 | ID=Glyma.06G182800.1.Wm82.a2.v1.CDS.2;Parent=Glyma.06G182800.1.Wm82.a2.v1;pacid=30553594 |
| Chr06 | phytozomev10 | mRNA | 15705310 | 15708875 | . | - | . | ID=Glyma.06G182800.3.Wm82.a2.v1;Name=Glyma.06G182800.3;pacid=30553593;longest=0;Parent=Glyma.06G182800.Wm82.a2.v1 |
| Chr06 | phytozomev10 | exon | 15708783 | 15708875 | . | - | . | ID=Glyma.06G182800.3.Wm82.a2.v1.exon.1;Parent=Glyma.06G182800.3.Wm82.a2.v1;pacid=30553593 |
| Chr06 | phytozomev10 | 5p_UTR | 15708783 | 15708875 | . | - | . | ID=Glyma.06G182800.3.Wm82.a2.v1.5p_UTR.1;Parent=Glyma.06G182800.3.Wm82.a2.v1;pacid=30553593 |
| Chr06 | phytozomev10 | exon | 15706586 | 15708663 | . | - | . | ID=Glyma.06G182800.3.Wm82.a2.v1.exon.2;Parent=Glyma.06G182800.3.Wm82.a2.v1;pacid=30553593 |
| Chr06 | phytozomev10 | CDS | 15706586 | 15708636 | . | - | 0 | ID=Glyma.06G182800.3.Wm82.a2.v1.CDS.1;Parent=Glyma.06G182800.3.Wm82.a2.v1;pacid=30553593 |
| Chr06 | phytozomev10 | 5p_UTR | 15708637 | 15708663 | . | - | . | ID=Glyma.06G182800.3.Wm82.a2.v1.5p_UTR.2;Parent=Glyma.06G182800.3.Wm82.a2.v1;pacid=30553593 |
| Chr06 | phytozomev10 | exon | 15705310 | 15705840 | . | - | . | ID=Glyma.06G182800.3.Wm82.a2.v1.exon.3;Parent=Glyma.06G182800.3.Wm82.a2.v1;pacid=30553593 |
| Chr06 | phytozomev10 | 3p_UTR | 15705310 | 15705629 | . | - | . | ID=Glyma.06G182800.3.Wm82.a2.v1.3p_UTR.1;Parent=Glyma.06G182800.3.Wm82.a2.v1;pacid=30553593 |
| Chr06 | phytozomev10 | CDS | 15705630 | 15705840 | . | - | 1 | ID=Glyma.06G182800.3.Wm82.a2.v1.CDS.2;Parent=Glyma.06G182800.3.Wm82.a2.v1;pacid=30553593 |
| Chr07 | phytozomev10 | gene | 40298318 | 40302692 | . | - | . | ID=Glyma.07G226000.Wm82.a2.v1;Name=Glyma.07G226000;ancestorIdentifier=Glyma07g35240.v1.1 |
| Chr07 | phytozomev10 | mRNA | 40298318 | 40302692 | . | - | . | ID=Glyma.07G226000.1.Wm82.a2.v1;Name=Glyma.07G226000.1;pacid=30491884;longest=1;ancestorIdentifer=Glyma07g35240.1.v1.1;Parent=Glyma.07G226000.Wm82.a2.v1 |
| Chr07 | phytozomev10 | exon | 40302596 | 40302692 | . | - | . | ID=Glyma.07G226000.1.Wm82.a2.v1.exon.1;Parent=Glyma.07G226000.1.Wm82.a2.v1;pacid=30491884 |
| Chr07 | phytozomev10 | CDS | 40302596 | 40302668 | . | - | 0 | ID=Glyma.07G226000.1.Wm82.a2.v1.CDS.1;Parent=Glyma.07G226000.1.Wm82.a2.v1;pacid=30491884 |
| Chr07 | phytozomev10 | 5p_UTR | 40302669 | 40302692 | . | - | . | ID=Glyma.07G226000.1.Wm82.a2.v1.5p_UTR.1;Parent=Glyma.07G226000.1.Wm82.a2.v1;pacid=30491884 |
| Chr07 | phytozomev10 | exon | 40301689 | 40301995 | . | - | . | ID=Glyma.07G226000.1.Wm82.a2.v1.exon.2;Parent=Glyma.07G226000.1.Wm82.a2.v1;pacid=30491884 |
| Chr07 | phytozomev10 | CDS | 40301689 | 40301995 | . | - | 2 | ID=Glyma.07G226000.1.Wm82.a2.v1.CDS.2;Parent=Glyma.07G226000.1.Wm82.a2.v1;pacid=30491884 |
| Chr07 | phytozomev10 | exon | 40300833 | 40300920 | . | - | . | ID=Glyma.07G226000.1.Wm82.a2.v1.exon.3;Parent=Glyma.07G226000.1.Wm82.a2.v1;pacid=30491884 |
| Chr07 | phytozomev10 | CDS | 40300833 | 40300920 | . | - | 1 | ID=Glyma.07G226000.1.Wm82.a2.v1.CDS.3;Parent=Glyma.07G226000.1.Wm82.a2.v1;pacid=30491884 |
| Chr07 | phytozomev10 | exon | 40300609 | 40300718 | . | - | . | ID=Glyma.07G226000.1.Wm82.a2.v1.exon.4;Parent=Glyma.07G226000.1.Wm82.a2.v1;pacid=30491884 |
| Chr07 | phytozomev10 | CDS | 40300609 | 40300718 | . | - | 0 | ID=Glyma.07G226000.1.Wm82.a2.v1.CDS.4;Parent=Glyma.07G226000.1.Wm82.a2.v1;pacid=30491884 |
| Chr07 | phytozomev10 | exon | 40299763 | 40299911 | . | - | . | ID=Glyma.07G226000.1.Wm82.a2.v1.exon.5;Parent=Glyma.07G226000.1.Wm82.a2.v1;pacid=30491884 |
| Chr07 | phytozomev10 | CDS | 40299763 | 40299911 | . | - | 1 | ID=Glyma.07G226000.1.Wm82.a2.v1.CDS.5;Parent=Glyma.07G226000.1.Wm82.a2.v1;pacid=30491884 |
| Chr07 | phytozomev10 | exon | 40299103 | 40299149 | . | - | . | ID=Glyma.07G226000.1.Wm82.a2.v1.exon.6;Parent=Glyma.07G226000.1.Wm82.a2.v1;pacid=30491884 |
| Chr07 | phytozomev10 | CDS | 40299103 | 40299149 | . | - | 2 | ID=Glyma.07G226000.1.Wm82.a2.v1.CDS.6;Parent=Glyma.07G226000.1.Wm82.a2.v1;pacid=30491884 |
| Chr07 | phytozomev10 | exon | 40298318 | 40298662 | . | - | . | ID=Glyma.07G226000.1.Wm82.a2.v1.exon.7;Parent=Glyma.07G226000.1.Wm82.a2.v1;pacid=30491884 |
| Chr07 | phytozomev10 | 3p_UTR | 40298318 | 40298656 | . | - | . | ID=Glyma.07G226000.1.Wm82.a2.v1.3p_UTR.1;Parent=Glyma.07G226000.1.Wm82.a2.v1;pacid=30491884 |
| Chr07 | phytozomev10 | CDS | 40298657 | 40298662 | . | - | 0 | ID=Glyma.07G226000.1.Wm82.a2.v1.CDS.7;Parent=Glyma.07G226000.1.Wm82.a2.v1;pacid=30491884 |
| Chr08 | phytozomev10 | gene | 8877809 | 8881104 | . | + | . | ID=Glyma.08G115800.Wm82.a2.v1;Name=Glyma.08G115800;ancestorIdentifier=Glyma08g12220.v1.1 |
| Chr08 | phytozomev10 | mRNA | 8877809 | 8881104 | . | + | . | ID=Glyma.08G115800.2.Wm82.a2.v1;Name=Glyma.08G115800.2;pacid=30537328;longest=0;ancestorIdentifer=Glyma08g12220.2.v1.1;Parent=Glyma.08G115800.Wm82.a2.v1 |
| Chr08 | phytozomev10 | exon | 8877809 | 8878167 | . | + | . | ID=Glyma.08G115800.2.Wm82.a2.v1.exon.1;Parent=Glyma.08G115800.2.Wm82.a2.v1;pacid=30537328 |
| Chr08 | phytozomev10 | 5p_UTR | 8877809 | 8878094 | . | + | . | ID=Glyma.08G115800.2.Wm82.a2.v1.5p_UTR.1;Parent=Glyma.08G115800.2.Wm82.a2.v1;pacid=30537328 |
| Chr08 | phytozomev10 | CDS | 8878095 | 8878167 | . | + | 0 | ID=Glyma.08G115800.2.Wm82.a2.v1.CDS.1;Parent=Glyma.08G115800.2.Wm82.a2.v1;pacid=30537328 |
| Chr08 | phytozomev10 | exon | 8878374 | 8878680 | . | + | . | ID=Glyma.08G115800.2.Wm82.a2.v1.exon.2;Parent=Glyma.08G115800.2.Wm82.a2.v1;pacid=30537328 |
| Chr08 | phytozomev10 | CDS | 8878374 | 8878680 | . | + | 2 | ID=Glyma.08G115800.2.Wm82.a2.v1.CDS.2;Parent=Glyma.08G115800.2.Wm82.a2.v1;pacid=30537328 |
| Chr08 | phytozomev10 | exon | 8878785 | 8878872 | . | + | . | ID=Glyma.08G115800.2.Wm82.a2.v1.exon.3;Parent=Glyma.08G115800.2.Wm82.a2.v1;pacid=30537328 |
| Chr08 | phytozomev10 | CDS | 8878785 | 8878872 | . | + | 1 | ID=Glyma.08G115800.2.Wm82.a2.v1.CDS.3;Parent=Glyma.08G115800.2.Wm82.a2.v1;pacid=30537328 |
| Chr08 | phytozomev10 | exon | 8879493 | 8879602 | . | + | . | ID=Glyma.08G115800.2.Wm82.a2.v1.exon.4;Parent=Glyma.08G115800.2.Wm82.a2.v1;pacid=30537328 |
| Chr08 | phytozomev10 | CDS | 8879493 | 8879602 | . | + | 0 | ID=Glyma.08G115800.2.Wm82.a2.v1.CDS.4;Parent=Glyma.08G115800.2.Wm82.a2.v1;pacid=30537328 |
| Chr08 | phytozomev10 | exon | 8879723 | 8881104 | . | + | . | ID=Glyma.08G115800.2.Wm82.a2.v1.exon.5;Parent=Glyma.08G115800.2.Wm82.a2.v1;pacid=30537328 |
| Chr08 | phytozomev10 | CDS | 8879723 | 8879882 | . | + | 1 | ID=Glyma.08G115800.2.Wm82.a2.v1.CDS.5;Parent=Glyma.08G115800.2.Wm82.a2.v1;pacid=30537328 |
| Chr08 | phytozomev10 | 3p_UTR | 8879883 | 8881104 | . | + | . | ID=Glyma.08G115800.2.Wm82.a2.v1.3p_UTR.1;Parent=Glyma.08G115800.2.Wm82.a2.v1;pacid=30537328 |
| Chr08 | phytozomev10 | mRNA | 8877809 | 8881104 | . | + | . | ID=Glyma.08G115800.3.Wm82.a2.v1;Name=Glyma.08G115800.3;pacid=30537329;longest=0;ancestorIdentifer=Glyma08g12220.10.v1.1;Parent=Glyma.08G115800.Wm82.a2.v1 |
| Chr08 | phytozomev10 | exon | 8877809 | 8878167 | . | + | . | ID=Glyma.08G115800.3.Wm82.a2.v1.exon.1;Parent=Glyma.08G115800.3.Wm82.a2.v1;pacid=30537329 |
| Chr08 | phytozomev10 | 5p_UTR | 8877809 | 8878094 | . | + | . | ID=Glyma.08G115800.3.Wm82.a2.v1.5p_UTR.1;Parent=Glyma.08G115800.3.Wm82.a2.v1;pacid=30537329 |
| Chr08 | phytozomev10 | CDS | 8878095 | 8878167 | . | + | 0 | ID=Glyma.08G115800.3.Wm82.a2.v1.CDS.1;Parent=Glyma.08G115800.3.Wm82.a2.v1;pacid=30537329 |
| Chr08 | phytozomev10 | exon | 8878374 | 8878680 | . | + | . | ID=Glyma.08G115800.3.Wm82.a2.v1.exon.2;Parent=Glyma.08G115800.3.Wm82.a2.v1;pacid=30537329 |
| Chr08 | phytozomev10 | CDS | 8878374 | 8878680 | . | + | 2 | ID=Glyma.08G115800.3.Wm82.a2.v1.CDS.2;Parent=Glyma.08G115800.3.Wm82.a2.v1;pacid=30537329 |
| Chr08 | phytozomev10 | exon | 8878785 | 8878872 | . | + | . | ID=Glyma.08G115800.3.Wm82.a2.v1.exon.3;Parent=Glyma.08G115800.3.Wm82.a2.v1;pacid=30537329 |
| Chr08 | phytozomev10 | CDS | 8878785 | 8878872 | . | + | 1 | ID=Glyma.08G115800.3.Wm82.a2.v1.CDS.3;Parent=Glyma.08G115800.3.Wm82.a2.v1;pacid=30537329 |
| Chr08 | phytozomev10 | exon | 8879493 | 8879602 | . | + | . | ID=Glyma.08G115800.3.Wm82.a2.v1.exon.4;Parent=Glyma.08G115800.3.Wm82.a2.v1;pacid=30537329 |
| Chr08 | phytozomev10 | CDS | 8879493 | 8879602 | . | + | 0 | ID=Glyma.08G115800.3.Wm82.a2.v1.CDS.4;Parent=Glyma.08G115800.3.Wm82.a2.v1;pacid=30537329 |
| Chr08 | phytozomev10 | exon | 8879723 | 8880685 | . | + | . | ID=Glyma.08G115800.3.Wm82.a2.v1.exon.5;Parent=Glyma.08G115800.3.Wm82.a2.v1;pacid=30537329 |
| Chr08 | phytozomev10 | CDS | 8879723 | 8879882 | . | + | 1 | ID=Glyma.08G115800.3.Wm82.a2.v1.CDS.5;Parent=Glyma.08G115800.3.Wm82.a2.v1;pacid=30537329 |
| Chr08 | phytozomev10 | 3p_UTR | 8879883 | 8880685 | . | + | . | ID=Glyma.08G115800.3.Wm82.a2.v1.3p_UTR.1;Parent=Glyma.08G115800.3.Wm82.a2.v1;pacid=30537329 |
| Chr08 | phytozomev10 | exon | 8880763 | 8881104 | . | + | . | ID=Glyma.08G115800.3.Wm82.a2.v1.exon.6;Parent=Glyma.08G115800.3.Wm82.a2.v1;pacid=30537329 |
| Chr08 | phytozomev10 | 3p_UTR | 8880763 | 8881104 | . | + | . | ID=Glyma.08G115800.3.Wm82.a2.v1.3p_UTR.2;Parent=Glyma.08G115800.3.Wm82.a2.v1;pacid=30537329 |
| Chr08 | phytozomev10 | mRNA | 8878009 | 8881104 | . | + | . | ID=Glyma.08G115800.6.Wm82.a2.v1;Name=Glyma.08G115800.6;pacid=30537327;longest=0;Parent=Glyma.08G115800.Wm82.a2.v1 |
| Chr08 | phytozomev10 | exon | 8878009 | 8878167 | . | + | . | ID=Glyma.08G115800.6.Wm82.a2.v1.exon.1;Parent=Glyma.08G115800.6.Wm82.a2.v1;pacid=30537327 |
| Chr08 | phytozomev10 | 5p_UTR | 8878009 | 8878094 | . | + | . | ID=Glyma.08G115800.6.Wm82.a2.v1.5p_UTR.1;Parent=Glyma.08G115800.6.Wm82.a2.v1;pacid=30537327 |
| Chr08 | phytozomev10 | CDS | 8878095 | 8878167 | . | + | 0 | ID=Glyma.08G115800.6.Wm82.a2.v1.CDS.1;Parent=Glyma.08G115800.6.Wm82.a2.v1;pacid=30537327 |
| Chr08 | phytozomev10 | exon | 8878374 | 8878680 | . | + | . | ID=Glyma.08G115800.6.Wm82.a2.v1.exon.2;Parent=Glyma.08G115800.6.Wm82.a2.v1;pacid=30537327 |
| Chr08 | phytozomev10 | CDS | 8878374 | 8878680 | . | + | 2 | ID=Glyma.08G115800.6.Wm82.a2.v1.CDS.2;Parent=Glyma.08G115800.6.Wm82.a2.v1;pacid=30537327 |
| Chr08 | phytozomev10 | exon | 8878785 | 8878872 | . | + | . | ID=Glyma.08G115800.6.Wm82.a2.v1.exon.3;Parent=Glyma.08G115800.6.Wm82.a2.v1;pacid=30537327 |
| Chr08 | phytozomev10 | CDS | 8878785 | 8878872 | . | + | 1 | ID=Glyma.08G115800.6.Wm82.a2.v1.CDS.3;Parent=Glyma.08G115800.6.Wm82.a2.v1;pacid=30537327 |
| Chr08 | phytozomev10 | exon | 8879493 | 8879602 | . | + | . | ID=Glyma.08G115800.6.Wm82.a2.v1.exon.4;Parent=Glyma.08G115800.6.Wm82.a2.v1;pacid=30537327 |
| Chr08 | phytozomev10 | CDS | 8879493 | 8879602 | . | + | 0 | ID=Glyma.08G115800.6.Wm82.a2.v1.CDS.4;Parent=Glyma.08G115800.6.Wm82.a2.v1;pacid=30537327 |
| Chr08 | phytozomev10 | exon | 8879723 | 8879871 | . | + | . | ID=Glyma.08G115800.6.Wm82.a2.v1.exon.5;Parent=Glyma.08G115800.6.Wm82.a2.v1;pacid=30537327 |
| Chr08 | phytozomev10 | CDS | 8879723 | 8879871 | . | + | 1 | ID=Glyma.08G115800.6.Wm82.a2.v1.CDS.5;Parent=Glyma.08G115800.6.Wm82.a2.v1;pacid=30537327 |
| Chr08 | phytozomev10 | exon | 8880633 | 8881104 | . | + | . | ID=Glyma.08G115800.6.Wm82.a2.v1.exon.6;Parent=Glyma.08G115800.6.Wm82.a2.v1;pacid=30537327 |
| Chr08 | phytozomev10 | CDS | 8880633 | 8880685 | . | + | 2 | ID=Glyma.08G115800.6.Wm82.a2.v1.CDS.6;Parent=Glyma.08G115800.6.Wm82.a2.v1;pacid=30537327 |
| Chr08 | phytozomev10 | 3p_UTR | 8880686 | 8881104 | . | + | . | ID=Glyma.08G115800.6.Wm82.a2.v1.3p_UTR.1;Parent=Glyma.08G115800.6.Wm82.a2.v1;pacid=30537327 |
| Chr08 | phytozomev10 | mRNA | 8877809 | 8881104 | . | + | . | ID=Glyma.08G115800.1.Wm82.a2.v1;Name=Glyma.08G115800.1;pacid=30537326;longest=1;ancestorIdentifer=Glyma08g12220.8.v1.1;Parent=Glyma.08G115800.Wm82.a2.v1 |
| Chr08 | phytozomev10 | exon | 8877809 | 8878167 | . | + | . | ID=Glyma.08G115800.1.Wm82.a2.v1.exon.1;Parent=Glyma.08G115800.1.Wm82.a2.v1;pacid=30537326 |
| Chr08 | phytozomev10 | 5p_UTR | 8877809 | 8878094 | . | + | . | ID=Glyma.08G115800.1.Wm82.a2.v1.5p_UTR.1;Parent=Glyma.08G115800.1.Wm82.a2.v1;pacid=30537326 |
| Chr08 | phytozomev10 | CDS | 8878095 | 8878167 | . | + | 0 | ID=Glyma.08G115800.1.Wm82.a2.v1.CDS.1;Parent=Glyma.08G115800.1.Wm82.a2.v1;pacid=30537326 |
| Chr08 | phytozomev10 | exon | 8878374 | 8878680 | . | + | . | ID=Glyma.08G115800.1.Wm82.a2.v1.exon.2;Parent=Glyma.08G115800.1.Wm82.a2.v1;pacid=30537326 |
| Chr08 | phytozomev10 | CDS | 8878374 | 8878680 | . | + | 2 | ID=Glyma.08G115800.1.Wm82.a2.v1.CDS.2;Parent=Glyma.08G115800.1.Wm82.a2.v1;pacid=30537326 |
| Chr08 | phytozomev10 | exon | 8878785 | 8878872 | . | + | . | ID=Glyma.08G115800.1.Wm82.a2.v1.exon.3;Parent=Glyma.08G115800.1.Wm82.a2.v1;pacid=30537326 |
| Chr08 | phytozomev10 | CDS | 8878785 | 8878872 | . | + | 1 | ID=Glyma.08G115800.1.Wm82.a2.v1.CDS.3;Parent=Glyma.08G115800.1.Wm82.a2.v1;pacid=30537326 |
| Chr08 | phytozomev10 | exon | 8879493 | 8879602 | . | + | . | ID=Glyma.08G115800.1.Wm82.a2.v1.exon.4;Parent=Glyma.08G115800.1.Wm82.a2.v1;pacid=30537326 |
| Chr08 | phytozomev10 | CDS | 8879493 | 8879602 | . | + | 0 | ID=Glyma.08G115800.1.Wm82.a2.v1.CDS.4;Parent=Glyma.08G115800.1.Wm82.a2.v1;pacid=30537326 |
| Chr08 | phytozomev10 | exon | 8879723 | 8879871 | . | + | . | ID=Glyma.08G115800.1.Wm82.a2.v1.exon.5;Parent=Glyma.08G115800.1.Wm82.a2.v1;pacid=30537326 |
| Chr08 | phytozomev10 | CDS | 8879723 | 8879871 | . | + | 1 | ID=Glyma.08G115800.1.Wm82.a2.v1.CDS.5;Parent=Glyma.08G115800.1.Wm82.a2.v1;pacid=30537326 |
| Chr08 | phytozomev10 | exon | 8880633 | 8880685 | . | + | . | ID=Glyma.08G115800.1.Wm82.a2.v1.exon.6;Parent=Glyma.08G115800.1.Wm82.a2.v1;pacid=30537326 |
| Chr08 | phytozomev10 | CDS | 8880633 | 8880685 | . | + | 2 | ID=Glyma.08G115800.1.Wm82.a2.v1.CDS.6;Parent=Glyma.08G115800.1.Wm82.a2.v1;pacid=30537326 |
| Chr08 | phytozomev10 | exon | 8880763 | 8881104 | . | + | . | ID=Glyma.08G115800.1.Wm82.a2.v1.exon.7;Parent=Glyma.08G115800.1.Wm82.a2.v1;pacid=30537326 |
| Chr08 | phytozomev10 | 3p_UTR | 8880763 | 8881104 | . | + | . | ID=Glyma.08G115800.1.Wm82.a2.v1.3p_UTR.1;Parent=Glyma.08G115800.1.Wm82.a2.v1;pacid=30537326 |
| Chr08 | phytozomev10 | mRNA | 8877809 | 8881104 | . | + | . | ID=Glyma.08G115800.4.Wm82.a2.v1;Name=Glyma.08G115800.4;pacid=30537325;longest=0;ancestorIdentifer=Glyma08g12220.7.v1.1;Parent=Glyma.08G115800.Wm82.a2.v1 |
| Chr08 | phytozomev10 | exon | 8877809 | 8878167 | . | + | . | ID=Glyma.08G115800.4.Wm82.a2.v1.exon.1;Parent=Glyma.08G115800.4.Wm82.a2.v1;pacid=30537325 |
| Chr08 | phytozomev10 | 5p_UTR | 8877809 | 8878094 | . | + | . | ID=Glyma.08G115800.4.Wm82.a2.v1.5p_UTR.1;Parent=Glyma.08G115800.4.Wm82.a2.v1;pacid=30537325 |
| Chr08 | phytozomev10 | CDS | 8878095 | 8878167 | . | + | 0 | ID=Glyma.08G115800.4.Wm82.a2.v1.CDS.1;Parent=Glyma.08G115800.4.Wm82.a2.v1;pacid=30537325 |
| Chr08 | phytozomev10 | exon | 8878374 | 8878680 | . | + | . | ID=Glyma.08G115800.4.Wm82.a2.v1.exon.2;Parent=Glyma.08G115800.4.Wm82.a2.v1;pacid=30537325 |
| Chr08 | phytozomev10 | CDS | 8878374 | 8878680 | . | + | 2 | ID=Glyma.08G115800.4.Wm82.a2.v1.CDS.2;Parent=Glyma.08G115800.4.Wm82.a2.v1;pacid=30537325 |
| Chr08 | phytozomev10 | exon | 8878785 | 8878872 | . | + | . | ID=Glyma.08G115800.4.Wm82.a2.v1.exon.3;Parent=Glyma.08G115800.4.Wm82.a2.v1;pacid=30537325 |
| Chr08 | phytozomev10 | CDS | 8878785 | 8878872 | . | + | 1 | ID=Glyma.08G115800.4.Wm82.a2.v1.CDS.3;Parent=Glyma.08G115800.4.Wm82.a2.v1;pacid=30537325 |
| Chr08 | phytozomev10 | exon | 8879493 | 8879602 | . | + | . | ID=Glyma.08G115800.4.Wm82.a2.v1.exon.4;Parent=Glyma.08G115800.4.Wm82.a2.v1;pacid=30537325 |
| Chr08 | phytozomev10 | CDS | 8879493 | 8879602 | . | + | 0 | ID=Glyma.08G115800.4.Wm82.a2.v1.CDS.4;Parent=Glyma.08G115800.4.Wm82.a2.v1;pacid=30537325 |
| Chr08 | phytozomev10 | exon | 8879723 | 8879871 | . | + | . | ID=Glyma.08G115800.4.Wm82.a2.v1.exon.5;Parent=Glyma.08G115800.4.Wm82.a2.v1;pacid=30537325 |
| Chr08 | phytozomev10 | CDS | 8879723 | 8879871 | . | + | 1 | ID=Glyma.08G115800.4.Wm82.a2.v1.CDS.5;Parent=Glyma.08G115800.4.Wm82.a2.v1;pacid=30537325 |
| Chr08 | phytozomev10 | exon | 8880633 | 8880685 | . | + | . | ID=Glyma.08G115800.4.Wm82.a2.v1.exon.6;Parent=Glyma.08G115800.4.Wm82.a2.v1;pacid=30537325 |
| Chr08 | phytozomev10 | CDS | 8880633 | 8880685 | . | + | 2 | ID=Glyma.08G115800.4.Wm82.a2.v1.CDS.6;Parent=Glyma.08G115800.4.Wm82.a2.v1;pacid=30537325 |
| Chr08 | phytozomev10 | exon | 8880771 | 8881104 | . | + | . | ID=Glyma.08G115800.4.Wm82.a2.v1.exon.7;Parent=Glyma.08G115800.4.Wm82.a2.v1;pacid=30537325 |
| Chr08 | phytozomev10 | 3p_UTR | 8880771 | 8881104 | . | + | . | ID=Glyma.08G115800.4.Wm82.a2.v1.3p_UTR.1;Parent=Glyma.08G115800.4.Wm82.a2.v1;pacid=30537325 |
| Chr08 | phytozomev10 | mRNA | 8877809 | 8881104 | . | + | . | ID=Glyma.08G115800.5.Wm82.a2.v1;Name=Glyma.08G115800.5;pacid=30537324;longest=0;ancestorIdentifer=Glyma08g12220.9.v1.1;Parent=Glyma.08G115800.Wm82.a2.v1 |
| Chr08 | phytozomev10 | exon | 8877809 | 8878167 | . | + | . | ID=Glyma.08G115800.5.Wm82.a2.v1.exon.1;Parent=Glyma.08G115800.5.Wm82.a2.v1;pacid=30537324 |
| Chr08 | phytozomev10 | 5p_UTR | 8877809 | 8878094 | . | + | . | ID=Glyma.08G115800.5.Wm82.a2.v1.5p_UTR.1;Parent=Glyma.08G115800.5.Wm82.a2.v1;pacid=30537324 |
| Chr08 | phytozomev10 | CDS | 8878095 | 8878167 | . | + | 0 | ID=Glyma.08G115800.5.Wm82.a2.v1.CDS.1;Parent=Glyma.08G115800.5.Wm82.a2.v1;pacid=30537324 |
| Chr08 | phytozomev10 | exon | 8878374 | 8878680 | . | + | . | ID=Glyma.08G115800.5.Wm82.a2.v1.exon.2;Parent=Glyma.08G115800.5.Wm82.a2.v1;pacid=30537324 |
| Chr08 | phytozomev10 | CDS | 8878374 | 8878680 | . | + | 2 | ID=Glyma.08G115800.5.Wm82.a2.v1.CDS.2;Parent=Glyma.08G115800.5.Wm82.a2.v1;pacid=30537324 |
| Chr08 | phytozomev10 | exon | 8878785 | 8878872 | . | + | . | ID=Glyma.08G115800.5.Wm82.a2.v1.exon.3;Parent=Glyma.08G115800.5.Wm82.a2.v1;pacid=30537324 |
| Chr08 | phytozomev10 | CDS | 8878785 | 8878872 | . | + | 1 | ID=Glyma.08G115800.5.Wm82.a2.v1.CDS.3;Parent=Glyma.08G115800.5.Wm82.a2.v1;pacid=30537324 |
| Chr08 | phytozomev10 | exon | 8879493 | 8879602 | . | + | . | ID=Glyma.08G115800.5.Wm82.a2.v1.exon.4;Parent=Glyma.08G115800.5.Wm82.a2.v1;pacid=30537324 |
| Chr08 | phytozomev10 | CDS | 8879493 | 8879602 | . | + | 0 | ID=Glyma.08G115800.5.Wm82.a2.v1.CDS.4;Parent=Glyma.08G115800.5.Wm82.a2.v1;pacid=30537324 |
| Chr08 | phytozomev10 | exon | 8879723 | 8879871 | . | + | . | ID=Glyma.08G115800.5.Wm82.a2.v1.exon.5;Parent=Glyma.08G115800.5.Wm82.a2.v1;pacid=30537324 |
| Chr08 | phytozomev10 | CDS | 8879723 | 8879871 | . | + | 1 | ID=Glyma.08G115800.5.Wm82.a2.v1.CDS.5;Parent=Glyma.08G115800.5.Wm82.a2.v1;pacid=30537324 |
| Chr08 | phytozomev10 | exon | 8880633 | 8880685 | . | + | . | ID=Glyma.08G115800.5.Wm82.a2.v1.exon.6;Parent=Glyma.08G115800.5.Wm82.a2.v1;pacid=30537324 |
| Chr08 | phytozomev10 | CDS | 8880633 | 8880685 | . | + | 2 | ID=Glyma.08G115800.5.Wm82.a2.v1.CDS.6;Parent=Glyma.08G115800.5.Wm82.a2.v1;pacid=30537324 |
| Chr08 | phytozomev10 | exon | 8880768 | 8881104 | . | + | . | ID=Glyma.08G115800.5.Wm82.a2.v1.exon.7;Parent=Glyma.08G115800.5.Wm82.a2.v1;pacid=30537324 |
| Chr08 | phytozomev10 | 3p_UTR | 8880768 | 8881104 | . | + | . | ID=Glyma.08G115800.5.Wm82.a2.v1.3p_UTR.1;Parent=Glyma.08G115800.5.Wm82.a2.v1;pacid=30537324 |
| Chr08 | phytozomev10 | gene | 47528826 | 47532060 | . | - | . | ID=Glyma.08G363800.Wm82.a2.v1;Name=Glyma.08G363800;ancestorIdentifier=Glyma08g47900.v1.1 |
| Chr08 | phytozomev10 | mRNA | 47528826 | 47531719 | . | - | . | ID=Glyma.08G363800.3.Wm82.a2.v1;Name=Glyma.08G363800.3;pacid=30535568;longest=0;ancestorIdentifer=Glyma08g47900.3.v1.1;Parent=Glyma.08G363800.Wm82.a2.v1 |
| Chr08 | phytozomev10 | exon | 47531657 | 47531719 | . | - | . | ID=Glyma.08G363800.3.Wm82.a2.v1.exon.1;Parent=Glyma.08G363800.3.Wm82.a2.v1;pacid=30535568 |
| Chr08 | phytozomev10 | 5p_UTR | 47531657 | 47531719 | . | - | . | ID=Glyma.08G363800.3.Wm82.a2.v1.5p_UTR.1;Parent=Glyma.08G363800.3.Wm82.a2.v1;pacid=30535568 |
| Chr08 | phytozomev10 | exon | 47530729 | 47531239 | . | - | . | ID=Glyma.08G363800.3.Wm82.a2.v1.exon.2;Parent=Glyma.08G363800.3.Wm82.a2.v1;pacid=30535568 |
| Chr08 | phytozomev10 | CDS | 47530729 | 47531208 | . | - | 0 | ID=Glyma.08G363800.3.Wm82.a2.v1.CDS.1;Parent=Glyma.08G363800.3.Wm82.a2.v1;pacid=30535568 |
| Chr08 | phytozomev10 | 5p_UTR | 47531209 | 47531239 | . | - | . | ID=Glyma.08G363800.3.Wm82.a2.v1.5p_UTR.2;Parent=Glyma.08G363800.3.Wm82.a2.v1;pacid=30535568 |
| Chr08 | phytozomev10 | exon | 47530505 | 47530627 | . | - | . | ID=Glyma.08G363800.3.Wm82.a2.v1.exon.3;Parent=Glyma.08G363800.3.Wm82.a2.v1;pacid=30535568 |
| Chr08 | phytozomev10 | CDS | 47530505 | 47530627 | . | - | 0 | ID=Glyma.08G363800.3.Wm82.a2.v1.CDS.2;Parent=Glyma.08G363800.3.Wm82.a2.v1;pacid=30535568 |
| Chr08 | phytozomev10 | exon | 47529812 | 47529928 | . | - | . | ID=Glyma.08G363800.3.Wm82.a2.v1.exon.4;Parent=Glyma.08G363800.3.Wm82.a2.v1;pacid=30535568 |
| Chr08 | phytozomev10 | CDS | 47529812 | 47529928 | . | - | 0 | ID=Glyma.08G363800.3.Wm82.a2.v1.CDS.3;Parent=Glyma.08G363800.3.Wm82.a2.v1;pacid=30535568 |
| Chr08 | phytozomev10 | exon | 47528826 | 47529250 | . | - | . | ID=Glyma.08G363800.3.Wm82.a2.v1.exon.5;Parent=Glyma.08G363800.3.Wm82.a2.v1;pacid=30535568 |
| Chr08 | phytozomev10 | 3p_UTR | 47528826 | 47529196 | . | - | . | ID=Glyma.08G363800.3.Wm82.a2.v1.3p_UTR.1;Parent=Glyma.08G363800.3.Wm82.a2.v1;pacid=30535568 |
| Chr08 | phytozomev10 | CDS | 47529197 | 47529250 | . | - | 0 | ID=Glyma.08G363800.3.Wm82.a2.v1.CDS.4;Parent=Glyma.08G363800.3.Wm82.a2.v1;pacid=30535568 |
| Chr08 | phytozomev10 | mRNA | 47528826 | 47532060 | . | - | . | ID=Glyma.08G363800.2.Wm82.a2.v1;Name=Glyma.08G363800.2;pacid=30535567;longest=0;ancestorIdentifer=Glyma08g47900.2.v1.1;Parent=Glyma.08G363800.Wm82.a2.v1 |
| Chr08 | phytozomev10 | exon | 47531744 | 47532060 | . | - | . | ID=Glyma.08G363800.2.Wm82.a2.v1.exon.1;Parent=Glyma.08G363800.2.Wm82.a2.v1;pacid=30535567 |
| Chr08 | phytozomev10 | 5p_UTR | 47531744 | 47532060 | . | - | . | ID=Glyma.08G363800.2.Wm82.a2.v1.5p_UTR.1;Parent=Glyma.08G363800.2.Wm82.a2.v1;pacid=30535567 |
| Chr08 | phytozomev10 | exon | 47530729 | 47531239 | . | - | . | ID=Glyma.08G363800.2.Wm82.a2.v1.exon.2;Parent=Glyma.08G363800.2.Wm82.a2.v1;pacid=30535567 |
| Chr08 | phytozomev10 | CDS | 47530729 | 47531208 | . | - | 0 | ID=Glyma.08G363800.2.Wm82.a2.v1.CDS.1;Parent=Glyma.08G363800.2.Wm82.a2.v1;pacid=30535567 |
| Chr08 | phytozomev10 | 5p_UTR | 47531209 | 47531239 | . | - | . | ID=Glyma.08G363800.2.Wm82.a2.v1.5p_UTR.2;Parent=Glyma.08G363800.2.Wm82.a2.v1;pacid=30535567 |
| Chr08 | phytozomev10 | exon | 47530505 | 47530627 | . | - | . | ID=Glyma.08G363800.2.Wm82.a2.v1.exon.3;Parent=Glyma.08G363800.2.Wm82.a2.v1;pacid=30535567 |
| Chr08 | phytozomev10 | CDS | 47530505 | 47530627 | . | - | 0 | ID=Glyma.08G363800.2.Wm82.a2.v1.CDS.2;Parent=Glyma.08G363800.2.Wm82.a2.v1;pacid=30535567 |
| Chr08 | phytozomev10 | exon | 47529812 | 47529928 | . | - | . | ID=Glyma.08G363800.2.Wm82.a2.v1.exon.4;Parent=Glyma.08G363800.2.Wm82.a2.v1;pacid=30535567 |
| Chr08 | phytozomev10 | CDS | 47529812 | 47529928 | . | - | 0 | ID=Glyma.08G363800.2.Wm82.a2.v1.CDS.3;Parent=Glyma.08G363800.2.Wm82.a2.v1;pacid=30535567 |
| Chr08 | phytozomev10 | exon | 47528826 | 47529250 | . | - | . | ID=Glyma.08G363800.2.Wm82.a2.v1.exon.5;Parent=Glyma.08G363800.2.Wm82.a2.v1;pacid=30535567 |
| Chr08 | phytozomev10 | 3p_UTR | 47528826 | 47529196 | . | - | . | ID=Glyma.08G363800.2.Wm82.a2.v1.3p_UTR.1;Parent=Glyma.08G363800.2.Wm82.a2.v1;pacid=30535567 |
| Chr08 | phytozomev10 | CDS | 47529197 | 47529250 | . | - | 0 | ID=Glyma.08G363800.2.Wm82.a2.v1.CDS.4;Parent=Glyma.08G363800.2.Wm82.a2.v1;pacid=30535567 |
| Chr08 | phytozomev10 | mRNA | 47528826 | 47532060 | . | - | . | ID=Glyma.08G363800.1.Wm82.a2.v1;Name=Glyma.08G363800.1;pacid=30535566;longest=1;ancestorIdentifer=Glyma08g47900.1.v1.1;Parent=Glyma.08G363800.Wm82.a2.v1 |
| Chr08 | phytozomev10 | exon | 47531744 | 47532060 | . | - | . | ID=Glyma.08G363800.1.Wm82.a2.v1.exon.1;Parent=Glyma.08G363800.1.Wm82.a2.v1;pacid=30535566 |
| Chr08 | phytozomev10 | 5p_UTR | 47531744 | 47532060 | . | - | . | ID=Glyma.08G363800.1.Wm82.a2.v1.5p_UTR.1;Parent=Glyma.08G363800.1.Wm82.a2.v1;pacid=30535566 |
| Chr08 | phytozomev10 | exon | 47530729 | 47531239 | . | - | . | ID=Glyma.08G363800.1.Wm82.a2.v1.exon.2;Parent=Glyma.08G363800.1.Wm82.a2.v1;pacid=30535566 |
| Chr08 | phytozomev10 | CDS | 47530729 | 47531208 | . | - | 0 | ID=Glyma.08G363800.1.Wm82.a2.v1.CDS.1;Parent=Glyma.08G363800.1.Wm82.a2.v1;pacid=30535566 |
| Chr08 | phytozomev10 | 5p_UTR | 47531209 | 47531239 | . | - | . | ID=Glyma.08G363800.1.Wm82.a2.v1.5p_UTR.2;Parent=Glyma.08G363800.1.Wm82.a2.v1;pacid=30535566 |
| Chr08 | phytozomev10 | exon | 47530505 | 47530627 | . | - | . | ID=Glyma.08G363800.1.Wm82.a2.v1.exon.3;Parent=Glyma.08G363800.1.Wm82.a2.v1;pacid=30535566 |
| Chr08 | phytozomev10 | CDS | 47530505 | 47530627 | . | - | 0 | ID=Glyma.08G363800.1.Wm82.a2.v1.CDS.2;Parent=Glyma.08G363800.1.Wm82.a2.v1;pacid=30535566 |
| Chr08 | phytozomev10 | exon | 47528826 | 47529928 | . | - | . | ID=Glyma.08G363800.1.Wm82.a2.v1.exon.4;Parent=Glyma.08G363800.1.Wm82.a2.v1;pacid=30535566 |
| Chr08 | phytozomev10 | 3p_UTR | 47528826 | 47529748 | . | - | . | ID=Glyma.08G363800.1.Wm82.a2.v1.3p_UTR.1;Parent=Glyma.08G363800.1.Wm82.a2.v1;pacid=30535566 |
| Chr08 | phytozomev10 | CDS | 47529749 | 47529928 | . | - | 0 | ID=Glyma.08G363800.1.Wm82.a2.v1.CDS.3;Parent=Glyma.08G363800.1.Wm82.a2.v1;pacid=30535566 |
| Chr12 | phytozomev10 | gene | 36943077 | 36946491 | . | - | . | ID=Glyma.12G210400.Wm82.a2.v1;Name=Glyma.12G210400;ancestorIdentifier=Glyma12g33800.v1.1 |
| Chr12 | phytozomev10 | mRNA | 36943077 | 36946491 | . | - | . | ID=Glyma.12G210400.2.Wm82.a2.v1;Name=Glyma.12G210400.2;pacid=30545753;longest=0;ancestorIdentifer=Glyma12g33800.2.v1.1;Parent=Glyma.12G210400.Wm82.a2.v1 |
| Chr12 | phytozomev10 | exon | 36946270 | 36946491 | . | - | . | ID=Glyma.12G210400.2.Wm82.a2.v1.exon.1;Parent=Glyma.12G210400.2.Wm82.a2.v1;pacid=30545753 |
| Chr12 | phytozomev10 | CDS | 36946270 | 36946342 | . | - | 0 | ID=Glyma.12G210400.2.Wm82.a2.v1.CDS.1;Parent=Glyma.12G210400.2.Wm82.a2.v1;pacid=30545753 |
| Chr12 | phytozomev10 | 5p_UTR | 36946343 | 36946491 | . | - | . | ID=Glyma.12G210400.2.Wm82.a2.v1.5p_UTR.1;Parent=Glyma.12G210400.2.Wm82.a2.v1;pacid=30545753 |
| Chr12 | phytozomev10 | exon | 36945478 | 36945784 | . | - | . | ID=Glyma.12G210400.2.Wm82.a2.v1.exon.2;Parent=Glyma.12G210400.2.Wm82.a2.v1;pacid=30545753 |
| Chr12 | phytozomev10 | CDS | 36945478 | 36945784 | . | - | 2 | ID=Glyma.12G210400.2.Wm82.a2.v1.CDS.2;Parent=Glyma.12G210400.2.Wm82.a2.v1;pacid=30545753 |
| Chr12 | phytozomev10 | exon | 36945283 | 36945370 | . | - | . | ID=Glyma.12G210400.2.Wm82.a2.v1.exon.3;Parent=Glyma.12G210400.2.Wm82.a2.v1;pacid=30545753 |
| Chr12 | phytozomev10 | CDS | 36945283 | 36945370 | . | - | 1 | ID=Glyma.12G210400.2.Wm82.a2.v1.CDS.3;Parent=Glyma.12G210400.2.Wm82.a2.v1;pacid=30545753 |
| Chr12 | phytozomev10 | exon | 36944438 | 36944547 | . | - | . | ID=Glyma.12G210400.2.Wm82.a2.v1.exon.4;Parent=Glyma.12G210400.2.Wm82.a2.v1;pacid=30545753 |
| Chr12 | phytozomev10 | CDS | 36944438 | 36944547 | . | - | 0 | ID=Glyma.12G210400.2.Wm82.a2.v1.CDS.4;Parent=Glyma.12G210400.2.Wm82.a2.v1;pacid=30545753 |
| Chr12 | phytozomev10 | exon | 36944202 | 36944350 | . | - | . | ID=Glyma.12G210400.2.Wm82.a2.v1.exon.5;Parent=Glyma.12G210400.2.Wm82.a2.v1;pacid=30545753 |
| Chr12 | phytozomev10 | CDS | 36944202 | 36944350 | . | - | 1 | ID=Glyma.12G210400.2.Wm82.a2.v1.CDS.5;Parent=Glyma.12G210400.2.Wm82.a2.v1;pacid=30545753 |
| Chr12 | phytozomev10 | exon | 36943608 | 36943660 | . | - | . | ID=Glyma.12G210400.2.Wm82.a2.v1.exon.6;Parent=Glyma.12G210400.2.Wm82.a2.v1;pacid=30545753 |
| Chr12 | phytozomev10 | CDS | 36943608 | 36943660 | . | - | 2 | ID=Glyma.12G210400.2.Wm82.a2.v1.CDS.6;Parent=Glyma.12G210400.2.Wm82.a2.v1;pacid=30545753 |
| Chr12 | phytozomev10 | exon | 36943077 | 36943514 | . | - | . | ID=Glyma.12G210400.2.Wm82.a2.v1.exon.7;Parent=Glyma.12G210400.2.Wm82.a2.v1;pacid=30545753 |
| Chr12 | phytozomev10 | 3p_UTR | 36943077 | 36943511 | . | - | . | ID=Glyma.12G210400.2.Wm82.a2.v1.3p_UTR.1;Parent=Glyma.12G210400.2.Wm82.a2.v1;pacid=30545753 |
| Chr12 | phytozomev10 | CDS | 36943512 | 36943514 | . | - | 0 | ID=Glyma.12G210400.2.Wm82.a2.v1.CDS.7;Parent=Glyma.12G210400.2.Wm82.a2.v1;pacid=30545753 |
| Chr12 | phytozomev10 | mRNA | 36943077 | 36946491 | . | - | . | ID=Glyma.12G210400.1.Wm82.a2.v1;Name=Glyma.12G210400.1;pacid=30545752;longest=1;ancestorIdentifer=Glyma12g33800.1.v1.1;Parent=Glyma.12G210400.Wm82.a2.v1 |
| Chr12 | phytozomev10 | exon | 36946270 | 36946491 | . | - | . | ID=Glyma.12G210400.1.Wm82.a2.v1.exon.1;Parent=Glyma.12G210400.1.Wm82.a2.v1;pacid=30545752 |
| Chr12 | phytozomev10 | CDS | 36946270 | 36946342 | . | - | 0 | ID=Glyma.12G210400.1.Wm82.a2.v1.CDS.1;Parent=Glyma.12G210400.1.Wm82.a2.v1;pacid=30545752 |
| Chr12 | phytozomev10 | 5p_UTR | 36946343 | 36946491 | . | - | . | ID=Glyma.12G210400.1.Wm82.a2.v1.5p_UTR.1;Parent=Glyma.12G210400.1.Wm82.a2.v1;pacid=30545752 |
| Chr12 | phytozomev10 | exon | 36945478 | 36945784 | . | - | . | ID=Glyma.12G210400.1.Wm82.a2.v1.exon.2;Parent=Glyma.12G210400.1.Wm82.a2.v1;pacid=30545752 |
| Chr12 | phytozomev10 | CDS | 36945478 | 36945784 | . | - | 2 | ID=Glyma.12G210400.1.Wm82.a2.v1.CDS.2;Parent=Glyma.12G210400.1.Wm82.a2.v1;pacid=30545752 |
| Chr12 | phytozomev10 | exon | 36945283 | 36945370 | . | - | . | ID=Glyma.12G210400.1.Wm82.a2.v1.exon.3;Parent=Glyma.12G210400.1.Wm82.a2.v1;pacid=30545752 |
| Chr12 | phytozomev10 | CDS | 36945283 | 36945370 | . | - | 1 | ID=Glyma.12G210400.1.Wm82.a2.v1.CDS.3;Parent=Glyma.12G210400.1.Wm82.a2.v1;pacid=30545752 |
| Chr12 | phytozomev10 | exon | 36944438 | 36944547 | . | - | . | ID=Glyma.12G210400.1.Wm82.a2.v1.exon.4;Parent=Glyma.12G210400.1.Wm82.a2.v1;pacid=30545752 |
| Chr12 | phytozomev10 | CDS | 36944438 | 36944547 | . | - | 0 | ID=Glyma.12G210400.1.Wm82.a2.v1.CDS.4;Parent=Glyma.12G210400.1.Wm82.a2.v1;pacid=30545752 |
| Chr12 | phytozomev10 | exon | 36944202 | 36944350 | . | - | . | ID=Glyma.12G210400.1.Wm82.a2.v1.exon.5;Parent=Glyma.12G210400.1.Wm82.a2.v1;pacid=30545752 |
| Chr12 | phytozomev10 | CDS | 36944202 | 36944350 | . | - | 1 | ID=Glyma.12G210400.1.Wm82.a2.v1.CDS.5;Parent=Glyma.12G210400.1.Wm82.a2.v1;pacid=30545752 |
| Chr12 | phytozomev10 | exon | 36943608 | 36943663 | . | - | . | ID=Glyma.12G210400.1.Wm82.a2.v1.exon.6;Parent=Glyma.12G210400.1.Wm82.a2.v1;pacid=30545752 |
| Chr12 | phytozomev10 | CDS | 36943608 | 36943663 | . | - | 2 | ID=Glyma.12G210400.1.Wm82.a2.v1.CDS.6;Parent=Glyma.12G210400.1.Wm82.a2.v1;pacid=30545752 |
| Chr12 | phytozomev10 | exon | 36943077 | 36943514 | . | - | . | ID=Glyma.12G210400.1.Wm82.a2.v1.exon.7;Parent=Glyma.12G210400.1.Wm82.a2.v1;pacid=30545752 |
| Chr12 | phytozomev10 | 3p_UTR | 36943077 | 36943511 | . | - | . | ID=Glyma.12G210400.1.Wm82.a2.v1.3p_UTR.1;Parent=Glyma.12G210400.1.Wm82.a2.v1;pacid=30545752 |
| Chr12 | phytozomev10 | CDS | 36943512 | 36943514 | . | - | 0 | ID=Glyma.12G210400.1.Wm82.a2.v1.CDS.7;Parent=Glyma.12G210400.1.Wm82.a2.v1;pacid=30545752 |
| Chr12 | phytozomev10 | gene | 38919217 | 38923409 | . | - | . | ID=Glyma.12G229200.Wm82.a2.v1;Name=Glyma.12G229200;ancestorIdentifier=Glyma12g35820.v1.1 |
| Chr12 | phytozomev10 | mRNA | 38919217 | 38923409 | . | - | . | ID=Glyma.12G229200.1.Wm82.a2.v1;Name=Glyma.12G229200.1;pacid=30548376;longest=1;ancestorIdentifer=Glyma12g35820.1.v1.1;Parent=Glyma.12G229200.Wm82.a2.v1 |
| Chr12 | phytozomev10 | exon | 38923208 | 38923409 | . | - | . | ID=Glyma.12G229200.1.Wm82.a2.v1.exon.1;Parent=Glyma.12G229200.1.Wm82.a2.v1;pacid=30548376 |
| Chr12 | phytozomev10 | CDS | 38923208 | 38923298 | . | - | 0 | ID=Glyma.12G229200.1.Wm82.a2.v1.CDS.1;Parent=Glyma.12G229200.1.Wm82.a2.v1;pacid=30548376 |
| Chr12 | phytozomev10 | 5p_UTR | 38923299 | 38923409 | . | - | . | ID=Glyma.12G229200.1.Wm82.a2.v1.5p_UTR.1;Parent=Glyma.12G229200.1.Wm82.a2.v1;pacid=30548376 |
| Chr12 | phytozomev10 | exon | 38922179 | 38922485 | . | - | . | ID=Glyma.12G229200.1.Wm82.a2.v1.exon.2;Parent=Glyma.12G229200.1.Wm82.a2.v1;pacid=30548376 |
| Chr12 | phytozomev10 | CDS | 38922179 | 38922485 | . | - | 2 | ID=Glyma.12G229200.1.Wm82.a2.v1.CDS.2;Parent=Glyma.12G229200.1.Wm82.a2.v1;pacid=30548376 |
| Chr12 | phytozomev10 | exon | 38921976 | 38922063 | . | - | . | ID=Glyma.12G229200.1.Wm82.a2.v1.exon.3;Parent=Glyma.12G229200.1.Wm82.a2.v1;pacid=30548376 |
| Chr12 | phytozomev10 | CDS | 38921976 | 38922063 | . | - | 1 | ID=Glyma.12G229200.1.Wm82.a2.v1.CDS.3;Parent=Glyma.12G229200.1.Wm82.a2.v1;pacid=30548376 |
| Chr12 | phytozomev10 | exon | 38920388 | 38920497 | . | - | . | ID=Glyma.12G229200.1.Wm82.a2.v1.exon.4;Parent=Glyma.12G229200.1.Wm82.a2.v1;pacid=30548376 |
| Chr12 | phytozomev10 | CDS | 38920388 | 38920497 | . | - | 0 | ID=Glyma.12G229200.1.Wm82.a2.v1.CDS.4;Parent=Glyma.12G229200.1.Wm82.a2.v1;pacid=30548376 |
| Chr12 | phytozomev10 | exon | 38920167 | 38920315 | . | - | . | ID=Glyma.12G229200.1.Wm82.a2.v1.exon.5;Parent=Glyma.12G229200.1.Wm82.a2.v1;pacid=30548376 |
| Chr12 | phytozomev10 | CDS | 38920167 | 38920315 | . | - | 1 | ID=Glyma.12G229200.1.Wm82.a2.v1.CDS.5;Parent=Glyma.12G229200.1.Wm82.a2.v1;pacid=30548376 |
| Chr12 | phytozomev10 | exon | 38919217 | 38919587 | . | - | . | ID=Glyma.12G229200.1.Wm82.a2.v1.exon.6;Parent=Glyma.12G229200.1.Wm82.a2.v1;pacid=30548376 |
| Chr12 | phytozomev10 | 3p_UTR | 38919217 | 38919534 | . | - | . | ID=Glyma.12G229200.1.Wm82.a2.v1.3p_UTR.1;Parent=Glyma.12G229200.1.Wm82.a2.v1;pacid=30548376 |
| Chr12 | phytozomev10 | CDS | 38919535 | 38919587 | . | - | 2 | ID=Glyma.12G229200.1.Wm82.a2.v1.CDS.6;Parent=Glyma.12G229200.1.Wm82.a2.v1;pacid=30548376 |
| Chr12 | phytozomev10 | mRNA | 38919217 | 38923409 | . | - | . | ID=Glyma.12G229200.2.Wm82.a2.v1;Name=Glyma.12G229200.2;pacid=30548377;longest=0;ancestorIdentifer=Glyma12g35820.2.v1.1;Parent=Glyma.12G229200.Wm82.a2.v1 |
| Chr12 | phytozomev10 | exon | 38923208 | 38923409 | . | - | . | ID=Glyma.12G229200.2.Wm82.a2.v1.exon.1;Parent=Glyma.12G229200.2.Wm82.a2.v1;pacid=30548377 |
| Chr12 | phytozomev10 | 5p_UTR | 38923208 | 38923409 | . | - | . | ID=Glyma.12G229200.2.Wm82.a2.v1.5p_UTR.1;Parent=Glyma.12G229200.2.Wm82.a2.v1;pacid=30548377 |
| Chr12 | phytozomev10 | exon | 38922179 | 38922514 | . | - | . | ID=Glyma.12G229200.2.Wm82.a2.v1.exon.2;Parent=Glyma.12G229200.2.Wm82.a2.v1;pacid=30548377 |
| Chr12 | phytozomev10 | CDS | 38922179 | 38922483 | . | - | 0 | ID=Glyma.12G229200.2.Wm82.a2.v1.CDS.1;Parent=Glyma.12G229200.2.Wm82.a2.v1;pacid=30548377 |
| Chr12 | phytozomev10 | 5p_UTR | 38922484 | 38922514 | . | - | . | ID=Glyma.12G229200.2.Wm82.a2.v1.5p_UTR.2;Parent=Glyma.12G229200.2.Wm82.a2.v1;pacid=30548377 |
| Chr12 | phytozomev10 | exon | 38921976 | 38922063 | . | - | . | ID=Glyma.12G229200.2.Wm82.a2.v1.exon.3;Parent=Glyma.12G229200.2.Wm82.a2.v1;pacid=30548377 |
| Chr12 | phytozomev10 | CDS | 38921976 | 38922063 | . | - | 1 | ID=Glyma.12G229200.2.Wm82.a2.v1.CDS.2;Parent=Glyma.12G229200.2.Wm82.a2.v1;pacid=30548377 |
| Chr12 | phytozomev10 | exon | 38920388 | 38920497 | . | - | . | ID=Glyma.12G229200.2.Wm82.a2.v1.exon.4;Parent=Glyma.12G229200.2.Wm82.a2.v1;pacid=30548377 |
| Chr12 | phytozomev10 | CDS | 38920388 | 38920497 | . | - | 0 | ID=Glyma.12G229200.2.Wm82.a2.v1.CDS.3;Parent=Glyma.12G229200.2.Wm82.a2.v1;pacid=30548377 |
| Chr12 | phytozomev10 | exon | 38920167 | 38920315 | . | - | . | ID=Glyma.12G229200.2.Wm82.a2.v1.exon.5;Parent=Glyma.12G229200.2.Wm82.a2.v1;pacid=30548377 |
| Chr12 | phytozomev10 | CDS | 38920167 | 38920315 | . | - | 1 | ID=Glyma.12G229200.2.Wm82.a2.v1.CDS.4;Parent=Glyma.12G229200.2.Wm82.a2.v1;pacid=30548377 |
| Chr12 | phytozomev10 | exon | 38919217 | 38919587 | . | - | . | ID=Glyma.12G229200.2.Wm82.a2.v1.exon.6;Parent=Glyma.12G229200.2.Wm82.a2.v1;pacid=30548377 |
| Chr12 | phytozomev10 | 3p_UTR | 38919217 | 38919534 | . | - | . | ID=Glyma.12G229200.2.Wm82.a2.v1.3p_UTR.1;Parent=Glyma.12G229200.2.Wm82.a2.v1;pacid=30548377 |
| Chr12 | phytozomev10 | CDS | 38919535 | 38919587 | . | - | 2 | ID=Glyma.12G229200.2.Wm82.a2.v1.CDS.5;Parent=Glyma.12G229200.2.Wm82.a2.v1;pacid=30548377 |
| Chr13 | phytozomev10 | gene | 37265741 | 37269626 | . | + | . | ID=Glyma.13G270600.Wm82.a2.v1;Name=Glyma.13G270600;ancestorIdentifier=Glyma13g34580.v1.1 |
| Chr13 | phytozomev10 | mRNA | 37265741 | 37269626 | . | + | . | ID=Glyma.13G270600.1.Wm82.a2.v1;Name=Glyma.13G270600.1;pacid=30500698;longest=1;ancestorIdentifer=Glyma13g34580.2.v1.1;Parent=Glyma.13G270600.Wm82.a2.v1 |
| Chr13 | phytozomev10 | exon | 37265741 | 37265925 | . | + | . | ID=Glyma.13G270600.1.Wm82.a2.v1.exon.1;Parent=Glyma.13G270600.1.Wm82.a2.v1;pacid=30500698 |
| Chr13 | phytozomev10 | 5p_UTR | 37265741 | 37265849 | . | + | . | ID=Glyma.13G270600.1.Wm82.a2.v1.5p_UTR.1;Parent=Glyma.13G270600.1.Wm82.a2.v1;pacid=30500698 |
| Chr13 | phytozomev10 | CDS | 37265850 | 37265925 | . | + | 0 | ID=Glyma.13G270600.1.Wm82.a2.v1.CDS.1;Parent=Glyma.13G270600.1.Wm82.a2.v1;pacid=30500698 |
| Chr13 | phytozomev10 | exon | 37266720 | 37267026 | . | + | . | ID=Glyma.13G270600.1.Wm82.a2.v1.exon.2;Parent=Glyma.13G270600.1.Wm82.a2.v1;pacid=30500698 |
| Chr13 | phytozomev10 | CDS | 37266720 | 37267026 | . | + | 2 | ID=Glyma.13G270600.1.Wm82.a2.v1.CDS.2;Parent=Glyma.13G270600.1.Wm82.a2.v1;pacid=30500698 |
| Chr13 | phytozomev10 | exon | 37267135 | 37267222 | . | + | . | ID=Glyma.13G270600.1.Wm82.a2.v1.exon.3;Parent=Glyma.13G270600.1.Wm82.a2.v1;pacid=30500698 |
| Chr13 | phytozomev10 | CDS | 37267135 | 37267222 | . | + | 1 | ID=Glyma.13G270600.1.Wm82.a2.v1.CDS.3;Parent=Glyma.13G270600.1.Wm82.a2.v1;pacid=30500698 |
| Chr13 | phytozomev10 | exon | 37268346 | 37268455 | . | + | . | ID=Glyma.13G270600.1.Wm82.a2.v1.exon.4;Parent=Glyma.13G270600.1.Wm82.a2.v1;pacid=30500698 |
| Chr13 | phytozomev10 | CDS | 37268346 | 37268455 | . | + | 0 | ID=Glyma.13G270600.1.Wm82.a2.v1.CDS.4;Parent=Glyma.13G270600.1.Wm82.a2.v1;pacid=30500698 |
| Chr13 | phytozomev10 | exon | 37268528 | 37268676 | . | + | . | ID=Glyma.13G270600.1.Wm82.a2.v1.exon.5;Parent=Glyma.13G270600.1.Wm82.a2.v1;pacid=30500698 |
| Chr13 | phytozomev10 | CDS | 37268528 | 37268676 | . | + | 1 | ID=Glyma.13G270600.1.Wm82.a2.v1.CDS.5;Parent=Glyma.13G270600.1.Wm82.a2.v1;pacid=30500698 |
| Chr13 | phytozomev10 | exon | 37269190 | 37269626 | . | + | . | ID=Glyma.13G270600.1.Wm82.a2.v1.exon.6;Parent=Glyma.13G270600.1.Wm82.a2.v1;pacid=30500698 |
| Chr13 | phytozomev10 | CDS | 37269190 | 37269251 | . | + | 2 | ID=Glyma.13G270600.1.Wm82.a2.v1.CDS.6;Parent=Glyma.13G270600.1.Wm82.a2.v1;pacid=30500698 |
| Chr13 | phytozomev10 | 3p_UTR | 37269252 | 37269626 | . | + | . | ID=Glyma.13G270600.1.Wm82.a2.v1.3p_UTR.1;Parent=Glyma.13G270600.1.Wm82.a2.v1;pacid=30500698 |
| Chr13 | phytozomev10 | mRNA | 37265763 | 37269626 | . | + | . | ID=Glyma.13G270600.2.Wm82.a2.v1;Name=Glyma.13G270600.2;pacid=30500699;longest=0;ancestorIdentifer=Glyma13g34580.3.v1.1;Parent=Glyma.13G270600.Wm82.a2.v1 |
| Chr13 | phytozomev10 | exon | 37265763 | 37265960 | . | + | . | ID=Glyma.13G270600.2.Wm82.a2.v1.exon.1;Parent=Glyma.13G270600.2.Wm82.a2.v1;pacid=30500699 |
| Chr13 | phytozomev10 | 5p_UTR | 37265763 | 37265960 | . | + | . | ID=Glyma.13G270600.2.Wm82.a2.v1.5p_UTR.1;Parent=Glyma.13G270600.2.Wm82.a2.v1;pacid=30500699 |
| Chr13 | phytozomev10 | exon | 37266720 | 37267026 | . | + | . | ID=Glyma.13G270600.2.Wm82.a2.v1.exon.2;Parent=Glyma.13G270600.2.Wm82.a2.v1;pacid=30500699 |
| Chr13 | phytozomev10 | 5p_UTR | 37266720 | 37266721 | . | + | . | ID=Glyma.13G270600.2.Wm82.a2.v1.5p_UTR.2;Parent=Glyma.13G270600.2.Wm82.a2.v1;pacid=30500699 |
| Chr13 | phytozomev10 | CDS | 37266722 | 37267026 | . | + | 0 | ID=Glyma.13G270600.2.Wm82.a2.v1.CDS.1;Parent=Glyma.13G270600.2.Wm82.a2.v1;pacid=30500699 |
| Chr13 | phytozomev10 | exon | 37267135 | 37267222 | . | + | . | ID=Glyma.13G270600.2.Wm82.a2.v1.exon.3;Parent=Glyma.13G270600.2.Wm82.a2.v1;pacid=30500699 |
| Chr13 | phytozomev10 | CDS | 37267135 | 37267222 | . | + | 1 | ID=Glyma.13G270600.2.Wm82.a2.v1.CDS.2;Parent=Glyma.13G270600.2.Wm82.a2.v1;pacid=30500699 |
| Chr13 | phytozomev10 | exon | 37268346 | 37268455 | . | + | . | ID=Glyma.13G270600.2.Wm82.a2.v1.exon.4;Parent=Glyma.13G270600.2.Wm82.a2.v1;pacid=30500699 |
| Chr13 | phytozomev10 | CDS | 37268346 | 37268455 | . | + | 0 | ID=Glyma.13G270600.2.Wm82.a2.v1.CDS.3;Parent=Glyma.13G270600.2.Wm82.a2.v1;pacid=30500699 |
| Chr13 | phytozomev10 | exon | 37268528 | 37268676 | . | + | . | ID=Glyma.13G270600.2.Wm82.a2.v1.exon.5;Parent=Glyma.13G270600.2.Wm82.a2.v1;pacid=30500699 |
| Chr13 | phytozomev10 | CDS | 37268528 | 37268676 | . | + | 1 | ID=Glyma.13G270600.2.Wm82.a2.v1.CDS.4;Parent=Glyma.13G270600.2.Wm82.a2.v1;pacid=30500699 |
| Chr13 | phytozomev10 | exon | 37269190 | 37269626 | . | + | . | ID=Glyma.13G270600.2.Wm82.a2.v1.exon.6;Parent=Glyma.13G270600.2.Wm82.a2.v1;pacid=30500699 |
| Chr13 | phytozomev10 | CDS | 37269190 | 37269251 | . | + | 2 | ID=Glyma.13G270600.2.Wm82.a2.v1.CDS.5;Parent=Glyma.13G270600.2.Wm82.a2.v1;pacid=30500699 |
| Chr13 | phytozomev10 | 3p_UTR | 37269252 | 37269626 | . | + | . | ID=Glyma.13G270600.2.Wm82.a2.v1.3p_UTR.1;Parent=Glyma.13G270600.2.Wm82.a2.v1;pacid=30500699 |
| Chr13 | phytozomev10 | gene | 39120795 | 39124124 | . | + | . | ID=Glyma.13G290900.Wm82.a2.v1;Name=Glyma.13G290900;ancestorIdentifier=Glyma13g36690.v1.1 |
| Chr13 | phytozomev10 | mRNA | 39120795 | 39124124 | . | + | . | ID=Glyma.13G290900.2.Wm82.a2.v1;Name=Glyma.13G290900.2;pacid=30500145;longest=0;ancestorIdentifer=Glyma13g36690.2.v1.1;Parent=Glyma.13G290900.Wm82.a2.v1 |
| Chr13 | phytozomev10 | exon | 39120795 | 39120994 | . | + | . | ID=Glyma.13G290900.2.Wm82.a2.v1.exon.1;Parent=Glyma.13G290900.2.Wm82.a2.v1;pacid=30500145 |
| Chr13 | phytozomev10 | 5p_UTR | 39120795 | 39120921 | . | + | . | ID=Glyma.13G290900.2.Wm82.a2.v1.5p_UTR.1;Parent=Glyma.13G290900.2.Wm82.a2.v1;pacid=30500145 |
| Chr13 | phytozomev10 | CDS | 39120922 | 39120994 | . | + | 0 | ID=Glyma.13G290900.2.Wm82.a2.v1.CDS.1;Parent=Glyma.13G290900.2.Wm82.a2.v1;pacid=30500145 |
| Chr13 | phytozomev10 | exon | 39121463 | 39121769 | . | + | . | ID=Glyma.13G290900.2.Wm82.a2.v1.exon.2;Parent=Glyma.13G290900.2.Wm82.a2.v1;pacid=30500145 |
| Chr13 | phytozomev10 | CDS | 39121463 | 39121769 | . | + | 2 | ID=Glyma.13G290900.2.Wm82.a2.v1.CDS.2;Parent=Glyma.13G290900.2.Wm82.a2.v1;pacid=30500145 |
| Chr13 | phytozomev10 | exon | 39121881 | 39121968 | . | + | . | ID=Glyma.13G290900.2.Wm82.a2.v1.exon.3;Parent=Glyma.13G290900.2.Wm82.a2.v1;pacid=30500145 |
| Chr13 | phytozomev10 | CDS | 39121881 | 39121968 | . | + | 1 | ID=Glyma.13G290900.2.Wm82.a2.v1.CDS.3;Parent=Glyma.13G290900.2.Wm82.a2.v1;pacid=30500145 |
| Chr13 | phytozomev10 | exon | 39122714 | 39122823 | . | + | . | ID=Glyma.13G290900.2.Wm82.a2.v1.exon.4;Parent=Glyma.13G290900.2.Wm82.a2.v1;pacid=30500145 |
| Chr13 | phytozomev10 | CDS | 39122714 | 39122823 | . | + | 0 | ID=Glyma.13G290900.2.Wm82.a2.v1.CDS.4;Parent=Glyma.13G290900.2.Wm82.a2.v1;pacid=30500145 |
| Chr13 | phytozomev10 | exon | 39122909 | 39123057 | . | + | . | ID=Glyma.13G290900.2.Wm82.a2.v1.exon.5;Parent=Glyma.13G290900.2.Wm82.a2.v1;pacid=30500145 |
| Chr13 | phytozomev10 | CDS | 39122909 | 39123057 | . | + | 1 | ID=Glyma.13G290900.2.Wm82.a2.v1.CDS.5;Parent=Glyma.13G290900.2.Wm82.a2.v1;pacid=30500145 |
| Chr13 | phytozomev10 | exon | 39123604 | 39123656 | . | + | . | ID=Glyma.13G290900.2.Wm82.a2.v1.exon.6;Parent=Glyma.13G290900.2.Wm82.a2.v1;pacid=30500145 |
| Chr13 | phytozomev10 | CDS | 39123604 | 39123656 | . | + | 2 | ID=Glyma.13G290900.2.Wm82.a2.v1.CDS.6;Parent=Glyma.13G290900.2.Wm82.a2.v1;pacid=30500145 |
| Chr13 | phytozomev10 | exon | 39123742 | 39124124 | . | + | . | ID=Glyma.13G290900.2.Wm82.a2.v1.exon.7;Parent=Glyma.13G290900.2.Wm82.a2.v1;pacid=30500145 |
| Chr13 | phytozomev10 | CDS | 39123742 | 39123744 | . | + | 0 | ID=Glyma.13G290900.2.Wm82.a2.v1.CDS.7;Parent=Glyma.13G290900.2.Wm82.a2.v1;pacid=30500145 |
| Chr13 | phytozomev10 | 3p_UTR | 39123745 | 39124124 | . | + | . | ID=Glyma.13G290900.2.Wm82.a2.v1.3p_UTR.1;Parent=Glyma.13G290900.2.Wm82.a2.v1;pacid=30500145 |
| Chr13 | phytozomev10 | mRNA | 39120795 | 39124124 | . | + | . | ID=Glyma.13G290900.1.Wm82.a2.v1;Name=Glyma.13G290900.1;pacid=30500144;longest=1;ancestorIdentifer=Glyma13g36690.1.v1.1;Parent=Glyma.13G290900.Wm82.a2.v1 |
| Chr13 | phytozomev10 | exon | 39120795 | 39120994 | . | + | . | ID=Glyma.13G290900.1.Wm82.a2.v1.exon.1;Parent=Glyma.13G290900.1.Wm82.a2.v1;pacid=30500144 |
| Chr13 | phytozomev10 | 5p_UTR | 39120795 | 39120921 | . | + | . | ID=Glyma.13G290900.1.Wm82.a2.v1.5p_UTR.1;Parent=Glyma.13G290900.1.Wm82.a2.v1;pacid=30500144 |
| Chr13 | phytozomev10 | CDS | 39120922 | 39120994 | . | + | 0 | ID=Glyma.13G290900.1.Wm82.a2.v1.CDS.1;Parent=Glyma.13G290900.1.Wm82.a2.v1;pacid=30500144 |
| Chr13 | phytozomev10 | exon | 39121463 | 39121769 | . | + | . | ID=Glyma.13G290900.1.Wm82.a2.v1.exon.2;Parent=Glyma.13G290900.1.Wm82.a2.v1;pacid=30500144 |
| Chr13 | phytozomev10 | CDS | 39121463 | 39121769 | . | + | 2 | ID=Glyma.13G290900.1.Wm82.a2.v1.CDS.2;Parent=Glyma.13G290900.1.Wm82.a2.v1;pacid=30500144 |
| Chr13 | phytozomev10 | exon | 39121881 | 39121968 | . | + | . | ID=Glyma.13G290900.1.Wm82.a2.v1.exon.3;Parent=Glyma.13G290900.1.Wm82.a2.v1;pacid=30500144 |
| Chr13 | phytozomev10 | CDS | 39121881 | 39121968 | . | + | 1 | ID=Glyma.13G290900.1.Wm82.a2.v1.CDS.3;Parent=Glyma.13G290900.1.Wm82.a2.v1;pacid=30500144 |
| Chr13 | phytozomev10 | exon | 39122714 | 39122823 | . | + | . | ID=Glyma.13G290900.1.Wm82.a2.v1.exon.4;Parent=Glyma.13G290900.1.Wm82.a2.v1;pacid=30500144 |
| Chr13 | phytozomev10 | CDS | 39122714 | 39122823 | . | + | 0 | ID=Glyma.13G290900.1.Wm82.a2.v1.CDS.4;Parent=Glyma.13G290900.1.Wm82.a2.v1;pacid=30500144 |
| Chr13 | phytozomev10 | exon | 39122909 | 39123057 | . | + | . | ID=Glyma.13G290900.1.Wm82.a2.v1.exon.5;Parent=Glyma.13G290900.1.Wm82.a2.v1;pacid=30500144 |
| Chr13 | phytozomev10 | CDS | 39122909 | 39123057 | . | + | 1 | ID=Glyma.13G290900.1.Wm82.a2.v1.CDS.5;Parent=Glyma.13G290900.1.Wm82.a2.v1;pacid=30500144 |
| Chr13 | phytozomev10 | exon | 39123601 | 39123656 | . | + | . | ID=Glyma.13G290900.1.Wm82.a2.v1.exon.6;Parent=Glyma.13G290900.1.Wm82.a2.v1;pacid=30500144 |
| Chr13 | phytozomev10 | CDS | 39123601 | 39123656 | . | + | 2 | ID=Glyma.13G290900.1.Wm82.a2.v1.CDS.6;Parent=Glyma.13G290900.1.Wm82.a2.v1;pacid=30500144 |
| Chr13 | phytozomev10 | exon | 39123742 | 39124124 | . | + | . | ID=Glyma.13G290900.1.Wm82.a2.v1.exon.7;Parent=Glyma.13G290900.1.Wm82.a2.v1;pacid=30500144 |
| Chr13 | phytozomev10 | CDS | 39123742 | 39123744 | . | + | 0 | ID=Glyma.13G290900.1.Wm82.a2.v1.CDS.7;Parent=Glyma.13G290900.1.Wm82.a2.v1;pacid=30500144 |
| Chr13 | phytozomev10 | 3p_UTR | 39123745 | 39124124 | . | + | . | ID=Glyma.13G290900.1.Wm82.a2.v1.3p_UTR.1;Parent=Glyma.13G290900.1.Wm82.a2.v1;pacid=30500144 |
| Chr14 | phytozomev10 | gene | 43637893 | 43642553 | . | + | . | ID=Glyma.14G176900.Wm82.a2.v1;Name=Glyma.14G176900;ancestorIdentifier=Glyma14g35410.v1.1 |
| Chr14 | phytozomev10 | mRNA | 43640054 | 43642553 | . | + | . | ID=Glyma.14G176900.2.Wm82.a2.v1;Name=Glyma.14G176900.2;pacid=30534131;longest=0;ancestorIdentifer=Glyma14g35410.1.v1.1;Parent=Glyma.14G176900.Wm82.a2.v1 |
| Chr14 | phytozomev10 | exon | 43640054 | 43640710 | . | + | . | ID=Glyma.14G176900.2.Wm82.a2.v1.exon.1;Parent=Glyma.14G176900.2.Wm82.a2.v1;pacid=30534131 |
| Chr14 | phytozomev10 | 5p_UTR | 43640054 | 43640224 | . | + | . | ID=Glyma.14G176900.2.Wm82.a2.v1.5p_UTR.1;Parent=Glyma.14G176900.2.Wm82.a2.v1;pacid=30534131 |
| Chr14 | phytozomev10 | CDS | 43640225 | 43640710 | . | + | 0 | ID=Glyma.14G176900.2.Wm82.a2.v1.CDS.1;Parent=Glyma.14G176900.2.Wm82.a2.v1;pacid=30534131 |
| Chr14 | phytozomev10 | exon | 43641426 | 43641548 | . | + | . | ID=Glyma.14G176900.2.Wm82.a2.v1.exon.2;Parent=Glyma.14G176900.2.Wm82.a2.v1;pacid=30534131 |
| Chr14 | phytozomev10 | CDS | 43641426 | 43641548 | . | + | 0 | ID=Glyma.14G176900.2.Wm82.a2.v1.CDS.2;Parent=Glyma.14G176900.2.Wm82.a2.v1;pacid=30534131 |
| Chr14 | phytozomev10 | exon | 43641879 | 43641995 | . | + | . | ID=Glyma.14G176900.2.Wm82.a2.v1.exon.3;Parent=Glyma.14G176900.2.Wm82.a2.v1;pacid=30534131 |
| Chr14 | phytozomev10 | CDS | 43641879 | 43641995 | . | + | 0 | ID=Glyma.14G176900.2.Wm82.a2.v1.CDS.3;Parent=Glyma.14G176900.2.Wm82.a2.v1;pacid=30534131 |
| Chr14 | phytozomev10 | exon | 43642107 | 43642553 | . | + | . | ID=Glyma.14G176900.2.Wm82.a2.v1.exon.4;Parent=Glyma.14G176900.2.Wm82.a2.v1;pacid=30534131 |
| Chr14 | phytozomev10 | CDS | 43642107 | 43642166 | . | + | 0 | ID=Glyma.14G176900.2.Wm82.a2.v1.CDS.4;Parent=Glyma.14G176900.2.Wm82.a2.v1;pacid=30534131 |
| Chr14 | phytozomev10 | 3p_UTR | 43642167 | 43642553 | . | + | . | ID=Glyma.14G176900.2.Wm82.a2.v1.3p_UTR.1;Parent=Glyma.14G176900.2.Wm82.a2.v1;pacid=30534131 |
| Chr14 | phytozomev10 | mRNA | 43637893 | 43642553 | . | + | . | ID=Glyma.14G176900.1.Wm82.a2.v1;Name=Glyma.14G176900.1;pacid=30534130;longest=1;ancestorIdentifer=Glyma14g35410.2.v1.1;Parent=Glyma.14G176900.Wm82.a2.v1 |
| Chr14 | phytozomev10 | exon | 43637893 | 43638085 | . | + | . | ID=Glyma.14G176900.1.Wm82.a2.v1.exon.1;Parent=Glyma.14G176900.1.Wm82.a2.v1;pacid=30534130 |
| Chr14 | phytozomev10 | 5p_UTR | 43637893 | 43637937 | . | + | . | ID=Glyma.14G176900.1.Wm82.a2.v1.5p_UTR.1;Parent=Glyma.14G176900.1.Wm82.a2.v1;pacid=30534130 |
| Chr14 | phytozomev10 | CDS | 43637938 | 43638085 | . | + | 0 | ID=Glyma.14G176900.1.Wm82.a2.v1.CDS.1;Parent=Glyma.14G176900.1.Wm82.a2.v1;pacid=30534130 |
| Chr14 | phytozomev10 | exon | 43640214 | 43640710 | . | + | . | ID=Glyma.14G176900.1.Wm82.a2.v1.exon.2;Parent=Glyma.14G176900.1.Wm82.a2.v1;pacid=30534130 |
| Chr14 | phytozomev10 | CDS | 43640214 | 43640710 | . | + | 2 | ID=Glyma.14G176900.1.Wm82.a2.v1.CDS.2;Parent=Glyma.14G176900.1.Wm82.a2.v1;pacid=30534130 |
| Chr14 | phytozomev10 | exon | 43641426 | 43641548 | . | + | . | ID=Glyma.14G176900.1.Wm82.a2.v1.exon.3;Parent=Glyma.14G176900.1.Wm82.a2.v1;pacid=30534130 |
| Chr14 | phytozomev10 | CDS | 43641426 | 43641548 | . | + | 0 | ID=Glyma.14G176900.1.Wm82.a2.v1.CDS.3;Parent=Glyma.14G176900.1.Wm82.a2.v1;pacid=30534130 |
| Chr14 | phytozomev10 | exon | 43641879 | 43641995 | . | + | . | ID=Glyma.14G176900.1.Wm82.a2.v1.exon.4;Parent=Glyma.14G176900.1.Wm82.a2.v1;pacid=30534130 |
| Chr14 | phytozomev10 | CDS | 43641879 | 43641995 | . | + | 0 | ID=Glyma.14G176900.1.Wm82.a2.v1.CDS.4;Parent=Glyma.14G176900.1.Wm82.a2.v1;pacid=30534130 |
| Chr14 | phytozomev10 | exon | 43642107 | 43642553 | . | + | . | ID=Glyma.14G176900.1.Wm82.a2.v1.exon.5;Parent=Glyma.14G176900.1.Wm82.a2.v1;pacid=30534130 |
| Chr14 | phytozomev10 | CDS | 43642107 | 43642166 | . | + | 0 | ID=Glyma.14G176900.1.Wm82.a2.v1.CDS.5;Parent=Glyma.14G176900.1.Wm82.a2.v1;pacid=30534130 |
| Chr14 | phytozomev10 | 3p_UTR | 43642167 | 43642553 | . | + | . | ID=Glyma.14G176900.1.Wm82.a2.v1.3p_UTR.1;Parent=Glyma.14G176900.1.Wm82.a2.v1;pacid=30534130 |
| Chr17 | phytozomev10 | gene | 34108328 | 34108849 | . | - | . | ID=Glyma.17G208100.Wm82.a2.v1;Name=Glyma.17G208100;ancestorIdentifier=Glyma17g31305.v1.1 |
| Chr17 | phytozomev10 | mRNA | 34108328 | 34108849 | . | - | . | ID=Glyma.17G208100.1.Wm82.a2.v1;Name=Glyma.17G208100.1;pacid=30479727;longest=1;ancestorIdentifer=Glyma17g31305.1.v1.1;Parent=Glyma.17G208100.Wm82.a2.v1 |
| Chr17 | phytozomev10 | exon | 34108670 | 34108849 | . | - | . | ID=Glyma.17G208100.1.Wm82.a2.v1.exon.1;Parent=Glyma.17G208100.1.Wm82.a2.v1;pacid=30479727 |
| Chr17 | phytozomev10 | CDS | 34108670 | 34108849 | . | - | 0 | ID=Glyma.17G208100.1.Wm82.a2.v1.CDS.1;Parent=Glyma.17G208100.1.Wm82.a2.v1;pacid=30479727 |
| Chr17 | phytozomev10 | exon | 34108328 | 34108627 | . | - | . | ID=Glyma.17G208100.1.Wm82.a2.v1.exon.2;Parent=Glyma.17G208100.1.Wm82.a2.v1;pacid=30479727 |
| Chr17 | phytozomev10 | CDS | 34108328 | 34108627 | . | - | 0 | ID=Glyma.17G208100.1.Wm82.a2.v1.CDS.2;Parent=Glyma.17G208100.1.Wm82.a2.v1;pacid=30479727 |
| Chr18 | phytozomev10 | gene | 57587135 | 57590454 | . | + | . | ID=Glyma.18G298300.Wm82.a2.v1;Name=Glyma.18G298300;ancestorIdentifier=Glyma18g53610.v1.1 |
| Chr18 | phytozomev10 | mRNA | 57587135 | 57590454 | . | + | . | ID=Glyma.18G298300.1.Wm82.a2.v1;Name=Glyma.18G298300.1;pacid=30556705;longest=1;ancestorIdentifer=Glyma18g53610.1.v1.1;Parent=Glyma.18G298300.Wm82.a2.v1 |
| Chr18 | phytozomev10 | exon | 57587135 | 57587386 | . | + | . | ID=Glyma.18G298300.1.Wm82.a2.v1.exon.1;Parent=Glyma.18G298300.1.Wm82.a2.v1;pacid=30556705 |
| Chr18 | phytozomev10 | 5p_UTR | 57587135 | 57587386 | . | + | . | ID=Glyma.18G298300.1.Wm82.a2.v1.5p_UTR.1;Parent=Glyma.18G298300.1.Wm82.a2.v1;pacid=30556705 |
| Chr18 | phytozomev10 | exon | 57587761 | 57588271 | . | + | . | ID=Glyma.18G298300.1.Wm82.a2.v1.exon.2;Parent=Glyma.18G298300.1.Wm82.a2.v1;pacid=30556705 |
| Chr18 | phytozomev10 | 5p_UTR | 57587761 | 57587791 | . | + | . | ID=Glyma.18G298300.1.Wm82.a2.v1.5p_UTR.2;Parent=Glyma.18G298300.1.Wm82.a2.v1;pacid=30556705 |
| Chr18 | phytozomev10 | CDS | 57587792 | 57588271 | . | + | 0 | ID=Glyma.18G298300.1.Wm82.a2.v1.CDS.1;Parent=Glyma.18G298300.1.Wm82.a2.v1;pacid=30556705 |
| Chr18 | phytozomev10 | exon | 57588375 | 57588497 | . | + | . | ID=Glyma.18G298300.1.Wm82.a2.v1.exon.3;Parent=Glyma.18G298300.1.Wm82.a2.v1;pacid=30556705 |
| Chr18 | phytozomev10 | CDS | 57588375 | 57588497 | . | + | 0 | ID=Glyma.18G298300.1.Wm82.a2.v1.CDS.2;Parent=Glyma.18G298300.1.Wm82.a2.v1;pacid=30556705 |
| Chr18 | phytozomev10 | exon | 57589117 | 57589233 | . | + | . | ID=Glyma.18G298300.1.Wm82.a2.v1.exon.4;Parent=Glyma.18G298300.1.Wm82.a2.v1;pacid=30556705 |
| Chr18 | phytozomev10 | CDS | 57589117 | 57589233 | . | + | 0 | ID=Glyma.18G298300.1.Wm82.a2.v1.CDS.3;Parent=Glyma.18G298300.1.Wm82.a2.v1;pacid=30556705 |
| Chr18 | phytozomev10 | exon | 57589846 | 57590454 | . | + | . | ID=Glyma.18G298300.1.Wm82.a2.v1.exon.5;Parent=Glyma.18G298300.1.Wm82.a2.v1;pacid=30556705 |
| Chr18 | phytozomev10 | CDS | 57589846 | 57589899 | . | + | 0 | ID=Glyma.18G298300.1.Wm82.a2.v1.CDS.4;Parent=Glyma.18G298300.1.Wm82.a2.v1;pacid=30556705 |
| Chr18 | phytozomev10 | 3p_UTR | 57589900 | 57590454 | . | + | . | ID=Glyma.18G298300.1.Wm82.a2.v1.3p_UTR.1;Parent=Glyma.18G298300.1.Wm82.a2.v1;pacid=30556705 |
| Chr20 | phytozomev10 | gene | 2845106 | 2852380 | . | - | . | ID=Glyma.20G025900.Wm82.a2.v1;Name=Glyma.20G025900;ancestorIdentifier=Glyma20g03120.v1.1 |
| Chr20 | phytozomev10 | mRNA | 2845106 | 2852380 | . | - | . | ID=Glyma.20G025900.1.Wm82.a2.v1;Name=Glyma.20G025900.1;pacid=30521285;longest=1;ancestorIdentifer=Glyma20g03120.1.v1.1;Parent=Glyma.20G025900.Wm82.a2.v1 |
| Chr20 | phytozomev10 | exon | 2852241 | 2852380 | . | - | . | ID=Glyma.20G025900.1.Wm82.a2.v1.exon.1;Parent=Glyma.20G025900.1.Wm82.a2.v1;pacid=30521285 |
| Chr20 | phytozomev10 | CDS | 2852241 | 2852313 | . | - | 0 | ID=Glyma.20G025900.1.Wm82.a2.v1.CDS.1;Parent=Glyma.20G025900.1.Wm82.a2.v1;pacid=30521285 |
| Chr20 | phytozomev10 | 5p_UTR | 2852314 | 2852380 | . | - | . | ID=Glyma.20G025900.1.Wm82.a2.v1.5p_UTR.1;Parent=Glyma.20G025900.1.Wm82.a2.v1;pacid=30521285 |
| Chr20 | phytozomev10 | exon | 2851050 | 2851356 | . | - | . | ID=Glyma.20G025900.1.Wm82.a2.v1.exon.2;Parent=Glyma.20G025900.1.Wm82.a2.v1;pacid=30521285 |
| Chr20 | phytozomev10 | CDS | 2851050 | 2851356 | . | - | 2 | ID=Glyma.20G025900.1.Wm82.a2.v1.CDS.2;Parent=Glyma.20G025900.1.Wm82.a2.v1;pacid=30521285 |
| Chr20 | phytozomev10 | exon | 2850210 | 2850297 | . | - | . | ID=Glyma.20G025900.1.Wm82.a2.v1.exon.3;Parent=Glyma.20G025900.1.Wm82.a2.v1;pacid=30521285 |
| Chr20 | phytozomev10 | CDS | 2850210 | 2850297 | . | - | 1 | ID=Glyma.20G025900.1.Wm82.a2.v1.CDS.3;Parent=Glyma.20G025900.1.Wm82.a2.v1;pacid=30521285 |
| Chr20 | phytozomev10 | exon | 2849961 | 2850070 | . | - | . | ID=Glyma.20G025900.1.Wm82.a2.v1.exon.4;Parent=Glyma.20G025900.1.Wm82.a2.v1;pacid=30521285 |
| Chr20 | phytozomev10 | CDS | 2849961 | 2850070 | . | - | 0 | ID=Glyma.20G025900.1.Wm82.a2.v1.CDS.4;Parent=Glyma.20G025900.1.Wm82.a2.v1;pacid=30521285 |
| Chr20 | phytozomev10 | exon | 2846519 | 2846667 | . | - | . | ID=Glyma.20G025900.1.Wm82.a2.v1.exon.5;Parent=Glyma.20G025900.1.Wm82.a2.v1;pacid=30521285 |
| Chr20 | phytozomev10 | CDS | 2846519 | 2846667 | . | - | 1 | ID=Glyma.20G025900.1.Wm82.a2.v1.CDS.5;Parent=Glyma.20G025900.1.Wm82.a2.v1;pacid=30521285 |
| Chr20 | phytozomev10 | exon | 2845905 | 2845951 | . | - | . | ID=Glyma.20G025900.1.Wm82.a2.v1.exon.6;Parent=Glyma.20G025900.1.Wm82.a2.v1;pacid=30521285 |
| Chr20 | phytozomev10 | CDS | 2845905 | 2845951 | . | - | 2 | ID=Glyma.20G025900.1.Wm82.a2.v1.CDS.6;Parent=Glyma.20G025900.1.Wm82.a2.v1;pacid=30521285 |
| Chr20 | phytozomev10 | exon | 2845106 | 2845465 | . | - | . | ID=Glyma.20G025900.1.Wm82.a2.v1.exon.7;Parent=Glyma.20G025900.1.Wm82.a2.v1;pacid=30521285 |
| Chr20 | phytozomev10 | 3p_UTR | 2845106 | 2845456 | . | - | . | ID=Glyma.20G025900.1.Wm82.a2.v1.3p_UTR.1;Parent=Glyma.20G025900.1.Wm82.a2.v1;pacid=30521285 |
| Chr20 | phytozomev10 | CDS | 2845457 | 2845465 | . | - | 0 | ID=Glyma.20G025900.1.Wm82.a2.v1.CDS.7;Parent=Glyma.20G025900.1.Wm82.a2.v1;pacid=30521285 |
| Chr20 | phytozomev10 | gene | 7939112 | 7939943 | . | - | . | ID=Glyma.20G043700.Wm82.a2.v1;Name=Glyma.20G043700 |
| Chr20 | phytozomev10 | mRNA | 7939112 | 7939943 | . | - | . | ID=Glyma.20G043700.1.Wm82.a2.v1;Name=Glyma.20G043700.1;pacid=30521067;longest=1;Parent=Glyma.20G043700.Wm82.a2.v1 |
| Chr20 | phytozomev10 | exon | 7939770 | 7939943 | . | - | . | ID=Glyma.20G043700.1.Wm82.a2.v1.exon.1;Parent=Glyma.20G043700.1.Wm82.a2.v1;pacid=30521067 |
| Chr20 | phytozomev10 | CDS | 7939770 | 7939880 | . | - | 0 | ID=Glyma.20G043700.1.Wm82.a2.v1.CDS.1;Parent=Glyma.20G043700.1.Wm82.a2.v1;pacid=30521067 |
| Chr20 | phytozomev10 | 5p_UTR | 7939881 | 7939943 | . | - | . | ID=Glyma.20G043700.1.Wm82.a2.v1.5p_UTR.1;Parent=Glyma.20G043700.1.Wm82.a2.v1;pacid=30521067 |
| Chr20 | phytozomev10 | exon | 7939112 | 7939213 | . | - | . | ID=Glyma.20G043700.1.Wm82.a2.v1.exon.2;Parent=Glyma.20G043700.1.Wm82.a2.v1;pacid=30521067 |
| Chr20 | phytozomev10 | CDS | 7939112 | 7939213 | . | - | 0 | ID=Glyma.20G043700.1.Wm82.a2.v1.CDS.2;Parent=Glyma.20G043700.1.Wm82.a2.v1;pacid=30521067 |
