# Supplementary material for: Genome-wide identification and expression analysis of the 14-3-3 gene family in soybean (Glycine max)
Source: PeerJ. 2019 Dec 6;7:e7950. doi: 10.7717/peerj.7950 (PMC6901008; doi:10.7717/peerj.7950)
Supplement: Table S4 [file peerj-07-7950-s006.docx]

| Table S4 Duplication relationship of *GmGF14* genes | | |
| --- | --- | --- |
| Gene1 | Gene2 | Similarity |
| GmGF14a | GmGF14b | 98.462 |
| GmGF14a | GmGF14k | 89.655 |
| GmGF14a | GmGF14u | 89.844 |
| GmGF14b | GmGF14k | 89.655 |
| GmGF14b | GmGF14u | 90.234 |
| GmGF14c | GmGF14e | 83.972 |
| GmGF14c | GmGF14r | 100 |
| GmGF14d | GmGF14h | 97.211 |
| GmGF14f | GmGF14j | 88.064 |
| GmGF14g | GmGF14l | 96.538 |
| GmGF14g | GmGF14n | 81.923 |
| GmGF14g | GmGF14q | 80.242 |
| GmGF14k | GmGF14u | 98.842 |
| GmGF14l | GmGF14n | 83.846 |
| GmGF14l | GmGF14q | 83.077 |
| GmGF14m | GmGF14t | 99.583 |
| GmGF14n | GmGF14q | 98.092 |
| GmGF14o | GmGF14p | 95.455 |
| GmGF14r | GmGF14e | 84.028 |
