# Supplementary material for: Genome-wide identification and expression analysis of the 14-3-3 gene family in soybean (Glycine max)
Source: PeerJ. 2019 Dec 6;7:e7950. doi: 10.7717/peerj.7950 (PMC6901008; doi:10.7717/peerj.7950)
Supplement: Table S5 [file peerj-07-7950-s007.docx]

| Table S5 Similarity of duplication gene pairs under abotic stresses | | | | |
| --- | --- | --- | --- | --- |
| Gene1 | Gene2 | cold | drought | salt |
| GmGF14a | GmGF14b | √ | √ | √ |
| GmGF14a | GmGF14k |  |  |  |
| GmGF14a | GmGF14u |  |  |  |
| GmGF14b | GmGF14k |  |  |  |
| GmGF14b | GmGF14u |  |  |  |
| GmGF14c | GmGF14e | √ |  |  |
| GmGF14c | GmGF14r | √ | √ | √ |
| GmGF14d | GmGF14h |  |  |  |
| GmGF14f | GmGF14j |  | √ |  |
| GmGF14g | GmGF14l | √ |  |  |
| GmGF14g | GmGF14n | √ |  |  |
| GmGF14g | GmGF14q | √ |  | √ |
| GmGF14k | GmGF14u |  |  |  |
| GmGF14l | GmGF14n | √ |  |  |
| GmGF14l | GmGF14q | √ |  |  |
| GmGF14m | GmGF14t | √ |  |  |
| GmGF14n | GmGF14q | √ | √ |  |
| GmGF14o | GmGF14p |  | √ |  |
| GmGF14r | GmGF14e | √ |  | √ |
