# Supplementary material for: Genome-wide identification and expression analysis of the 14-3-3 gene family in soybean (Glycine max)
Source: PeerJ. 2019 Dec 6;7:e7950. doi: 10.7717/peerj.7950 (PMC6901008; doi:10.7717/peerj.7950)
Supplement: Table S6 [file peerj-07-7950-s008.docx]

| Table S6 Raw data for the drought stress | | | | | | | | | | | | |
| --- | --- | --- | --- | --- | --- | --- | --- | --- | --- | --- | --- | --- |
| Gene | 0h | | | 1h | | | 6h | | | 12h | | |
| internal control gene | 24.7424 | 25.4777 | 25.8974 | 24.7255 | 24.7774 | 24.6982 | 26.0245 | 25.7375 | 26.9825 | 24.8577 | 25.1777 | 24.7789 |
| GmGF14a | 28.4799 | 28.6596 | 28.8576 | 26.0018 | 26.2560 | 25.7486 | 27.9187 | 27.7983 | 27.7272 | 27.4636 | 27.6301 | 28.0052 |
| GmGF14b | 30.4555 | 30.6321 | 30.8321 | 27.9995 | 27.8308 | 27.9649 | 29.8129 | 29.6766 | 29.7312 | 29.1919 | 29.1916 | 28.8727 |
| GmGF14c | 28.5833 | 28.7712 | 28.6938 | 28.3131 | 28.3735 | 28.2248 | 29.9495 | 30.0588 | 30.0040 | 28.8182 | 28.8136 | 29.0454 |
| GmGF14d | 32.4269 | 32.4766 | 32.4518 | 31.9919 | 32.0835 | 32.2182 | 33.7893 | 33.8529 | 33.8211 | 32.7963 | 32.9897 | 32.8930 |
| GmGF14f | 32.7269 | 32.7766 | 32.8766 | 29.9636 | 29.9069 | 30.1398 | 32.4254 | 32.6384 | 32.5675 | 33.0931 | 33.0456 | 33.2797 |
| GmGF14e | 28.6734 | 28.6953 | 28.6764 | 28.8192 | 29.0471 | 28.8484 | 29.8512 | 29.7884 | 29.8415 | 28.7754 | 28.6764 | 28.6704 |
| GmGF14g | 30.6951 | 30.6929 | 30.5316 | 27.8681 | 27.8788 | 27.8073 | 29.9101 | 29.8883 | 29.9364 | 29.0367 | 29.2973 | 29.2388 |
| GmGF14h | 28.5520 | 28.8498 | 28.6797 | 27.0050 | 26.6579 | 27.0110 | 29.8161 | 29.7252 | 29.8849 | 25.8759 | 25.7024 | 25.8864 |
| GmGF14i | 31.4059 | 31.4868 | 31.4614 | 31.0816 | 31.0864 | 31.1567 | 31.9373 | 32.0322 | 31.8634 | 31.6214 | 31.7398 | 31.6247 |
| GmGF14j | 32.0896 | 32.2177 | 32.3296 | 32.4581 | 32.4594 | 32.2222 | 33.5443 | 33.9598 | 33.9384 | 33.5292 | 33.9741 | 33.5179 |
| GmGF14k | 28.6541 | 28.8151 | 28.7542 | 28.3472 | 28.3488 | 28.3905 | 30.5767 | 30.4720 | 30.4205 | 29.3166 | 29.8199 | 29.5350 |
| GmGF14l | 28.7118 | 28.8728 | 28.6424 | 28.2358 | 28.2768 | 28.2977 | 29.4062 | 29.3848 | 29.5113 | 27.6743 | 27.7005 | 27.5633 |
| GmGF14m | 28.7244 | 28.6601 | 28.5888 | 29.5445 | 29.0239 | 29.0853 | 30.0879 | 30.2608 | 30.0746 | 28.7110 | 28.7067 | 28.6098 |
| GmGF14n | 33.6778 | 33.7411 | 33.5887 | 33.0526 | 33.0106 | 33.0443 | 34.7995 | 34.7675 | 34.9697 | 33.7151 | 33.6749 | 33.8757 |
| GmGF14o | 33.8014 | 33.6691 | 33.8791 | 33.5363 | 33.6577 | 33.5429 | 34.2948 | 34.3427 | 34.2893 | 33.5751 | 33.4751 | 33.3751 |
| GmGF14p | 28.8060 | 28.6364 | 28.6954 | 29.1377 | 29.2080 | 29.0354 | 29.8422 | 29.9440 | 30.0982 | 28.9210 | 28.9149 | 29.0137 |
| GmGF14q | 28.6165 | 28.6634 | 28.5646 | 28.1173 | 28.0036 | 28.0896 | 29.5971 | 29.6691 | 29.6971 | 28.4213 | 28.5491 | 28.4123 |
| GmGF14r | 28.5834 | 28.6303 | 28.6723 | 28.0012 | 28.0868 | 28.0596 | 29.6442 | 29.6671 | 29.7030 | 28.4998 | 28.4436 | 28.5851 |
| GmGF14t | 33.6407 | 33.6876 | 33.7296 | 32.2999 | 32.2822 | 32.1977 | 34.8730 | 34.7605 | 34.8872 | 33.2695 | 33.3605 | 33.4229 |
