# Supplementary material for: Genome-wide identification and expression analysis of the 14-3-3 gene family in soybean (Glycine max)
Source: PeerJ. 2019 Dec 6;7:e7950. doi: 10.7717/peerj.7950 (PMC6901008; doi:10.7717/peerj.7950)
Supplement: Table S8 [file peerj-07-7950-s010.docx]

| Table S8 Raw data for the cold stress | | | | | | | | | | | | |
| --- | --- | --- | --- | --- | --- | --- | --- | --- | --- | --- | --- | --- |
| Gene | 0h | | | 1h | | | 6h | | | 12h | | |
| internal control gene | 24.7424 | 25.4777 | 25.8974 | 24.7255 | 24.7774 | 24.6982 | 26.0245 | 25.7375 | 26.9825 | 24.8577 | 25.1777 | 24.7789 |
| GmGF14a | 28.6127 | 28.6596 | 28.7016 | 28.2015 | 28.1223 | 28.1226 | 30.4772 | 30.2941 | 30.4973 | 29.4075 | 29.2540 | 29.4123 |
| GmGF14b | 30.5884 | 30.6321 | 30.6760 | 30.0313 | 30.1022 | 30.0019 | 33.1219 | 32.8426 | 33.0707 | 31.3647 | 31.4900 | 31.4327 |
| GmGF14c | 28.5833 | 28.7712 | 28.6938 | 28.0098 | 27.9188 | 27.9106 | 30.1730 | 30.1693 | 30.1711 | 29.2135 | 29.1846 | 29.1991 |
| GmGF14d | 32.4269 | 32.4766 | 32.4518 | 31.6598 | 31.6681 | 31.6066 | 33.8702 | 33.8829 | 33.8765 | 32.8963 | 32.8897 | 32.8930 |
| GmGF14f | 32.7725 | 32.7944 | 32.7613 | 31.5183 | 31.4837 | 31.5475 | 32.6502 | 32.6369 | 32.5715 | 31.8745 | 31.9555 | 31.9295 |
| GmGF14e | 28.6278 | 28.6775 | 28.7775 | 28.0096 | 27.9896 | 27.8600 | 30.3887 | 30.3292 | 30.5561 | 29.4470 | 29.2795 | 29.4606 |
| GmGF14g | 30.6951 | 30.6929 | 30.5316 | 28.8250 | 28.7425 | 28.7994 | 31.8101 | 31.6712 | 31.7564 | 30.2867 | 30.2973 | 30.3727 |
| GmGF14h | 28.6890 | 28.6974 | 28.6797 | 27.2455 | 27.1637 | 27.2595 | 29.7761 | 29.7854 | 29.8794 | 27.9376 | 27.9242 | 27.9290 |
| GmGF14i | 31.3244 | 31.4868 | 31.4760 | 30.8999 | 30.9164 | 30.9647 | 32.5494 | 32.5549 | 32.6467 | 32.7793 | 33.2935 | 32.7010 |
| GmGF14j | 32.0896 | 32.0717 | 32.1724 | 33.0735 | 33.1355 | 32.8142 | 33.5040 | 33.5239 | 33.4173 | 31.7292 | 31.8788 | 31.7200 |
| GmGF14k | 28.6541 | 28.8151 | 28.7542 | 28.3472 | 28.2656 | 28.3905 | 29.2393 | 29.3072 | 29.2717 | 27.8366 | 27.9248 | 27.8350 |
| GmGF14l | 28.7118 | 28.7185 | 28.6424 | 27.1707 | 27.1983 | 27.2522 | 30.0493 | 30.0472 | 30.0598 | 28.5743 | 28.5005 | 28.5633 |
| GmGF14m | 33.6778 | 33.7411 | 33.5887 | 32.8524 | 32.6818 | 32.6213 | 35.1595 | 35.3038 | 35.1817 | 33.3297 | 33.3255 | 33.4024 |
| GmGF14n | 28.7244 | 28.6601 | 28.5888 | 27.2589 | 27.1821 | 27.2540 | 29.1879 | 29.1784 | 29.2670 | 28.5110 | 28.3854 | 28.5458 |
| GmGF14o | 33.8014 | 33.6691 | 33.8791 | 33.9363 | 33.9583 | 34.1302 | 35.2936 | 35.2427 | 35.3931 | 33.8520 | 33.7497 | 33.6837 |
| GmGF14p | 28.8060 | 28.6364 | 28.6954 | 28.8377 | 29.0712 | 28.8959 | 29.8936 | 29.7464 | 29.8818 | 29.5270 | 29.5180 | 29.4354 |
| GmGF14q | 28.6165 | 28.6634 | 28.7054 | 27.4934 | 27.5418 | 27.5212 | 30.1030 | 30.2667 | 30.0971 | 28.5213 | 28.3949 | 28.5477 |
| GmGF14r | 28.5863 | 28.6245 | 28.6752 | 27.8577 | 27.8505 | 27.7996 | 30.3242 | 30.2719 | 30.4947 | 29.0192 | 29.2024 | 29.1924 |
| GmGF14t | 33.5219 | 33.6706 | 33.8885 | 32.0255 | 31.9020 | 32.0378 | 34.9730 | 34.9083 | 35.0664 | 34.0395 | 34.0505 | 34.2335 |
